# Can laboratory test-based frailty indices contribute to frailty screening in emergency departments?

**DOI:** 10.1093/ageing/afaf192

**Published:** 2025-07-16

**Authors:** Hugh Logan Ellis, Liam Dunnell, Julie Whitney, Cara Jennings, Dan Wilson, Jane Tippett, James Teo, Zina Ibrahim, Kenneth Rockwood

**Affiliations:** Department of Biostatistics & Health Informatics, Social Genetic and Developmental Psychiatry Centre, King’s College London, Memory Lane, Southwark, London SE5 8AF, UK; Department of Medicine, Dalhousie University, Suite 1421-5955 Veterans’ Memorial Lane, Halifax, Nova Scotia B3H 4R2, Canada; University Hospital Lewisham, Lewisham, London, UK; School of Life Course & Population Sciences, King’s College London, London, UK; Emergency Department, King’s College Hospital NHS Foundation Trust, London, UK; Department of Clinical Gerontology, King’s College Hospital NHS Foundation Trust, Denmark Hill, London SE5 9RS, UK; Emergency Department, King’s College Hospital NHS Foundation Trust, London, UK; Neurology Department, King’s College Hospital NHS Foundation Trust, London, UK; Department of Biostatistics & Health Informatics, King’s College London, London, UK; Department of Medicine, Dalhousie University, Suite 1421-5955 Veterans’ Memorial Lane, Halifax, Nova Scotia B3H 4R2, Canada

**Keywords:** frailty screening, laboratory-based frailty index, emergency department, clinical frailty scale, reliability, older people

## Abstract

**Background:**

Laboratory-based frailty indices (FI-Labs) offer potential adjuncts and alternatives to clinical assessments. Still, their optimal configuration and construct validity compared with nurse-assessed Clinical Frailty Scale (CFS) scores remain unclear.

**Methods:**

In this retrospective cohort study, we evaluated five FI-Lab configurations against nurse-assessed CFS scores using data from 74 493 emergency department visits. We examined their association with clinical outcomes and assessed measurement reliability using mixed effects models.

**Results:**

While nurse assessments demonstrated superior outcome discrimination (c-statistic 0.726 for 90-day mortality versus 0.718 for best FI-Lab), automated FI-Lab measures showed significantly greater between-visit reliability [intraclass correlation coefficient (ICC) = 0.51–0.76 versus 0.37 for nurse CFS]. The drug-adjusted FI-Lab demonstrated highest reliability (ICC = 0.76) but weaker age associations (β = 0.002, *P* = .08) compared to other configurations (β = 0.006–0.013, *P* < .001). In complex models adjusting for illness severity, nurse CFS scores showed stronger mortality associations (HR 1.55, 95% CI 1.45–1.66 per standard deviation) compared to FI-Lab configurations (HR range 1.19–1.29). Notably, all frailty measures showed effect sizes comparable to age (HR range 1.37–1.55 per SD).

**Conclusions:**

Automated FI-Lab measures offer a reliability advantage over nurse-assessed CFS scores despite slightly lower predictive validity for mortality. Their comparable effect sizes to age suggest these automated measures capture clinically meaningful patient characteristics. This trade-off between reliability and predictive validity suggests that integrated approaches combining automated screening with targeted clinical assessment may provide optimal frailty identification in emergency settings.

## Key Points

- Automated laboratory-based frailty indices (FI-Labs) show superior between-visit reliability compared to nurse-assessed Clinical Frailty Scale scores in emergency departments and require no additional instrumentation.
- Nurse assessments demonstrate slightly better outcome discrimination but are strongly associated by measures of acute illness National Early Warning Score and frailty indices and presenting complaints.
- Drug-adjusted FI-Labs demonstrated highest reliability but had limited coverage due to frequently missing medication data.
- Novel laboratory shelf-life imputation methodology enables the capture of both acute and chronic health status components from routine blood tests.
- Integration of automated FI-Labs with targeted clinical assessment may provide the optimal approach to frailty identification in emergency settings.

## Introduction

**Figure 1:**
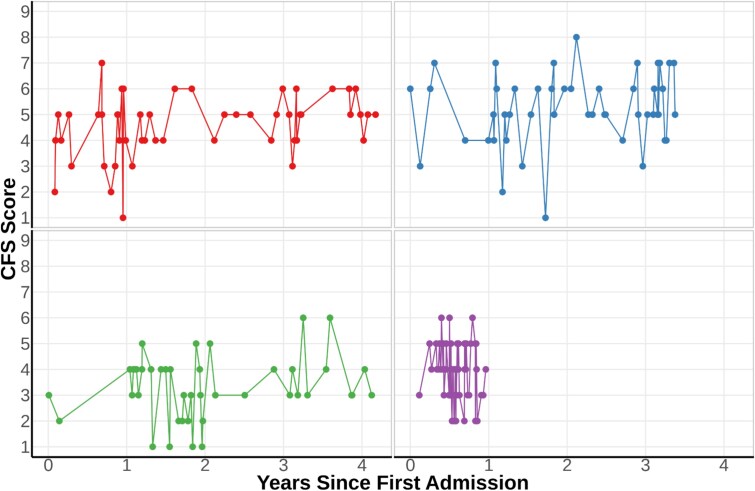
Marked instability in CFS scores in frequently admitted patients. This plot illustrates the substantial visit-to-visit variability in nurse-assessed CFS scores for four patients with the highest number of ED attendances. Note the upper right patient’s scores range from 1 (very fit) to 8 (very severely frail) within just a few months—a fluctuation inconsistent with our understanding of frailty as a relatively stable concept. This suggests nurse assessments may be capturing acute illness severity alongside baseline frailty status, potentially explaining their strong correlation with outcomes despite lower between-visit reliability.

In everyday practice, the Clinical Frailty Scale (CFS) [1, 2] is increasingly used for frailty screening in emergency departments (EDs). However, there are concerns regarding the consistency and reliability of CFS scores in these busy clinical environments [3, 4]. This variability can be seen in (Fig. 1). Acute illness severity and presenting complaints may influence nurse-assessed CFS scores, limiting their reliability as accurate indicators of underlying frailty [5]. This risks diminishing the clinical value of the CFS screening to detect baseline frailty.

Frailty is associated with adverse patient outcomes: increased likelihood of admission [6–9], prolonged length of stay [6, 10] and mortality [6, 9–16]. Prompt recognition and accurate estimation of frailty severity are important for optimising care [17] and initiating timely interventions that might mitigate its impact [15, 18–21]. As a result, frailty screening is considered a priority for geriatric medicine in EDs [22], which often serve as a primary contact point for many patients living with frailty. In 2019, NHS England outlined that, within 30 minutes of arrival to acute hospital services, all patients over 65 years old should undergo frailty screening [17]. This is challenging in EDs that are under ever-increasing pressures [23].

Automation of frailty assessments could enhance reliability and reduce demands on staff. Laboratory-based frailty indices (FI-Labs), utilising routinely available laboratory data, represent a promising automated approach. These indices show potential for predicting adverse outcomes, including mortality, prolonged hospitalisation and reduced likelihood of discharge [24–28]. An automated FI-Lab approach may be particularly suited to EDs given its minimal need for additional resources and equipment, using data already collected as part of routine clinical care.

The optimal configuration of FI-Labs and their relationship with traditional, nurse-based CFS assessments remain unclear. Our primary objectives were therefore as follows:

1. to compare the performance and reliability of FI-Labs with nurse-assessed CFS scores among older adults presenting to EDs and
2. to evaluate how different configurations of FI-Lab influence their associations with adverse clinical outcomes

## Methods

### Study design and setting

We conducted a retrospective cohort study of ED attendances between July 2017 and December 2021 at two London hospitals: King’s College Hospital is an inner-city tertiary centre with major trauma services, while Princess Royal University Hospital is a suburban district general hospital. Extending our analysis of frailty assessment methods [5], we evaluated both the predictive validity and between-visit reliability of six automated approaches, compared with nurse-assessed CFS scores. We adhered to the Strengthening the Reporting of Observational Studies in Epidemiology guidelines (see Supplementary Material for checklist).

### Population

The study included all ED attendances for patients aged 70 years or older. Not all patients had a CFS recorded. We excluded patients with indeterminate gender and those without complete attendance records (70 patients in total). For analyses requiring longitudinal data, we included only patients with multiple ED attendances during the study period.

### Laboratory data processing

For each patient, we extracted all laboratory results from the hospital electronic health record (EHR) spanning from 3 years before the study period until its conclusion. We included only laboratory tests that had associated reference ranges and were performed at least 1000 times during the study period; we did not include blood gas or urinalysis or primary care investigations as they did not meet these criteria. To manage temporal variations and extreme values, we aggregated laboratory results by calendar month, calculating mean values for each feature within these periods. To distinguish between baseline health status and acute presentation, we excluded all laboratory data from the calendar month of admission when calculating FI-Lab scores.

### Frailty assessment methods

Although several ED studies have used an FI-Lab, there has been variability in how the FI-Lab has been constructed [24, 29]. We compared seven approaches to quantifying frailty (summarised in supplemental table one):

1. **Base FI-Lab**: Using a 36-month observation window, we calculated the proportion of laboratory values outside their reference ranges. Features were included if they had measurements in at least three different months within the observation period.
2. **Short-period FI-Lab**: Similar to the base configuration, but only using the last 12 months of data.
3. **Mean-type FI-Lab**: Using the 36-month window but determining abnormality based on whether the mean value across all months fell outside reference ranges, rather than the proportion of abnormal months.
4. **High-features FI-Lab**: Required measurements in at least 10 different months, assessing the impact of increased data density requirements.
5. **Low-features FI-Lab**: Required measurements in only 1 month, maximising population coverage while potentially sacrificing reliability.
6. **Drug-adjusted FI-Lab**: Built upon the base FI-Lab by incorporating medication data. We used medication reconciliation records from ED presentations as the primary source of medication data, falling back to discharge prescriptions from previous admissions when reconciliation data was unavailable. This captured regular medications and any ad-hoc medications prescribed and documented at discharge or reconciliation. Medications were classified using Anatomical Therapeutic Chemical codes [30] into 21 categories. The FI-Lab denominator was increased by 21, and the numerator was increased by the number of different medication categories prescribed.
7. **Nurse-assessed CFS**: Recorded during ED triage following standard CFS guidelines.

**Figure 2:**
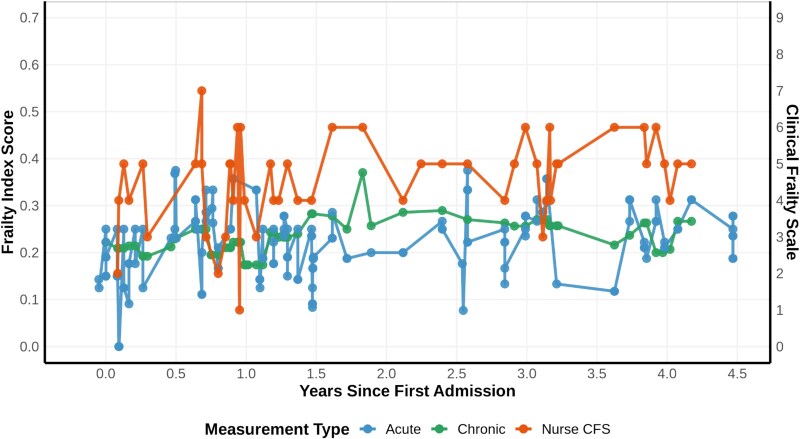
Longitudinal comparison of frailty measures across multiple hospital visits for a single patient. This figure demonstrates the stability of three different frailty assessment methods (acute FI-Lab in blue, Chronic FI-Lab in green and nurse-assessed CFS in orange) tracked over 4.5 years. Note the higher visit-to-visit variability in nurse-assessed CFS scores compared to the relatively more stable trajectory of chronic FI-Lab measurements*.*

**Figure 3:**
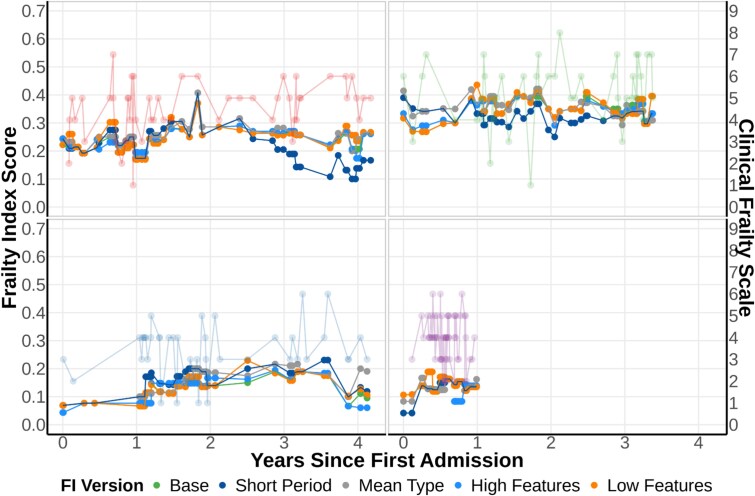
Comparison of stability across five FI-Lab configurations for four patients with frequent emergency department attendances. This visualisation demonstrates the between-visit reliability of different FI-Lab configurations (Base, Short Period, Mean Type, High Features and Low Features) plotted alongside nurse-assessed CFS scores (shown in pale colours on the right axis). Note the substantially higher stability of automated measures compared to nurse assessments, with all FI-Lab versions showing more gradual and consistent trajectories over time. This illustrates the key finding that automated measures capture a more stable underlying patient state while nurse assessments fluctuate more with acute presentation.

While these FI-Lab measures captured chronic health status, we separately analysed the acute clinical presentation using our FI-acute methodology [5]. For this acute measure, we assigned each laboratory feature a ‘shelf-life’ based on its median sampling interval. This resulted in feature-specific validity periods ranging from ~1 day for frequently sampled tests (e.g. electrolytes) to 3 months for more stable measures like HbA1c. Using these shelf-lives, we imputed the most recent valid result for each feature, considering only values that remained valid within 24 hours of admission. The FI-acute score was calculated using all available valid results, requiring a minimum of 10 features for score generation and accounted for the temporal relevance of different tests. Figure 2 illustrates the longitudinal stability of these different frailty assessment approaches, demonstrating the relative consistency of chronic FI-Lab measures. Figure 3 shows how adjusting the recipe affects the chronic FI-lab trajectory.

**Table 1:** Baseline characteristics and demographics of emergency department attendances stratified by frailty assessment method.

Column 1	Base	Short period	Mean	High features	Low features	Nurse	Drug
Total visits	60 381	60 381	60 381	36 267	74 493	60 381	60 381
Unique patients	23 956	23 956	23 956	12 524	31 353	23 956	23 956
Number of unique patients still alive at end of study period (%)	16 964 (70.8)	16 964 (70.8)	16 964 (70.8)	8750 (69.9)	22 591 (72.1)	16 964 (70.8)	16 964 (70.8)
Female of unique (%)	13 192 (55.1)	13 192 (55.1)	13 192 (55.1)	6832 (54.6)	17 541 (55.9)	13 192 (55.1)	1 192 (55.1)
Female of visits (%)	32 260 (53.4)	32 260 (53.4)	32 260 (53.4)	19 251 (53.1)	40 496 (54.4)	32 260 (53.4)	32 260 (53.4)
Ethnicity (%)							
Asian	2482 (4.1)	2482 (4.1)	2482 (4.1)	1722 (4.7)	2909 (3.9)	2482 (4.1)	2482 (4.1)
Black	10 894 (18.0)	10 894 (18.0)	10 894 (18.0)	8496 (23.4)	12 014 (16.1)	10 894 (18.0)	10 894 (18.0)
Mixed	33 (0.1)	33 (0.1)	33 (0.1)	23 (0.1)	40 (0.1)	33 (0.1)	33 (0.1)
Not stated	4263 (7.1)	4263 (7.1)	4263 (7.1)	1979 (5.5)	7058 (9.5)	4263 (7.1)	4263 (7.1)
Other	346 (0.6)	346 (0.6)	346 (0.6)	278 (0.8)	387 (0.5)	346 (0.6)	346 (0.6)
White	42 363 (70.2)	42 363 (70.2)	42 363 (70.2)	23 769 (65.5)	52 085 (69.9)	42 363 (70.2)	42 363 (70.2)
PRUH (%)	22 331 (37.0)	22 331 (37.0)	22 331 (37.0)	6714 (18.5)	30 687 (41.2)	22 331 (37.0)	22 331 (37.0)
Alive = TRUE (%)	40 070 (66.4)	40 070 (66.4)	40 070 (66.4)	24 103 (66.5)	49 923 (67.0)	40 070 (66.4)	40 070 (66.4)
Age (visits) [mean (SD)]	81.64 (7.01)	81.64 (7.01)	81.64 (7.01)	81.30 (6.88)	81.73 (7.11)	81.64 (7.01)	81.64 (7.01)
NEWS score [mean (SD)]	1.96 (1.31)	1.96 (1.31)	1.96 (1.31)	1.98 (1.33)	1.95 (1.30)	1.96 (1.31)	1.96 (1.31)
IMD decile [mean (SD)]	5.26 (2.63)	5.26 (2.63)	5.26 (2.63)	4.69 (2.37)	5.39 (2.66)	5.26 (2.63)	5.26 (2.63)
Length of hospital stay (days) [mean (SD)]	4.28 (9.75)	4.28 (9.75)	4.28 (9.75)	4.13 (9.67)	4.37 (9.96)	4.28 (9.75)	4.28 (9.75)
Chronic FI-Lab score [mean (SD)]	0.17 (0.13)	0.17 (0.13)	0.19 (0.14)	0.15 (0.13)	0.19 (0.13)	0.17 (0.13)	0.17 (0.13)
Acute FI-Lab score [mean (SD)]	0.39 (0.11)	0.39 (0.11)	0.39 (0.11)	0.39 (0.11)	0.39 (0.11)	0.39 (0.11)	0.39 (0.11)
CFS (adjusted) [mean (SD)]	4.06 (1.87)	4.06 (1.87)	4.06 (1.87)	4.20 (1.85)	4.01 (1.87)	4.06 (1.87)	4.06 (1.87)

Data shown for seven different assessment approaches: Base FI-Lab, Short period FI-Lab, Mean-type FI-Lab, High features FI-Lab, Low features FI-Lab, Nurse assessment and Drug-adjusted FI-Lab. Values are presented as counts (percentages) for categorical variables and mean (standard deviation) for continuous variables. Notable differences in sample sizes reflect varying data requirements across assessment methods, ranging from 36 267 visits for high-features configuration to 74 493 visits for low-features configuration. PRUH = Princess Royal University Hospital; IMD = Index of Multiple Deprivation.

### Data collection

Using the CogStack ecosystem for EHR processing, we extracted the following data.

- Demographic data: age, sex, ethnicity, postcode
- Clinical observations: vital signs, National Early Warning Score (NEWS)
- Laboratory results within measurement windows
- Medication records
- Presenting complaints (categorised into clinical domains)
- Outcomes: length of stay, mortality, readmissions

### Statistical analysis

We took two main approaches to evaluate the performance and reliability of the different frailty measures. First, with survival analyses, we assessed the predictive validity of each measure for key clinical outcomes. We examined four time-to-event outcomes: time to discharge from hospital, death during admission, death within 90 days of admission and overall survival. For each outcome, we constructed Cox proportional hazards models with two levels of complexity. Our basic models adjusted for fundamental demographic and institutional factors (age, sex and hospital site), whereas our complex models incorporated additional clinical variables including illness severity (NEWS score), socioeconomic status and acute FI-Lab. To facilitate comparison between different frailty measures, we standardised all hazard ratios to reflect the risk associated with a one standard deviation increase in each measure. We assessed model discrimination using c-statistics. Participants with missing data for any covariates included in the complex models were excluded from those specific analyses.

To evaluate the reliability of frailty measurements over time, we employed linear mixed effects models. These models accounted for the clustered nature of our data, with multiple ED visits nested within individual patients, by including random intercepts for patient identification. The fixed effects portion of our models included age (allowing us to capture both baseline differences and the passage of time as individuals aged during the study period), sex, hospital site and presenting complaint, with ‘generalised weakness’ serving as our reference category for presenting complaints [5] we adjusted for acute illness severity by incorporating NEWS and FI-Acute. We assessed the performance of these models through several complementary metrics. The intraclass correlation coefficient (ICC) provided a measure of between-visit reliability, while conditional and marginal R^2^ values helped us understand the proportion of variance explained by both fixed and random effects. We compared model fit using Akaike information criteria (AIC) and Bayesian information criteria.

All analyses were performed using R version 4.1.2. Statistical significance was set at *P* < .05 without correction for multiple comparisons.

### Ethical considerations

We obtained ethical approval from the King’s Electronic Patient Record Interface committee (approval ID 20230411B).

**Table 2:** Performance metrics of different frailty assessment methods for predicting clinical outcomes.

FI-Lab version	Sample size	ICC	90-day mortality HR	CI	C-statistic	LoS	Length of stay CI	C-statistic	Read-mission	Readmission CI	C-statistic	Overall survival	C-statistic
Base FI-Lab	60 381	0.60	1.25	(1.18–1.33)	0.701	0.87	(0.86–0.89)	0.622	1.05	(1.03–1.07)	0.581	1.27	0.694
Short-period FI-Lab	60 381	0.60	1.2	(1.14–1.26)	0.699	0.88	(0.87–0.90)	0.618	1.04	(1.02–1.06)	0.577	1.22	0.692
Mean-type FI-Lab	60 381	0.59	1.29	(1.22–1.37)	0.718	0.86	(0.85–0.88)	0.625	1.06	(1.04–1.08)	0.584	1.3	0.712
High-features FI-Lab	36 267	0.67	1.24	(1.16–1.33)	0.702	0.87	(0.85–0.89)	0.623	1.05	(1.02–1.08)	0.582	1.25	0.695
Low-features FI-Lab	74 493	0.51	1.19	(1.13–1.26)	0.694	0.84	(0.83–0.85)	0.629	1.03	(1.01–1.05)	0.573	1.21	0.686
Drug-adjusted FI-Lab	39 748	0.76	1.24	(1.17–1.31)	0.7	0.87	(0.85–0.89)	0.621	1.04	(1.02–1.06)	0.58	1.26	0.693
Nurse CFS	60 381	0.37	1.55	(1.45–1.66)	0.726	0.8	(0.78–0.81)	0.638	1.06	(1.04–1.09)	0.586	1.53	0.72

Comparison of sample sizes, reliability (ICC) and predictive validity for various clinical outcomes (hazard ratios with confidence intervals and c-statistics) across different frailty assessment methods.

*Note*: All hazard ratios standardised to reflect risk associated with one standard deviation increase in each measure.

## Results

### Patient characteristics

The analysis included 74 493 ED attendances from 54 075 unique patients aged ≥70 years (Table 1). Sample sizes varied by frailty measure due to differing minimum feature requirements: high-features configuration had the smallest sample (36 267 visits) while low-features had largest (74 493 visits). Nurse assessment was matched to the drug cohort for comparison in 39 748 visits. Median age was 81.9 years (IQR 76–87), with 54.3% female attendance.

### Reliability analysis

Drug-adjusted FI-Lab scores demonstrated highest between-visit reliability (ICC = 0.76, 95% CI 0.75–0.77), followed by high-features FI-Lab (ICC = 0.67, 95% CI 0.66–0.69) and base FI-Lab (ICC = 0.60, 95% CI 0.59–0.62). Nurse assessments showed lower reliability (ICC = 0.37, 95% CI 0.36–0.39), with 51.5% of variance attributable to visit-specific factors, such as the chief presenting complaint or NEWS.

Fixed effects analysis from the complex mixed-effects models revealed consistent patterns across automated measures. Age showed positive associations with most FI-Lab versions (standardised β range 0.006 to 0.010, all *P* < .001), while the drug-adjusted FI showed a slight negative association (β = −0.003, *P* = .028). Female sex associated with lower FI-Lab scores (β range − 0.04 to −0.02, all *P* < .001) but was not significantly associated with nurse assessments in the adjusted model (β = 0.05, *P* = .11).

### Association with mortality

In complex models adjusting for illness severity (including NEWS score and the FI-acute score), the nurse-assessed CFS was most strongly associated with 90-day mortality (HR 1.55 per SD, 95% CI 1.45–1.66). Among the chronic automated indices, the mean-type FI-Lab demonstrated the best performance (HR 1.29 per SD, 95% CI 1.22–1.37), followed by the base FI-Lab (HR 1.25, 95% CI 1.18–1.33). Notably, within these adjusted models, the FI-acute score itself was also consistently and independently associated with mortality outcomes; (HR range approx. 1.18–1.41 across models and outcomes, *P* < .001). Effect sizes for the primary chronic FI-Lab and age were comparable (age HR for 90-day mortality 1.37 per SD, 95% CI 1.34–1.40).

### Length of stay and readmission

All measures significantly associated with extended length of stay. Nurse assessment showed the strongest association (HR for discharge 0.80 per SD increase, 95% CI 0.78–0.81), followed by low-features FI-Lab (HR 0.84, 95% CI 0.83–0.85). Readmission risk similarly was higher with higher frailty scores across all measures (HR range 1.03–1.06 per SD increase).

These reliability and outcome associations are shown in Table 2.

### Clinical factors

Presenting complaint associated with nurse scores but not automated measures. Compared to ‘generalised weakness’, ‘difficulty breathing’ was associated with higher CFS score (β = 0.55, *P* < .01) and ‘chest pain’ lower (β = −0.22, *P* < .05). Higher NEWS scores correlated with higher CFS scores (β = 0.12, *P* < .001) but not chronic FI-Labs.

### Model fit comparison

Information criteria favoured automated measures (drug-adjusted AIC = −17 869; low-features AIC = −39 477) over nurse assessment (AIC = 61 580), reflecting greater consistency in automated scoring. However, nurse assessment showed superior discrimination for clinical outcomes (c-statistic 0.726 for 90-day mortality versus 0.718 for best FI-Lab), suggesting value in incorporating clinical judgement despite lower reliability.

## Discussion

Our FI-Labs showed inferior predictive validity compared to nurse-assessed CFS scores but exhibited greater between-visit reliability (Objective 1), while different FI-Lab configurations showed only minor variations in their associations with clinical outcomes, with increased complexity often resulted in reduced population coverage (Objective 2). Though not as strongly associated as nurse CFS scores, FI-Labs measures showed effect sizes comparable to age demonstrating they capture meaningful frailty gradations. Our findings support using an automated FI-Lab to improve frailty screening in EDs, extending our earlier work.

Automation in frailty screening is gaining significant attention, particularly through EHRs. Despite being in relatively early stages of development [31] numerous EHR-based models have emerged to automate frailty scoring [32–43]. The Electronic Frailty Index (eFI) [44] and Hospital Frailty Risk Score (HFRS) [45] and like measures have demonstrated alignment with traditional frailty measurements [31, 45, 46] and association with adverse health outcomes [31, 33, 34, 44, 47–53]. However, these approaches face substantial challenges with accuracy [54], data sharing across systems [55] and coding inconsistencies [35].

Traditional frailty indices, while valuable for their simplicity and implementation using existing data [55], often reach a ceiling in their predictive capability due to inherent limitations—primarily the reduction of complex physiological relationships to binary states and inability to capture temporal patterns. Brack and colleagues found the eFI to have a false positive rate of nearly 50%, despite being the best-performing tool examined, consequently increasing resource demands for accurate screening [54]. Furthermore, neither the eFI and HFRS [47] nor the HFRS and CFS [50], identify the same cohorts of patients with frailty, raising questions about accuracy and consistency. These models require sufficient data from EHRs, presenting challenges with cross-system data sharing [55], coding accuracy [35] and concern codes may miss certain features of frailty [35, 42]. While our hospital-derived FI-Lab differs from GP-based eFIs which use primary care data, future research comparing these across settings would be valuable.

Alternative approaches such as wearable sensors for gait and functional status analysis [56–62] or automated musculoskeletal [63–66] and physiological [67, 68] measurements, while promising, remain resource-intensive, limiting their widespread adoption. Recent advances in transformer-based architectures (a type of artificial intelligence model, as exemplified by the ‘T’ in GPT) suggest promising alternatives for capturing more nuanced interactions [69], but practical implementation across diverse healthcare settings presents significant challenges.

In contrast, an automated laboratory frailty index using routine investigations requires no additional instrumentation and has the potential to enhance accuracy of existing frailty indexes [70] or function as a stand-alone solution. The FI-Lab’s value lies in its minimal computational requirements and straightforward implementation, making it particularly suitable for healthcare systems with varying levels of technological maturity. Moloney and colleagues emphasise the importance of prioritising feasibility over creating ‘ideal’ screening instruments [71], a principle that FI-Lab embodies while still aligning with consensus requirements for frailty screening [71, 72].

We demonstrate that FI-Lab can help predict adverse outcomes in patients with frailty [24, 26] and FI-Labs may outperform the HFRS in predicting in-hospital mortality [28]. In the ED specifically, FI-Labs can predict increased mortality risk, longer length of stay, reduced likelihood of discharge home [24, 25] and increased readmission rates [24]. To our knowledge, this is the largest study examining FI-Lab use and its association with clinical outcomes [26, 29], strengthening existing literature advocating for incorporating FI-Lab into ED frailty screening [5, 24, 25, 27].

Our comparison of assessment methods revealed complementary strengths: nurse-assessed CFS scores demonstrated superior risk discrimination across clinical outcomes, while FI-Lab showed higher between-visit reliability. Despite studies indicating good reliability of CFS scoring in the ED [73, 74], automated measures appear to more consistently assess underlying frailty status, as nurse assessments may capture acute illness factors, explaining some observed variability. This suggests value in a combined approach. Chronic FI-Lab configurations reflect baseline health deficits with greater stability, while the FI-acute score provides a dynamic measure of current physiological disturbance. Using both alongside clinical assessment offers a more quantitative approach to characterising the ‘acute-on-chronic’ presentation, potentially distinguishing acute illness from underlying frailty. Neither fully explain age-related outcome variance, highlighting their individual limitations and reinforcing the benefit of using them in combination.

Comparisons between FI-Lab and CFS scoring have been reported [24, 75–79]. We offer a unique perspective on scoring consistency between automated FI-Lab and nurse-based CFS assessment. This is relevant to considering how the FI-Lab fits into clinical practice. It appears challenging to reliably predict clinical outcomes for acutely unwell patients with frailty through either automation or nurse-based assessment alone. While a combination approach would likely enhance accuracy [75], the ultimate goal of frailty screening in EDs is to optimise care pathways. Effective screening is essential in identifying patients who will benefit most from interventions aimed at reducing department time and improving outcomes [20]. Currently, the NHS ‘Getting It Right First Time’ guidance proposes frailty assessment for all over 65 years olds on arrival to hospital, with access to specialist care for those with a CFS score ≥ 6 [80].

Several limitations of our study warrant consideration. As a retrospective analysis of data from two London hospitals, our findings might not generalise to other healthcare settings. We cannot account for variations in laboratory reference ranges, clinical practices or population characteristics. Nevertheless, our results appear to align with findings and limitations described elsewhere [26, 29]. This study focused on the routinely documented CFS; other rapid measures like PRISMA-7 were unavailable in our EHR data for this retrospective analysis but warrant inclusion in future prospective comparisons.

Using observational data precludes causal inference; associations between frailty measures and outcomes should be interpreted cautiously. The availability of laboratory data varies across patient groups, presenting a significant methodological challenge. Different FI-Lab configurations captured distinct patient cohorts, potentially introducing selection bias—patients who are sicker are likely to have more comprehensive laboratory testing than those requiring minimal testing. This makes comparisons between FI-Lab configurations challenging: do observed differences reflect underlying population differences or methodological performance? This will require careful monitoring of test use, consistent with best practices in laboratory medicine. For further technical limitations, please see the supplemental materials.

The key question is straightforward: how do we screen more patients for frailty in the ED while ensuring the process remains effective? Getting it wrong carries real consequences—if we miss frailty, we miss opportunities for intervention; if we overstate it, we risk inappropriate care pathways. Our findings suggest the FI-Lab offers reliability in these assessments, reducing the risk of such errors.

To assess real-world impact, we need randomised trials to test whether FI-Lab actually improves patient care. We need to know whether helping to identify the right patients for intervention leads to better outcomes.

## Supplementary Material

aa-25-0992-File002_afaf192

aa-25-0992-File003_afaf192

## Data Availability

Due to patient confidentiality concerns, the raw data cannot be shared publicly. However, researchers interested in replicating or extending this work may contact the authors to discuss potential collaborations or data access arrangements, subject to appropriate ethical and governance approvals.
