# Supplementary material for: Can laboratory test-based frailty indices contribute to frailty screening in emergency departments?
Source: Age Ageing. 2025 Jul 16;54(7):afaf192. doi: 10.1093/ageing/afaf192 (PMC12264206; doi:10.1093/ageing/afaf192)
Supplement: aa-25-0992-File002_afaf192 [file aa-25-0992-file002_afaf192.pdf]

In [1]:

```
# 1. Setup & Configuration
## Libraries
library(tidyverse)
library(survival)
library(lme4)
library(survminer)
library(lubridate)
library(gridExtra)
## Global Settings
options(repr.plot.width = 20, repr.plot.height = 10)
custom_theme <- theme_minimal() +
                theme(text = element_text(size = 12))
```

```
Warning message in system("timedatectl", intern = TRUE):
“running command 'timedatectl' had status 1”
── Attaching packages ─────────────────────────────────────── tidyverse 1.3.2 ──
✓ ggplot2 3.4.0     ✓ purrr   1.0.0
✓ tibble  3.2.1     ✓ dplyr   1.1.2
✓ tidyr   1.2.1     ✓ stringr 1.5.0
✓ readr   2.1.3     ✓ forcats 0.5.2
── Conflicts ────────────────────────────────────────── tidyverse_conflicts() ──
✗ dplyr::filter() masks stats::filter()
✗ dplyr::lag()    masks stats::lag()
Loading required package: Matrix


Attaching package: ‘Matrix’


The following objects are masked from ‘package:tidyr’:

    expand, pack, unpack


Loading required package: ggpubr


Attaching package: ‘survminer’


The following object is masked from ‘package:survival’:

    myeloma


Loading required package: timechange


Attaching package: ‘lubridate’


The following objects are masked from ‘package:base’:

    date, intersect, setdiff, union



Attaching package: ‘gridExtra’


The following object is masked from ‘package:dplyr’:

    combine
```

In [2]:

```
#chronic fis - see separate supplemental for how these were created
filab_results <- read_csv("filab_results.csv")
# Drug scores (another supplemental)
drug_data <- read_csv('drug_count_admit_ma2.csv')
#See Logan Ellis et al 2025 Age and Aging paper for how these were created
combined_scores <- read_csv('combined_scores.csv')
#Creation in separate supplemental
fi_acute_df <- read_csv('filab_big4.csv', col_names = c('patient_TrustNumber', 'document_CreatedWhen', 'fi_acute')) %>%
left_join(combined_scores) %>%
select(fi_acute, patientVisit_AttendanceNumber) %>%
distinct()
```

```
Rows: 25862238 Columns: 7
── Column specification ────────────────────────────────────────────────────────
Delimiter: ","
chr  (3): client_idcode, n_months, type
dbl  (3): filab, n_tests, min_feature_count
dttm (1): year_month

ℹ Use `spec()` to retrieve the full column specification for this data.
ℹ Specify the column types or set `show_col_types = FALSE` to quiet this message.
Rows: 74830 Columns: 4
── Column specification ────────────────────────────────────────────────────────
Delimiter: ","
chr (2): patient_TrustNumber, clientvisit_visitidcode
dbl (2): drug_count, drug_count_ma

ℹ Use `spec()` to retrieve the full column specification for this data.
ℹ Specify the column types or set `show_col_types = FALSE` to quiet this message.
Rows: 136168 Columns: 47
── Column specification ────────────────────────────────────────────────────────
Delimiter: ","
chr  (19): _index, _id, patient_TrustNumber, patient_GenderCode, body_analys...
dbl  (18): ...1, imd_rank, imd_decile, score, adjusted_score, adjusted_score...
lgl   (4): _score, PRUH, Frailty_score, female
dttm  (6): patient_DOB, patient_DeceasedDtm, document_CreatedWhen, time, cfs...

ℹ Use `spec()` to retrieve the full column specification for this data.
ℹ Specify the column types or set `show_col_types = FALSE` to quiet this message.
Rows: 78970 Columns: 3
── Column specification ────────────────────────────────────────────────────────
Delimiter: ","
chr  (1): patient_TrustNumber
dbl  (1): fi_acute
dttm (1): document_CreatedWhen

ℹ Use `spec()` to retrieve the full column specification for this data.
ℹ Specify the column types or set `show_col_types = FALSE` to quiet this message.
Joining with `by = join_by(patient_TrustNumber, document_CreatedWhen)`
```

In [3]:

```
# Base configurations to test
fi_configs <- list(
  base = list(months = "last36", type = "prop_outside", min_features = 3),
  short_period = list(months = "last12", type = "prop_outside", min_features = 3),
  mean_type = list(months = "last36", type = "mean", min_features = 3),
  high_features = list(months = "last36", type = "prop_outside", min_features = 10),
  low_features = list(months = "last36", type = "prop_outside", min_features = 1)
)

# Function to generate FI for a specific configuration
generate_fi <- function(data, config) {
  data %>%
    filter(
      n_months == config$months,
      type == config$type,
      min_feature_count == config$min_features
    ) %>%
    rename(patient_TrustNumber = client_idcode, chron_filab = filab)
}

# Function to process and impute FI
process_fi <- function(fi_data) {
  # Create copy of data with shifted dates and NA values for imputation
  impute_template <- fi_data %>%
    mutate(year_month = year_month - months(1))

  # Combine with original data and fill
```

```
  impute_template %>%
    bind_rows(fi_data) %>%
    group_by(patient_TrustNumber) %>%
    arrange(year_month) %>%
    fill(chron_filab) %>%
    group_by(patient_TrustNumber, year_month) %>%
    slice(n())
}
# Generate all FI versions
fi_versions <- map(fi_configs, ~generate_fi(filab_results, .)) %>%
  map(process_fi)
```

In [4]:
```
combined_scores <- read_csv('combined_scores.csv') %>%
mutate(clientvisit_visitidcode = gsub("[[:punct:]]", "", patientVisit_AttendanceNumber)) %>%
left_join(fi_acute_df)
```

```
Rows: 136168 Columns: 47
── Column specification ──────────────────────────────────────────
Delimiter: ","
chr  (19): _index, _id, patient_TrustNumber, patient_GenderCode, body_analys...
dbl  (18): ...1, imd_rank, imd_decile, score, adjusted_score, adjusted_score...
lgl   (4): _score, PRUH, Frailty_score, female
dttm  (6): patient_DOB, patient_DeceasedDtm, document_CreatedWhen, time, cfs...

ℹ Use `spec()` to retrieve the full column specification for this data.
ℹ Specify the column types or set `show_col_types = FALSE` to quiet this message.
Joining with `by = join_by(patientVisit_AttendanceNumber)`
```

In [5]:
```
demo_data2 <- read_csv('demo_data2.csv')
discharge_times <- demo_data2 %>% filter(clientvisit_visitidcode %in% combined_scores$clientvisit_visitidcode) %>%
group_by(client_idcode) %>%
mutate(updatetime = max(updatetime)) %>%
group_by(client_idcode, clientvisit_visitidcode ) %>%
summarise(clientvisit_dischargedtm = first(clientvisit_dischargedtm), dod = first(client_deceaseddtm), updatetime = max(updatetime), clientvisit_admitdtm = first(clientvisit_admitdtm) )
```

```
Warning message:
“One or more parsing issues, call `problems()` on your data frame for details,
e.g.:
  dat <- vroom(...)
  problems(dat)”
Rows: 2399913 Columns: 13
── Column specification ──────────────────────────────────────────
Delimiter: ","
chr  (6): _index, client_idcode, client_gendercode, client_racecode, clienta...
dbl  (1): _id
lgl  (1): _score
dttm (5): client_dob, client_deceaseddtm, updatetime, clientvisit_admitdtm, ...

ℹ Use `spec()` to retrieve the full column specification for this data.
ℹ Specify the column types or set `show_col_types = FALSE` to quiet this message.
`summarise()` has grouped output by 'client_idcode'. You can override using the
`.groups` argument.
```

In [6]:
```
combined_scores_full <- combined_scores %>%
group_by(patientVisit_AttendanceNumber) %>%
arrange(desc(document_CreatedWhen)) %>%
slice(1L) %>%
left_join(drug_data) %>%
ungroup() %>%
mutate(
    time = document_CreatedWhen,
    clientvisit_visitidcode = gsub("[[:punct:]]", "", patientVisit_AttendanceNumber)) %>%
    group_by(clientvisit_visitidcode) %>%
    slice(1L) %>%
    mutate(client_idcode = patient_TrustNumber,
         year_month = floor_date( document_CreatedWhen - months(1), "month")) %>%
```

```r
    left_join(discharge_times, by = c('client_idcode', 'clientvisit_visitidcode')) %>%
    filter(!is.na(clientvisit_dischargedtm)) %>%
    arrange(time) %>%
    group_by(client_idcode) %>%
    mutate(
        Alive = is.na(patient_DeceasedDtm),
      last_update = max(max(time, na.rm = TRUE), max(updatetime, na.rm = TRUE)),
      dod = max(if_else( is.na(patient_DeceasedDtm), last_update, patient_DeceasedDtm)),
      died_ip = patient_DeceasedDtm < (clientvisit_dischargedtm + hours(24)),
      died_ip = if_else(is.na(died_ip), FALSE, died_ip),
      los = as.numeric(difftime(clientvisit_dischargedtm, clientvisit_admitdtm, units = "days")),
    days_elapsed = as.numeric(difftime(dod, document_CreatedWhen, units = "days")),
    days_censored = pmin(days_elapsed, 90),
      status_censored = ifelse(days_elapsed > 90, 0, !Alive))  %>%
    filter(los < 1000,
          days_elapsed >= 0,
          los >= 0)

combined_scores_full$age_scaled <- scale(combined_scores_full$age)
combined_scores_full$news_scaled <- scale(combined_scores_full$NEWS_Score)
combined_scores_full$fi_acute_scaled <- scale(combined_scores_full$fi_acute)
```

```
Joining with `by = join_by(patient_TrustNumber, clientvisit_visitidcode)`
```

In [7]:
```r
combined_scores_full$imd_scaled <- scale(combined_scores_full$imd_rank)
```

In [8]:
```r
combined_scores_full$news_scaled <- scale(combined_scores_full$NEWS_Score)
```

In [9]:
```r
combined_scores_full$fi_acute_scaled <- scale(combined_scores_full$fi_acute)
```

In [10]:
```r
generate_survival_datasets <- function(combined_data, fi_data) {
  # LOS and readmission dataset
  los_data <- combined_data %>%
      left_join(fi_data)  %>%
    filter(!is.na(chron_filab))

  # IP deaths - last visit per person
  ip_deaths <- los_data %>%
           group_by(client_idcode) %>%
           arrange(desc(time)) %>%
           slice(1L) %>%
           mutate(died_ip = if_else(los > 30, FALSE, died_ip),
                    los = min(los, 30))


  # 90-day mortality - first visit
  mort_90 <- los_data %>%
    group_by(client_idcode) %>%
    arrange(desc(time)) %>%
    slice(1L)


  # All mortality - first visit
  mort_all <- los_data %>%
    group_by(client_idcode) %>%
    arrange(time) %>%
    slice(1L)

  list(
    los = los_data,
    ip_death = ip_deaths,
    mort_90 = mort_90,
    mort_all = mort_all
```

```
  )
}
```

In [34]:

```
compare_fi_versions <- function(fi_list, outcomes_data) {
  # Prepare LOS dataset
  los_data <- outcomes_data %>%
    left_join(fi_list$base) %>%
    mutate(
      chron_fianddrug = if_else(!is.na(drug_count),
                                (chron_filab * n_tests + drug_count) / (21 + n_tests),
                                NA_real_)
    ) %>%
    filter(!is.na(chron_filab))

  # Generate additional datasets for nurse and drug configurations
  extra_data <- list(
    los = los_data,
    ip_death = los_data %>%
      group_by(client_idcode) %>%
      arrange(desc(time)) %>%
      slice(1L) %>%
      mutate(died_ip = if_else(los > 30, FALSE, died_ip),
             los = min(los, 30)),
    mort_90 = los_data %>%
      group_by(client_idcode) %>%
      arrange(desc(time)) %>%
      slice(1L),
    mort_all = los_data %>%
      group_by(client_idcode) %>%
      arrange(time) %>%
      slice(1L)
  )

  # Define model formulas
  basic_formula <- "~ scale(chron_filab) + age_scaled + female + PRUH"
  complex_formula <- "~ scale(chron_filab) + age_scaled + female + PRUH + news_scaled + imd_scaled + fi_acute_scaled"

  # Process the original FI configurations
  base_results <- map(fi_list, function(fi) {
    datasets <- generate_survival_datasets(outcomes_data, fi)

    # Basic models
    basic_models <- list(
      los = coxph(as.formula(paste0("Surv(los, !died_ip)", basic_formula)), data = datasets$los),
      ip_death = coxph(as.formula(paste0("Surv(los, died_ip)", basic_formula)), data = datasets$ip_death),
      mort_90 = coxph(as.formula(paste0("Surv(days_censored, status_censored)", basic_formula)), data = datasets$mort_90),
      mort_all = coxph(as.formula(paste0("Surv(days_elapsed, !Alive)", basic_formula)), data = datasets$mort_all)
    )

    # Complex models
    complex_models <- list(
      los = coxph(as.formula(paste0("Surv(los, !died_ip)", complex_formula)), data = datasets$los),
      ip_death = coxph(as.formula(paste0("Surv(los, died_ip)", complex_formula)), data = datasets$ip_death),
      mort_90 = coxph(as.formula(paste0("Surv(days_censored, status_censored)", complex_formula)), data = datasets$mort_90),
      mort_all = coxph(as.formula(paste0("Surv(days_elapsed, !Alive)", complex_formula)), data = datasets$mort_all)
    )

    surv_fits <- list(
      los = survfit(Surv(los, !died_ip) ~ ntile(chron_filab, 8), data = datasets$los),
      ip_death = survfit(Surv(los, died_ip) ~ ntile(chron_filab, 8), data = datasets$ip_death),
      mort_90 = survfit(Surv(days_censored, status_censored) ~ ntile(chron_filab, 8), data = datasets$mort_90),
      mort_all = survfit(Surv(days_elapsed, !Alive) ~ ntile(chron_filab, 8), data = datasets$mort_all)
    )

    list(
```

```r
    basic_models = basic_models,
    complex_models = complex_models,
    surv_fits = surv_fits,
    datasets = datasets
  )
})

# Define nurse formulas
nurse_basic_formula <- "~ scale(adjusted_score) + age_scaled + female + PRUH"
nurse_complex_formula <- "~ scale(adjusted_score) + age_scaled + female + PRUH + news_scaled + imd_scaled + fi_acute_scaled"

# Add nurse configuration with both model types
nurse_results <- list(
  basic_models = list(
    los = coxph(as.formula(paste0("Surv(los, !died_ip)", nurse_basic_formula)), data = extra_data$los),
    ip_death = coxph(as.formula(paste0("Surv(los, died_ip)", nurse_basic_formula)), data = extra_data$ip_death),
    mort_90 = coxph(as.formula(paste0("Surv(days_censored, status_censored)", nurse_basic_formula)), data = extra_data$mort_90),
    mort_all = coxph(as.formula(paste0("Surv(days_elapsed, !Alive)", nurse_basic_formula)), data = extra_data$mort_all)
  ),
  complex_models = list(
    los = coxph(as.formula(paste0("Surv(los, !died_ip)", nurse_complex_formula)), data = extra_data$los),
    ip_death = coxph(as.formula(paste0("Surv(los, died_ip)", nurse_complex_formula)), data = extra_data$ip_death),
    mort_90 = coxph(as.formula(paste0("Surv(days_censored, status_censored)", nurse_complex_formula)), data = extra_data$mort_90),
    mort_all = coxph(as.formula(paste0("Surv(days_elapsed, !Alive)", nurse_complex_formula)), data = extra_data$mort_all)
  ),
  surv_fits = list(
    los = survfit(Surv(los, !died_ip) ~ adjusted_scoref, data = extra_data$los),
    ip_death = survfit(Surv(los, died_ip) ~ adjusted_scoref, data = extra_data$ip_death),
    mort_90 = survfit(Surv(days_censored, status_censored) ~ adjusted_scoref, data = extra_data$mort_90),
    mort_all = survfit(Surv(days_elapsed, !Alive) ~ adjusted_scoref, data = extra_data$mort_all)
  ),
  datasets = extra_data
)

# Define drug formulas
drug_basic_formula <- "~ scale(chron_fianddrug) + age_scaled + female + PRUH"
drug_complex_formula <- "~ scale(chron_fianddrug) + age_scaled + female + PRUH + news_scaled + imd_scaled + fi_acute_scaled"

# Add drug configuration with both model types
drug_results <- list(
  basic_models = list(
    los = coxph(as.formula(paste0("Surv(los, !died_ip)", drug_basic_formula)), data = extra_data$los),
    ip_death = coxph(as.formula(paste0("Surv(los, died_ip)", drug_basic_formula)), data = extra_data$ip_death),
    mort_90 = coxph(as.formula(paste0("Surv(days_censored, status_censored)", drug_basic_formula)), data = extra_data$mort_90),
    mort_all = coxph(as.formula(paste0("Surv(days_elapsed, !Alive)", drug_basic_formula)), data = extra_data$mort_all)
  ),
  complex_models = list(
    los = coxph(as.formula(paste0("Surv(los, !died_ip)", drug_complex_formula)), data = extra_data$los),
    ip_death = coxph(as.formula(paste0("Surv(los, died_ip)", drug_complex_formula)), data = extra_data$ip_death),
    mort_90 = coxph(as.formula(paste0("Surv(days_censored, status_censored)", drug_complex_formula)), data = extra_data$mort_90),
    mort_all = coxph(as.formula(paste0("Surv(days_elapsed, !Alive)", drug_complex_formula)), data = extra_data$mort_all)
  ),
  surv_fits = list(
    los = survfit(Surv(los, !died_ip) ~ ntile(chron_fianddrug, 8), data = extra_data$los),
    ip_death = survfit(Surv(los, died_ip) ~ ntile(chron_fianddrug, 8), data = extra_data$ip_death),
    mort_90 = survfit(Surv(days_censored, status_censored) ~ ntile(chron_fianddrug, 8), data = extra_data$mort_90),
    mort_all = survfit(Surv(days_elapsed, !Alive) ~ ntile(chron_fianddrug, 8), data = extra_data$mort_all)
  ),
  datasets = extra_data
)

# Combine all results
c(base_results, list(nurse = nurse_results, drug = drug_results))
}
```

In [36]:
```
compare_fi_versions_o <- function(fi_list, outcomes_data) {
  # Prepare LOS dataset
  los_data <- outcomes_data %>%
    left_join(fi_list$base) %>%
    mutate(
      chron_fianddrug = if_else(!is.na(drug_count),
                                (chron_filab * n_tests + drug_count) / (21 + n_tests),
                                NA_real_)
    ) %>%
    filter(!is.na(chron_filab))

  # Generate additional datasets for nurse and drug configurations
  extra_data <- list(
    los = los_data,
    ip_death = los_data %>%
      group_by(client_idcode) %>%
      arrange(desc(time)) %>%
      slice(1L)%>%
       mutate(died_ip = if_else(los > 30, FALSE, died_ip),
                    los = min(los, 30)),
    mort_90 = los_data %>%
      group_by(client_idcode) %>%
      arrange(desc(time)) %>%
      slice(1L),
    mort_all = los_data %>%
      group_by(client_idcode) %>%
      arrange(time) %>%
      slice(1L)
  )

  # Process the original FI configurations
  base_results <- map(fi_list, function(fi) {
    datasets <- generate_survival_datasets(outcomes_data, fi)

    models <- list(
      los = coxph(Surv(los, !died_ip) ~ scale(chron_filab) + age_scaled + female + PRUH, data = datasets$los),
      ip_death = coxph(Surv(los, died_ip) ~ scale(chron_filab) + age_scaled + female + PRUH, data = datasets$ip_death),
      mort_90 = coxph(Surv(days_censored, status_censored) ~ scale(chron_filab) + age_scaled + female + PRUH, data = datasets$mort_90),
      mort_all = coxph(Surv(days_elapsed, !Alive) ~ scale(chron_filab) + age_scaled + female + PRUH, data = datasets$mort_all)
    )

    surv_fits <- list(
      los = survfit(Surv(los, !died_ip) ~ ntile(chron_filab, 8), data = datasets$los),
      ip_death = survfit(Surv(los, died_ip) ~ ntile(chron_filab, 8), data = datasets$ip_death),
      mort_90 = survfit(Surv(days_censored, status_censored) ~ ntile(chron_filab, 8), data = datasets$mort_90),
      mort_all = survfit(Surv(days_elapsed, !Alive) ~ ntile(chron_filab, 8), data = datasets$mort_all)
    )

    list(models = models, surv_fits = surv_fits, datasets = datasets)
  })

  # Add nurse configuration
  nurse_results <- list(
    models = list(
      los = coxph(Surv(los, !died_ip) ~ scale(adjusted_score) + age_scaled + female + PRUH, data = extra_data$los),
      ip_death = coxph(Surv(los, died_ip) ~ scale(adjusted_score) + age_scaled + female + PRUH, data = extra_data$ip_death),
      mort_90 = coxph(Surv(days_censored, status_censored) ~ scale(adjusted_score) + age_scaled + female + PRUH, data = extra_data$mort_90),
      mort_all = coxph(Surv(days_elapsed, !Alive) ~ scale(adjusted_score) + age_scaled + female + PRUH, data = extra_data$mort_all)
    ),
    surv_fits = list(
      los = survfit(Surv(los, !died_ip) ~ adjusted_scoref, data = extra_data$los),
      ip_death = survfit(Surv(los, died_ip) ~ adjusted_scoref, data = extra_data$ip_death),
      mort_90 = survfit(Surv(days_censored, status_censored) ~ adjusted_scoref, data = extra_data$mort_90),
      mort_all = survfit(Surv(days_elapsed, !Alive) ~ adjusted_scoref, data = extra_data$mort_all)
    ),
```

```
    datasets = extra_data
  )


  # Add drug configuration
  drug_results <- list(
    models = list(
      los = coxph(Surv(los, !died_ip) ~ scale(chron_fianddrug) + age_scaled + female + PRUH, data = extra_data$los),
      ip_death = coxph(Surv(los, died_ip) ~ scale(chron_fianddrug) + age_scaled + female + PRUH, data = extra_data$ip_death),
      mort_90 = coxph(Surv(days_censored, status_censored) ~ scale(chron_fianddrug) + age_scaled + female + PRUH, data = extra_data$mort_90),
      mort_all = coxph(Surv(days_elapsed, !Alive) ~ scale(chron_fianddrug) + age_scaled + female + PRUH, data = extra_data$mort_all)
    ),
    surv_fits = list(
      los = survfit(Surv(los, !died_ip) ~ ntile(chron_fianddrug, 8), data = extra_data$los),
      ip_death = survfit(Surv(los, died_ip) ~ ntile(chron_fianddrug, 8), data = extra_data$ip_death),
      mort_90 = survfit(Surv(days_censored, status_censored) ~ ntile(chron_fianddrug, 8), data = extra_data$mort_90),
      mort_all = survfit(Surv(days_elapsed, !Alive) ~ ntile(chron_fianddrug, 8), data = extra_data$mort_all)
    ),
      datasets = extra_data
  )

  # Combine all results at the same level
  c(base_results, list(nurse = nurse_results, drug = drug_results))
}
```

In [35]:
```
outputs2 <- compare_fi_versions(fi_versions, combined_scores_full)
```

```
Joining with `by = join_by(patient_TrustNumber, year_month)`
Joining with `by = join_by(patient_TrustNumber, year_month)`
Joining with `by = join_by(patient_TrustNumber, year_month)`
Joining with `by = join_by(patient_TrustNumber, year_month)`
Joining with `by = join_by(patient_TrustNumber, year_month)`
Joining with `by = join_by(patient_TrustNumber, year_month)`
```

In [37]:
```
create_survival_plots <- function(results, outcome_type) {
  # Plot settings based on outcome type
  plot_settings <- list(
    los = list(
      xlab = "Days in Hospital",
      ylab = "Probability of Remaining in Hospital",
      xlim = c(0, 30),
      break.time.by = 5
    ),
    ip_deaths = list(
      xlab = "Days",
      ylab = "Probability of Survival",
      xlim = c(0, 30),
      break.time.by = 5
    ),
    mort_90 = list(
      xlab = "Days",
      ylab = "Probability of Survival",
      xlim = c(0, 90),
      break.time.by = 15
    ),
    mort_all = list(
      xlab = "Days",
      ylab = "Probability of Survival",
      xlim = c(0, 730),
      break.time.by = 180
    )
  )

  # Create plots for each FI version
  plots <- imap(results, function(result, version) {
```

```r
    ggsurvplot(
      result$surv_fits[[outcome_type]],  # Now correctly accessing surv_fits
      data = result$datasets[[outcome_type]],  # Now correctly accessing datasets
      censor = FALSE,
      pval = FALSE,
      risk.table = FALSE,
      fontsize = 4,
      ggtheme = theme_minimal(),
      palette = "Set1",
      title = paste("Survival Plot -", version),
      xlab = plot_settings[[outcome_type]]$xlab,
      ylab = plot_settings[[outcome_type]]$ylab,
      xlim = plot_settings[[outcome_type]]$xlim,
      break.time.by = plot_settings[[outcome_type]]$break.time.by,
      legend.title = "Score Group",
      legend = "none"
    )$plot
  })

  # Convert list of plots to a list that grid.arrange can handle
  plot_list <- plots %>% unname()

  # Arrange plots in a grid
  do.call(grid.arrange, c(plot_list, ncol = 4))
}
```

In [38]:

```r
create_coefficient_plot <- function(results, outcome_type, model_type = "basic_models") {
  # Extract coefficients
  coefficients <- imap_dfr(results, function(result, version) {
    model <- result[[model_type]][[outcome_type]]
    broom::tidy(model, conf.int = TRUE) %>%
      mutate(
        version = version,
        HR = exp(estimate),
        HR_CI = sprintf("%.2f (%.2f-%.2f)",
                        exp(estimate),
                        exp(conf.low),
                        exp(conf.high))
      )
  })

  # Extract concordance
  metrics <- imap_dfr(results, function(result, version) {
    model <- result[[model_type]][[outcome_type]]
    concordance <- model$concordance
    c_stat <- concordance["concordant"] / (concordance["concordant"] + concordance["discordant"])
    data.frame(
      version = version,
      metric = sprintf("C-statistic: %f, N = %d, Events = %d",
                       c_stat,
                       model$n,
                       model$nevent)
    )
  })

  print(metrics)
  ggplot(coefficients, aes(y = term, x = HR, color = version)) +
    geom_point(position = position_dodge(width = 0.5)) +
    geom_errorbarh(aes(xmin = exp(conf.low), xmax = exp(conf.high)),
                   position = position_dodge(width = 0.5), height = 0.2) +
    geom_vline(xintercept = 1, linetype = "dashed") +
    geom_text(aes(label = HR_CI, x = 2),
              position = position_dodge(width = 0.8),
              hjust = 0) +
```

```
    geom_text(data = metrics,
              aes(label = metric, x = 0.6,
                  y = length(unique(coefficients$term))),
              hjust = 0,
              position = position_dodge(width = 1)) +
    scale_x_continuous(trans = "log10", limits = c(0.5, 2.2)) +
    labs(
      title = paste("Hazard Ratios -", outcome_type, "-",
                   ifelse(model_type == "basic_models", "Basic Model", "Complex Model")),
      x = "Hazard Ratio (95% CI)",
      y = "Variable"
    ) +
    theme_minimal()
}
```

In [39]:

```
# Run comparison function
results <- outputs2

# Define outcomes and model types
outcomes <- c("los", "ip_death", "mort_90", "mort_all")
model_types <- c("basic_models", "complex_models")

# Generate survival plots (these don't change with model type)
survival_plots <- map(outcomes, ~create_survival_plots(results, .))
names(survival_plots) <- outcomes

# Generate coefficient plots for both model types
coefficient_plots <- list(
  basic = map(outcomes, ~create_coefficient_plot(results, ., "basic_models")),
  complex = map(outcomes, ~create_coefficient_plot(results, ., "complex_models"))
)
names(coefficient_plots$basic) <- outcomes
names(coefficient_plots$complex) <- outcomes

# Create metrics table for both model types
create_metrics_table <- function(results, model_type = "basic_models") {
  imap_dfr(results, function(result, version) {
    map_dfr(result[[model_type]], function(model) {
      tibble(
        concordance = summary(model)$concordance,
        AIC = AIC(model),
        n_events = model$nevent,
        n_total = model$n
      )
    }, .id = "outcome") %>%
      mutate(
        version = version,
        model_type = model_type
      )
  })
}

# Generate metrics for both model types
metrics_tables <- map_dfr(model_types, ~create_metrics_table(results, .))
```

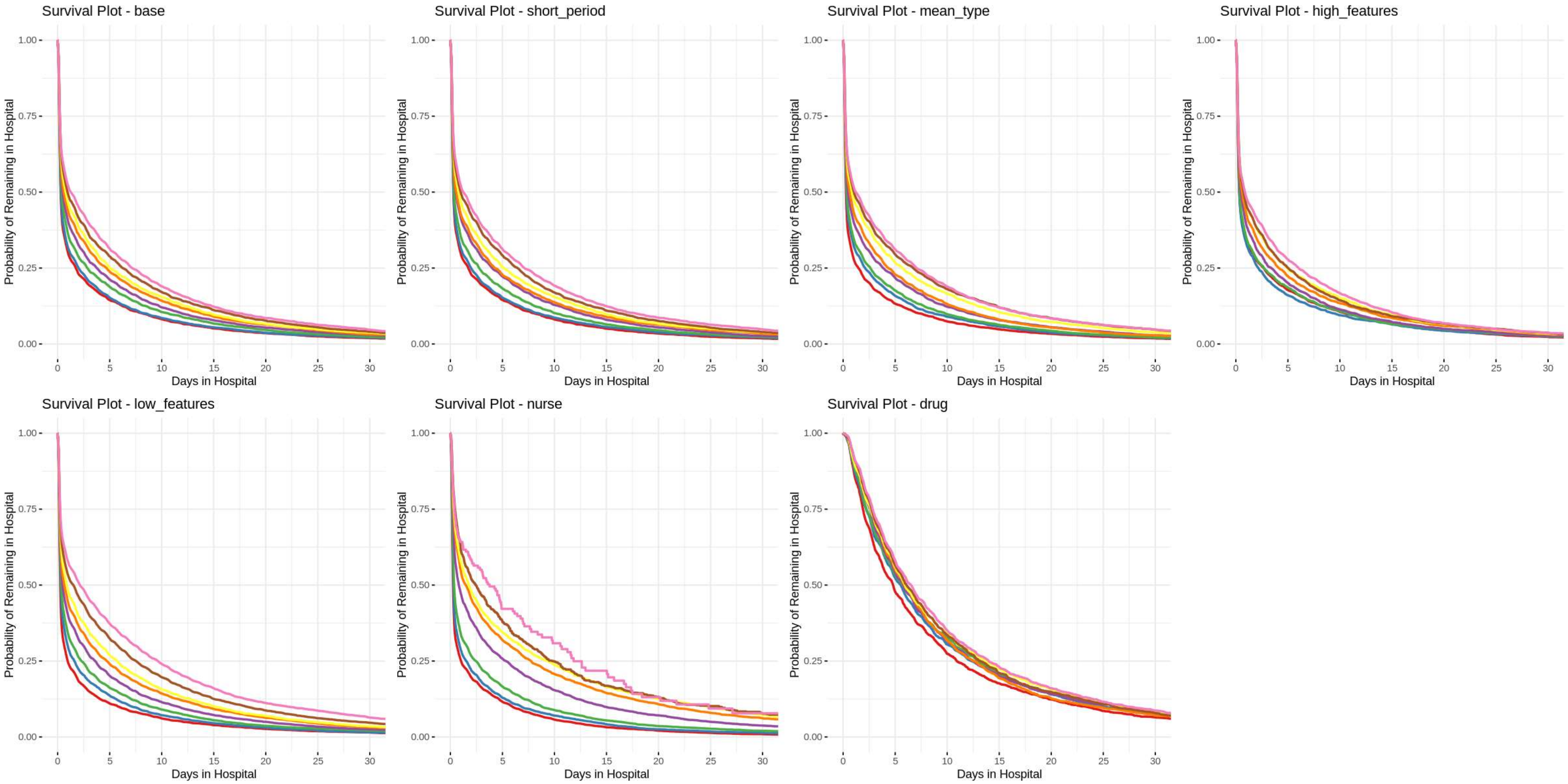
Survival Plot - base
Survival Plot - short_period
Survival Plot - mean_type
Survival Plot - high_features
Survival Plot - low_features
Survival Plot - nurse
Survival Plot - drug
Probability of Remaining in Hospital
Days in Hospital
1.00
0.75
0.50
0.25
0.00
0
5
10
15
20
25
30

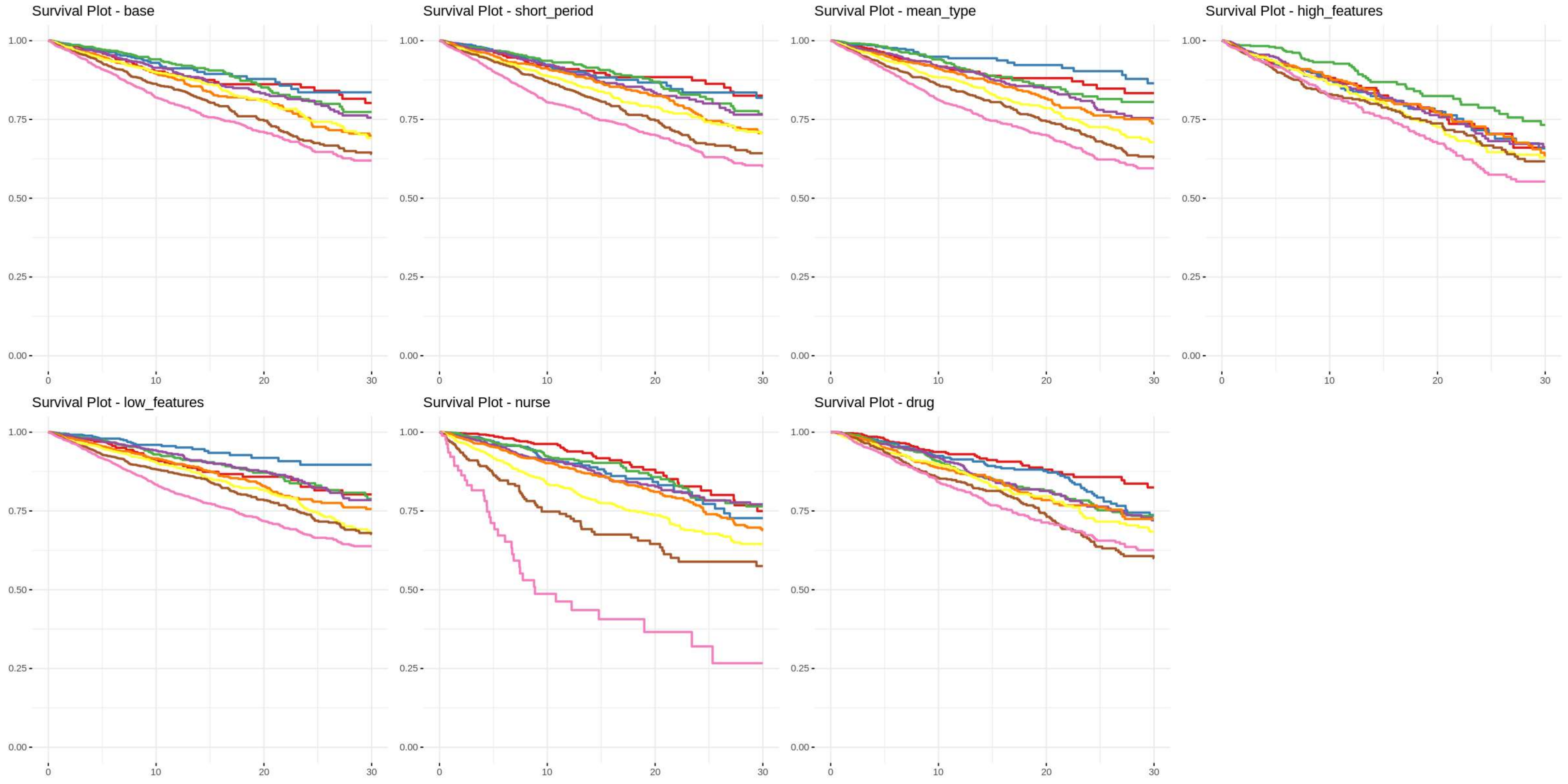
Survival Plot - base
Survival Plot - short_period
Survival Plot - mean_type
Survival Plot - high_features
Survival Plot - low_features
Survival Plot - nurse
Survival Plot - drug
1.00
0.75
0.50
0.25
0.00
0
10
20
30

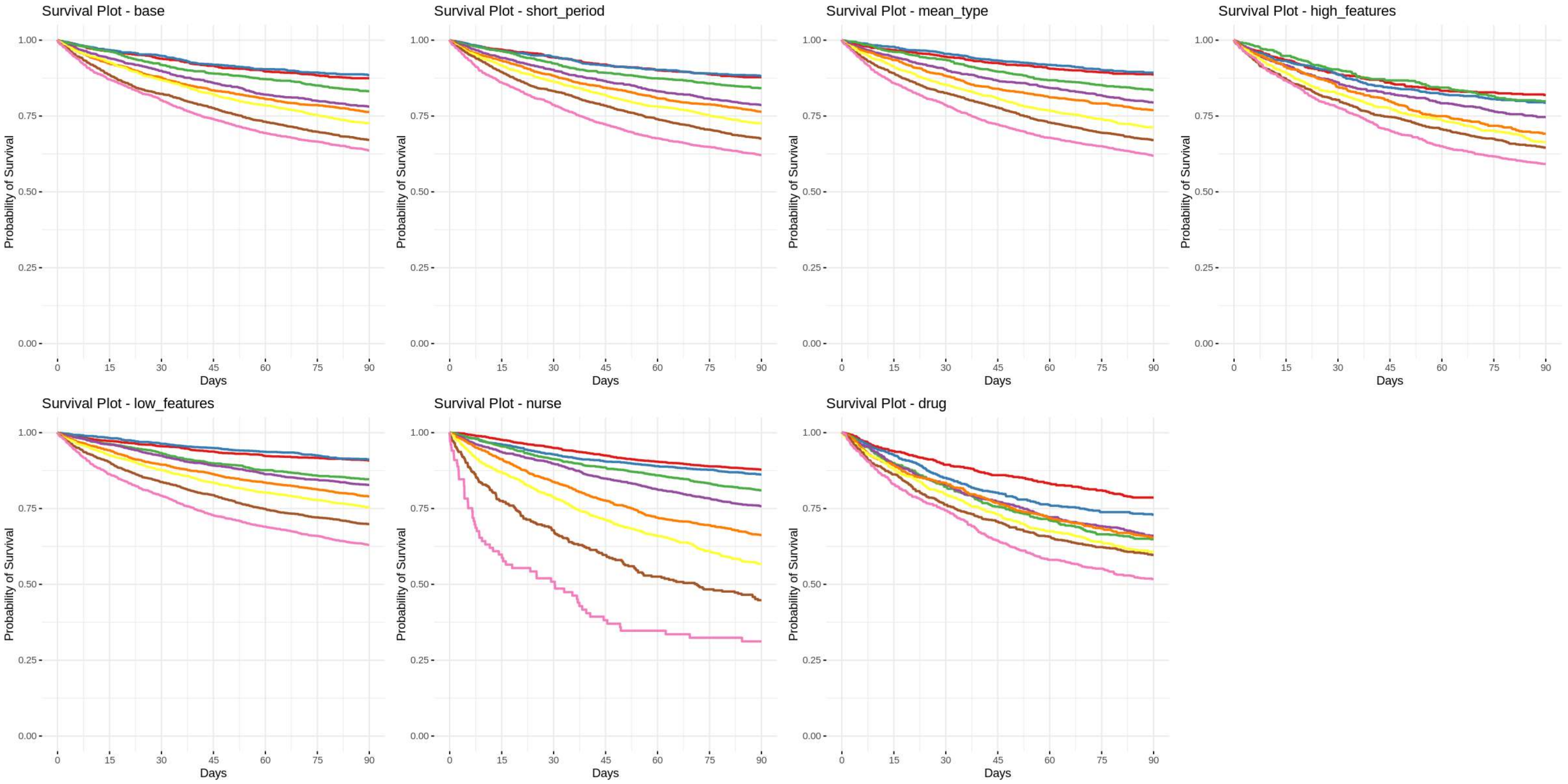
Survival Plot - base
Survival Plot - short_period
Survival Plot - mean_type
Survival Plot - high_features
Survival Plot - low_features
Survival Plot - nurse
Survival Plot - drug
Probability of Survival
1.00
0.75
0.50
0.25
0.00
0
15
30
45
60
75
90
Days

```
        version                                        metric
1          base C-statistic: 0.568753, N = 60381, Events = 59055
2  short_period C-statistic: 0.569558, N = 60381, Events = 59055
3     mean_type C-statistic: 0.571247, N = 60381, Events = 59055
4 high_features C-statistic: 0.559086, N = 36267, Events = 35359
5  low_features C-statistic: 0.588891, N = 74493, Events = 72921
6         nurse C-statistic: 0.603567, N = 39748, Events = 38897
7          drug C-statistic: 0.534056, N = 22146, Events = 21056
        version                                       metric
1          base C-statistic: 0.639390, N = 23956, Events = 1176
2  short_period C-statistic: 0.644756, N = 23956, Events = 1176
3     mean_type C-statistic: 0.651073, N = 23956, Events = 1176
4 high_features  C-statistic: 0.620535, N = 12524, Events = 808
5  low_features C-statistic: 0.646420, N = 31353, Events = 1394
6         nurse  C-statistic: 0.681738, N = 18420, Events = 742
7          drug   C-statistic: 0.654992, N = 8757, Events = 944
        version                                       metric
1          base C-statistic: 0.647744, N = 23956, Events = 3392
2  short_period C-statistic: 0.651602, N = 23956, Events = 3392
3     mean_type C-statistic: 0.658382, N = 23956, Events = 3392
4 high_features C-statistic: 0.617053, N = 12524, Events = 2085
5  low_features C-statistic: 0.671458, N = 31353, Events = 4009
6         nurse C-statistic: 0.679577, N = 18420, Events = 2391
7          drug  C-statistic: 0.621903, N = 8757, Events = 2285
        version                                       metric
1          base C-statistic: 0.633929, N = 23956, Events = 6992
2  short_period C-statistic: 0.634536, N = 23956, Events = 6992
3     mean_type C-statistic: 0.637365, N = 23956, Events = 6992
4 high_features C-statistic: 0.643603, N = 12524, Events = 3774
5  low_features C-statistic: 0.647774, N = 31353, Events = 8762
6         nurse C-statistic: 0.661803, N = 15826, Events = 4147
7          drug  C-statistic: 0.604782, N = 8048, Events = 3276
        version                                        metric
1          base C-statistic: 0.601325, N = 24595, Events = 24211
2  short_period C-statistic: 0.601539, N = 24595, Events = 24211
3     mean_type C-statistic: 0.602140, N = 24595, Events = 24211
4 high_features C-statistic: 0.597071, N = 16011, Events = 15731
5  low_features C-statistic: 0.606835, N = 27164, Events = 26748
6         nurse C-statistic: 0.626369, N = 19581, Events = 19217
7          drug   C-statistic: 0.537774, N = 8357, Events = 8001
        version                                      metric
1          base  C-statistic: 0.680156, N = 9163, Events = 320
2  short_period  C-statistic: 0.683105, N = 9163, Events = 320
3     mean_type  C-statistic: 0.691583, N = 9163, Events = 320
4 high_features  C-statistic: 0.646713, N = 5232, Events = 240
5  low_features C-statistic: 0.704120, N = 10340, Events = 349
6         nurse  C-statistic: 0.692551, N = 8238, Events = 304
7          drug  C-statistic: 0.666704, N = 3113, Events = 292
        version                                       metric
1          base  C-statistic: 0.713761, N = 9163, Events = 1111
2  short_period  C-statistic: 0.716254, N = 9163, Events = 1111
3     mean_type  C-statistic: 0.717802, N = 9163, Events = 1111
4 high_features   C-statistic: 0.704225, N = 5232, Events = 747
5  low_features C-statistic: 0.726151, N = 10340, Events = 1210
6         nurse  C-statistic: 0.726253, N = 8238, Events = 1026
7          drug   C-statistic: 0.654980, N = 3113, Events = 781
        version                                      metric
1          base C-statistic: 0.656943, N = 8727, Events = 2487
2  short_period C-statistic: 0.657715, N = 8727, Events = 2487
3     mean_type C-statistic: 0.658607, N = 8727, Events = 2487
4 high_features C-statistic: 0.671222, N = 4750, Events = 1389
5  low_features C-statistic: 0.668456, N = 8907, Events = 2480
6         nurse C-statistic: 0.675859, N = 6805, Events = 1811
7          drug C-statistic: 0.624572, N = 2794, Events = 1164
```

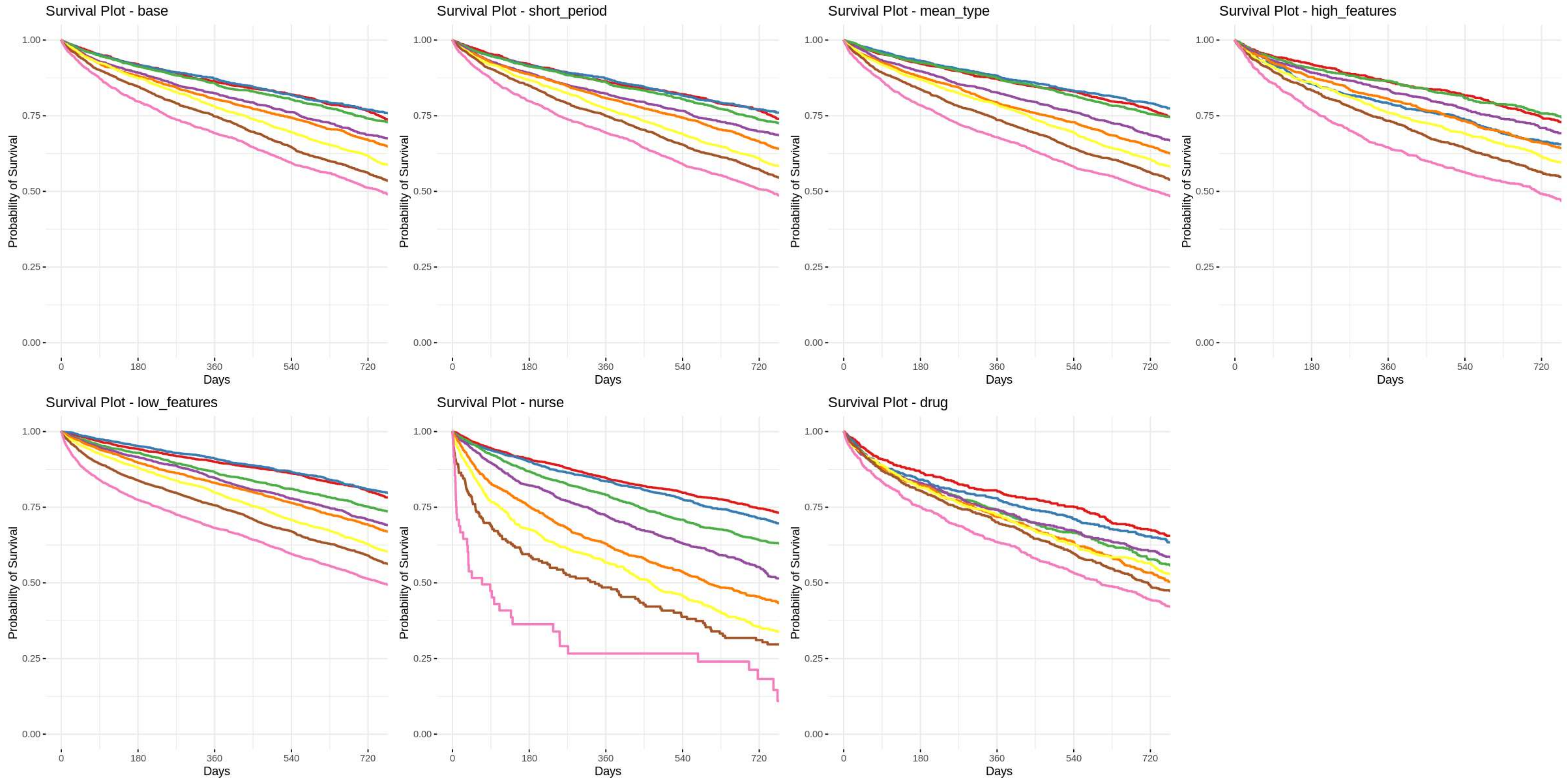

In [40]:

```r
# Usage:
# First run your comparison function
results <- outputs2
# Then create plots for each outcome
outcomes <- c("los", "ip_death", "mort_90", "mort_all")

# Generate all survival plots
survival_plots <- map(outcomes, ~create_survival_plots(results, .))
names(survival_plots) <- outcomes

# Generate all coefficient plots
coefficient_plots <- map(outcomes, ~create_coefficient_plot(results, .))
names(coefficient_plots) <- outcomes

# Optional: Create a summary metrics table
create_metrics_table <- function(results) {
  imap_dfr(results, function(result, version) {
    map_dfr(result$models, function(model) {
      tibble(
        concordance = summary(model)$concordance,
        AIC = AIC(model),
        n_events = model$nevent,
        n_total = model$n
      )
    }, .id = "outcome") %>%
      mutate(version = version)
  })
}
```

```
metrics_table <- create_metrics_table(results)
```

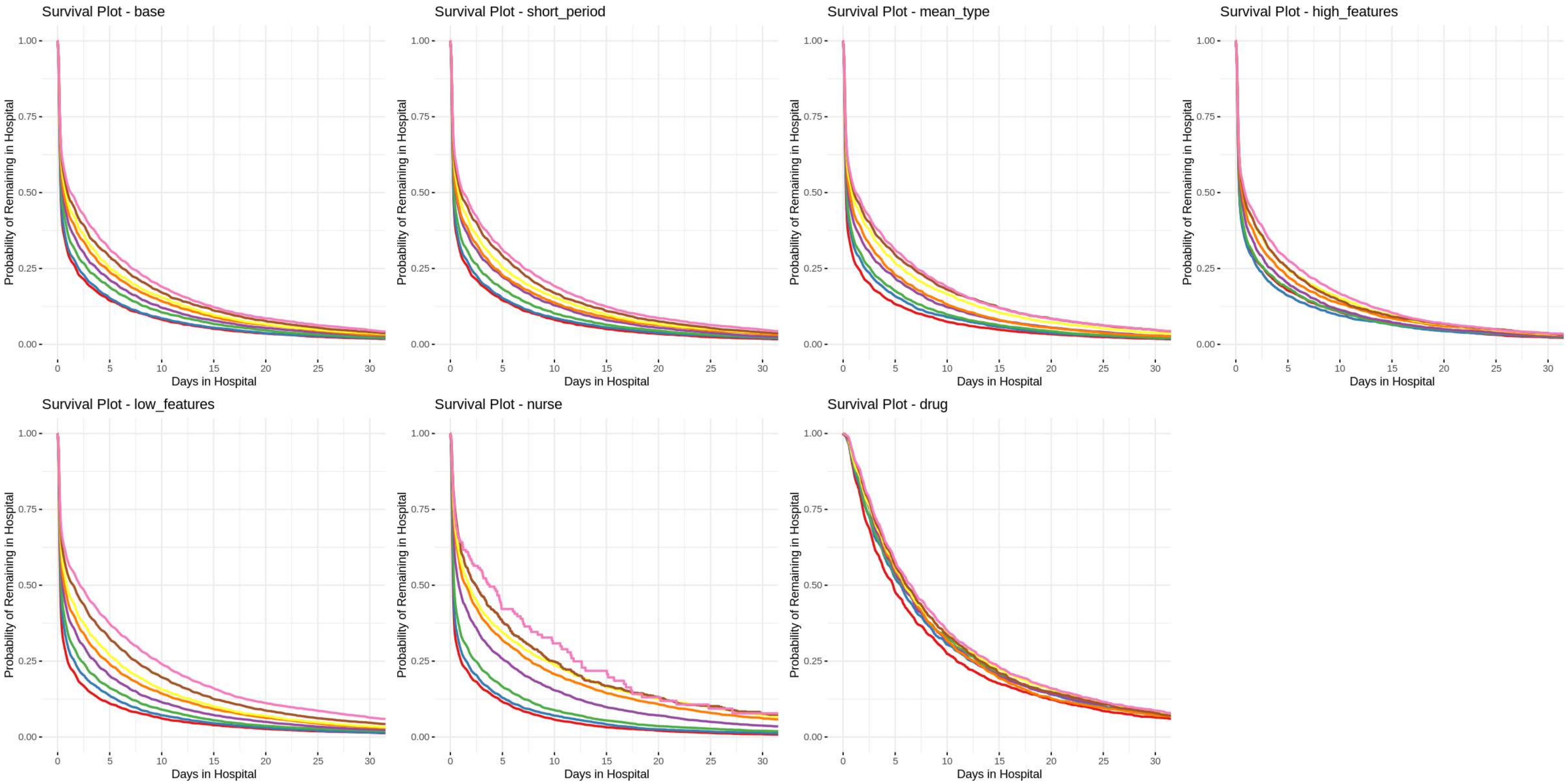

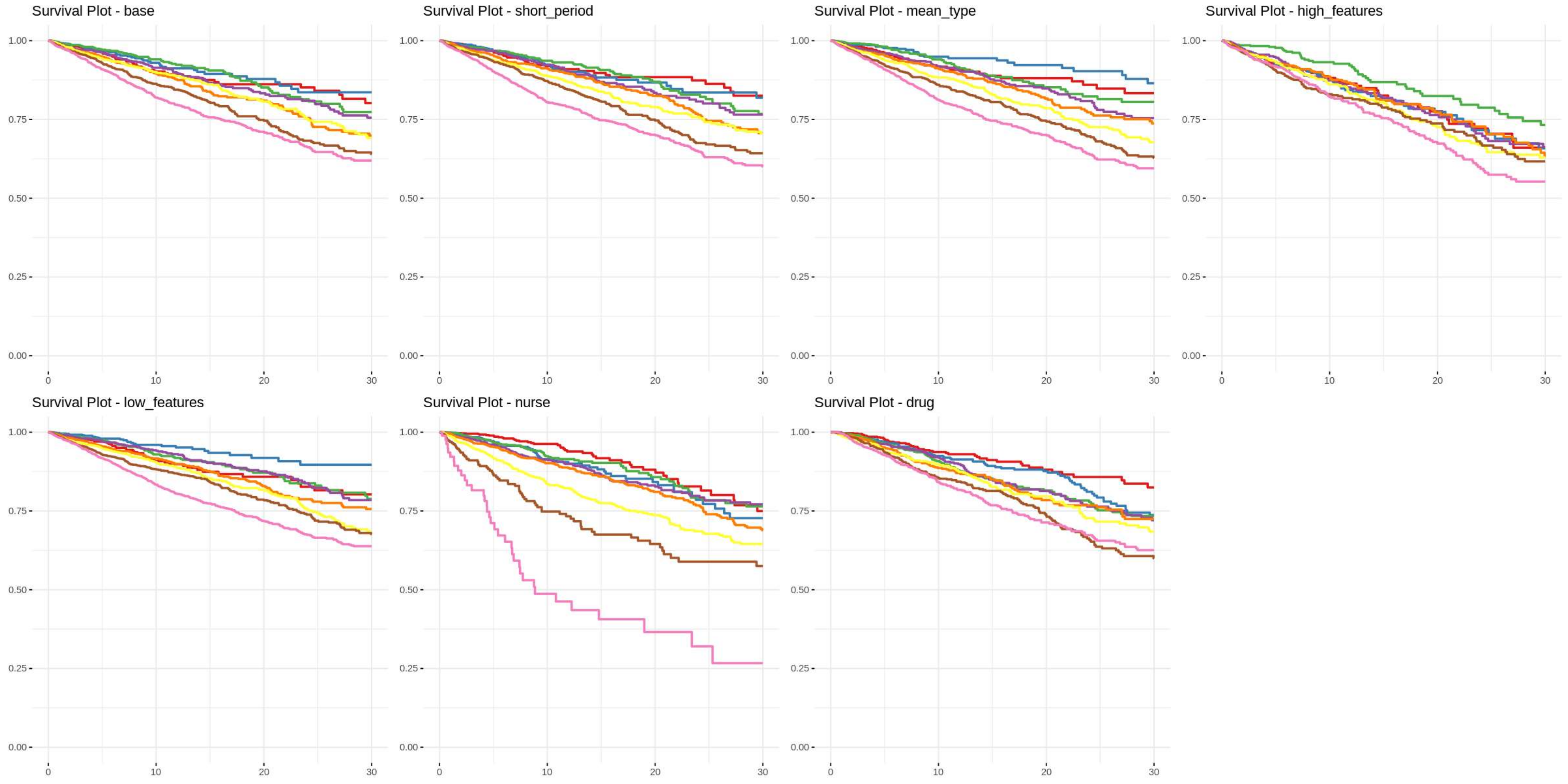
Survival Plot - base
Survival Plot - short_period
Survival Plot - mean_type
Survival Plot - high_features
Survival Plot - low_features
Survival Plot - nurse
Survival Plot - drug
1.00
0.75
0.50
0.25
0.00
0
10
20
30

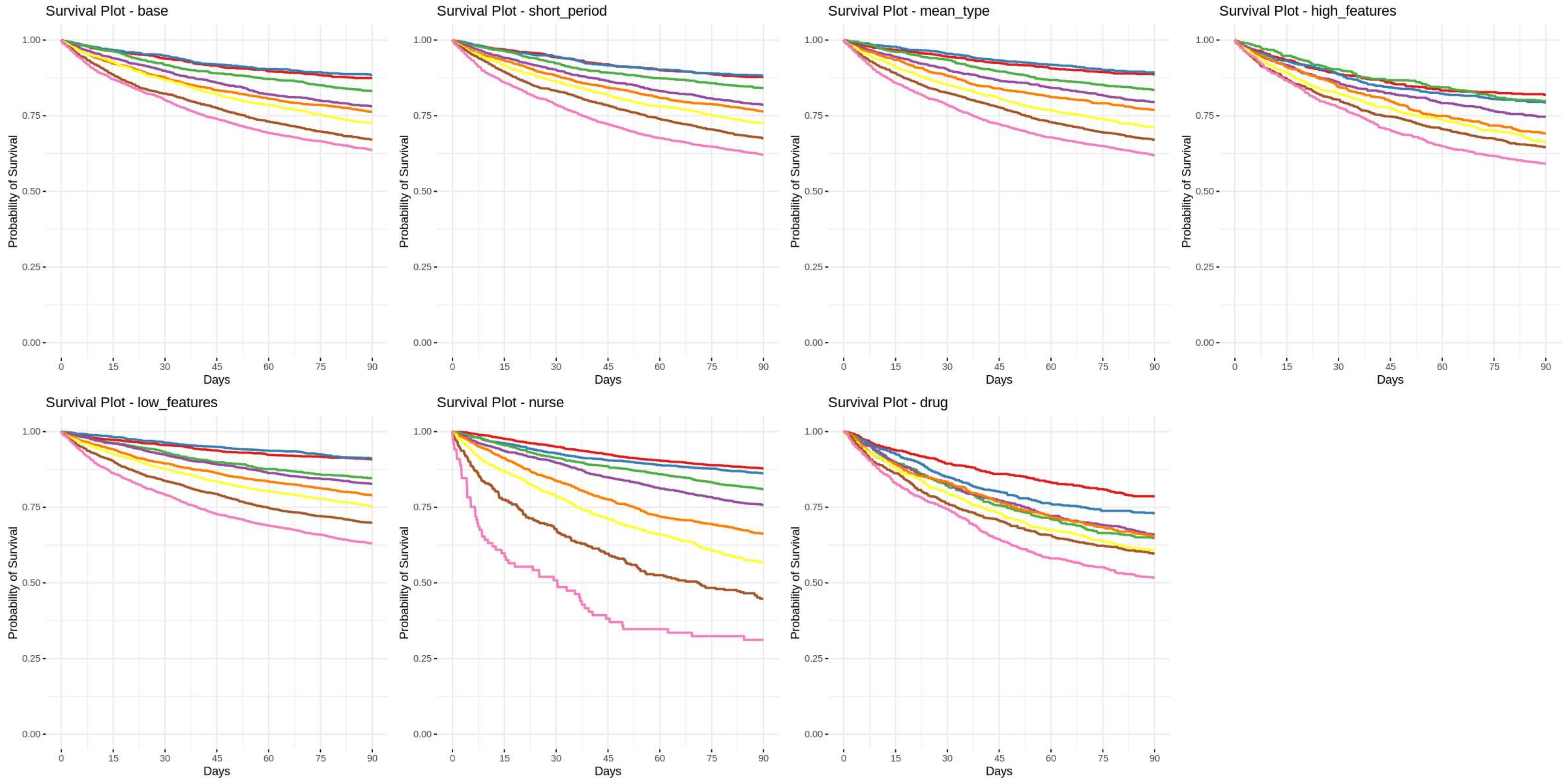

Survival Plot - base
Survival Plot - short_period
Survival Plot - mean_type
Survival Plot - high_features
Survival Plot - low_features
Survival Plot - nurse
Survival Plot - drug
Probability of Survival
Days
1.00
0.75
0.50
0.25
0.00
0
15
30
45
60
75
90

```
        version                                           metric
1          base C-statistic: 0.568753, N = 60381, Events = 59055
2  short_period C-statistic: 0.569558, N = 60381, Events = 59055
3     mean_type C-statistic: 0.571247, N = 60381, Events = 59055
4 high_features C-statistic: 0.559086, N = 36267, Events = 35359
5  low_features C-statistic: 0.588891, N = 74493, Events = 72921
6         nurse C-statistic: 0.603567, N = 39748, Events = 38897
7          drug C-statistic: 0.534056, N = 22146, Events = 21056
        version                                          metric
1          base C-statistic: 0.639390, N = 23956, Events = 1176
2  short_period C-statistic: 0.644756, N = 23956, Events = 1176
3     mean_type C-statistic: 0.651073, N = 23956, Events = 1176
4 high_features  C-statistic: 0.620535, N = 12524, Events = 808
5  low_features C-statistic: 0.646420, N = 31353, Events = 1394
6         nurse  C-statistic: 0.681738, N = 18420, Events = 742
7          drug   C-statistic: 0.654992, N = 8757, Events = 944
        version                                          metric
1          base C-statistic: 0.647744, N = 23956, Events = 3392
2  short_period C-statistic: 0.651602, N = 23956, Events = 3392
3     mean_type C-statistic: 0.658382, N = 23956, Events = 3392
4 high_features C-statistic: 0.617053, N = 12524, Events = 2085
5  low_features C-statistic: 0.671458, N = 31353, Events = 4009
6         nurse C-statistic: 0.679577, N = 18420, Events = 2391
7          drug  C-statistic: 0.621903, N = 8757, Events = 2285
        version                                          metric
1          base C-statistic: 0.633929, N = 23956, Events = 6992
2  short_period C-statistic: 0.634536, N = 23956, Events = 6992
3     mean_type C-statistic: 0.637365, N = 23956, Events = 6992
4 high_features C-statistic: 0.643603, N = 12524, Events = 3774
5  low_features C-statistic: 0.647774, N = 31353, Events = 8762
6         nurse C-statistic: 0.661803, N = 15826, Events = 4147
7          drug  C-statistic: 0.604782, N = 8048, Events = 3276
```

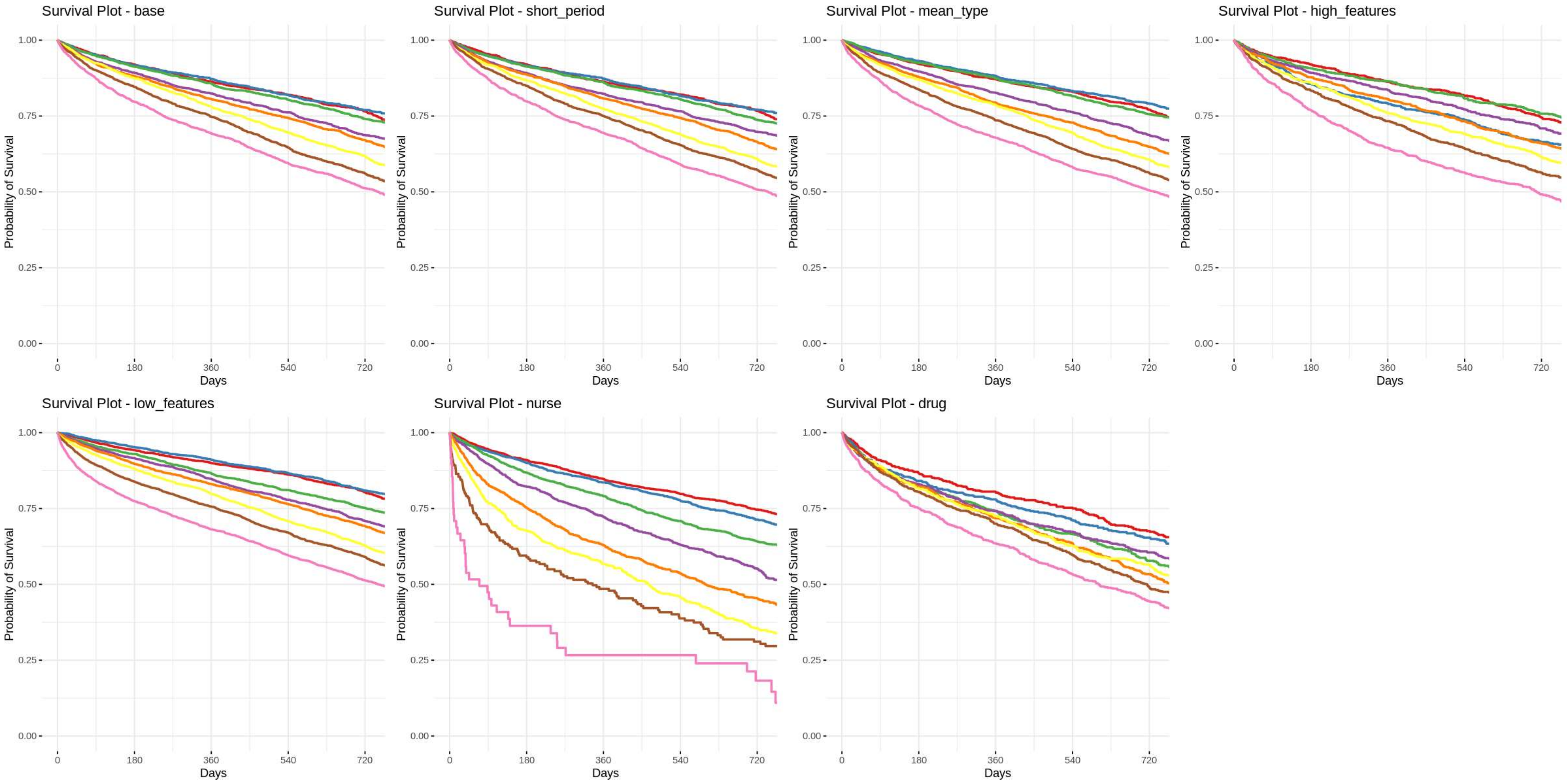

In [137…

```
coefficient_plots
```

```
Warning message:
“Removed 7 rows containing missing values (`geom_point()`).”
Warning message:
“Removed 7 rows containing missing values (`geom_errorbarh()`).”
```

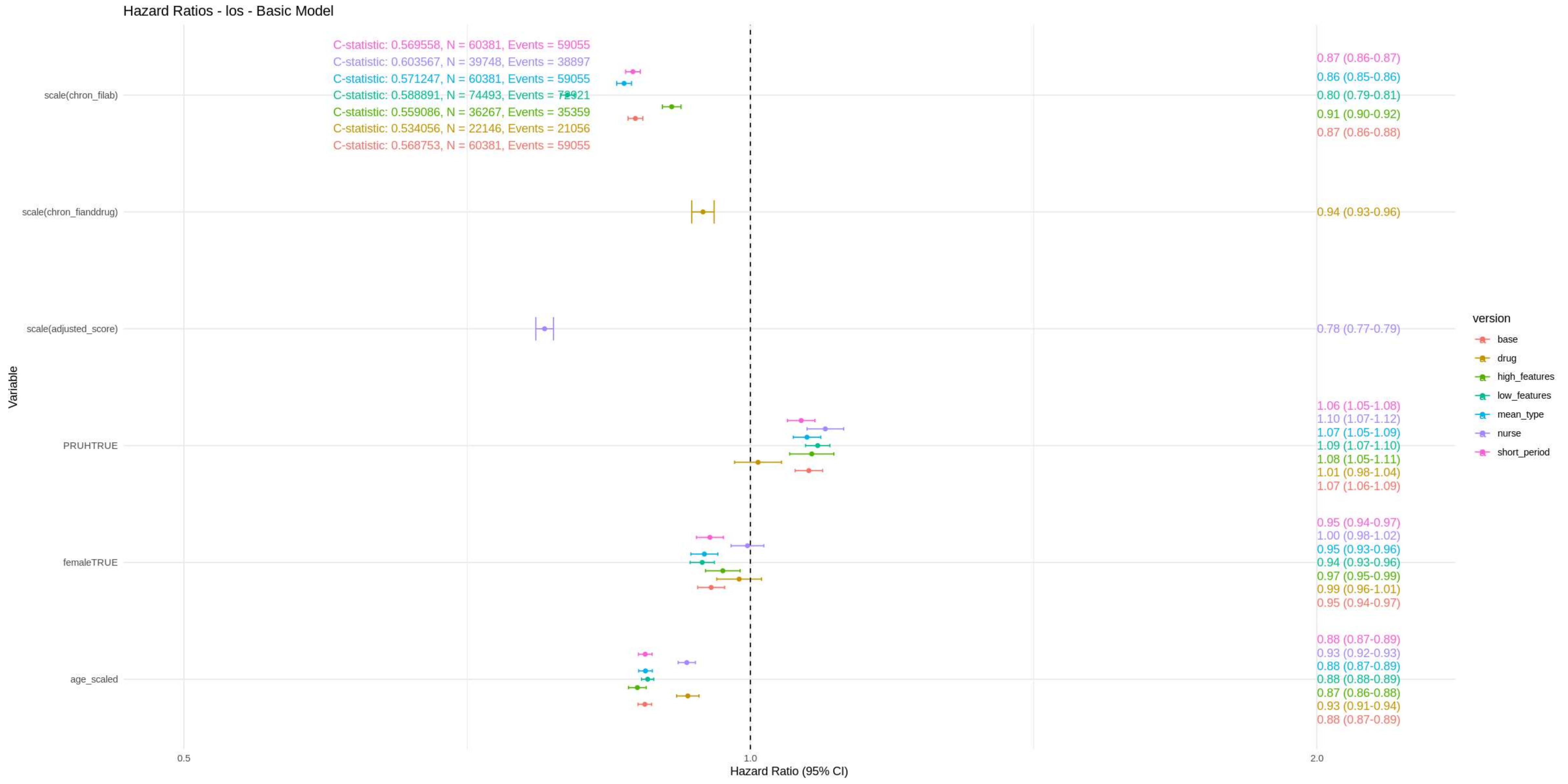
Hazard Ratios - los - Basic Model
C-statistic: 0.569558, N = 60381, Events = 59055
C-statistic: 0.603567, N = 39748, Events = 38897
C-statistic: 0.571247, N = 60381, Events = 59055
C-statistic: 0.588891, N = 74493, Events = 72921
C-statistic: 0.559086, N = 36267, Events = 35359
C-statistic: 0.534056, N = 22146, Events = 21056
C-statistic: 0.568753, N = 60381, Events = 59055
Variable
scale(chron_filab)
scale(chron_fianddrug)
scale(adjusted_score)
PRUHTRUE
femaleTRUE
age_scaled
0.87 (0.86-0.87)
0.86 (0.85-0.86)
0.80 (0.79-0.81)
0.91 (0.90-0.92)
0.87 (0.86-0.88)
0.94 (0.93-0.96)
0.78 (0.77-0.79)
1.06 (1.05-1.08)
1.10 (1.07-1.12)
1.07 (1.05-1.09)
1.09 (1.07-1.10)
1.08 (1.05-1.11)
1.01 (0.98-1.04)
1.07 (1.06-1.09)
0.95 (0.94-0.97)
1.00 (0.98-1.02)
0.95 (0.93-0.96)
0.94 (0.93-0.96)
0.97 (0.95-0.99)
0.99 (0.96-1.01)
0.95 (0.94-0.97)
0.88 (0.87-0.89)
0.93 (0.92-0.93)
0.88 (0.87-0.89)
0.88 (0.88-0.89)
0.87 (0.86-0.88)
0.93 (0.91-0.94)
0.88 (0.87-0.89)
0.5
1.0
2.0
Hazard Ratio (95% CI)
version
base
drug
high_features
low_features
mean_type
nurse
short_period

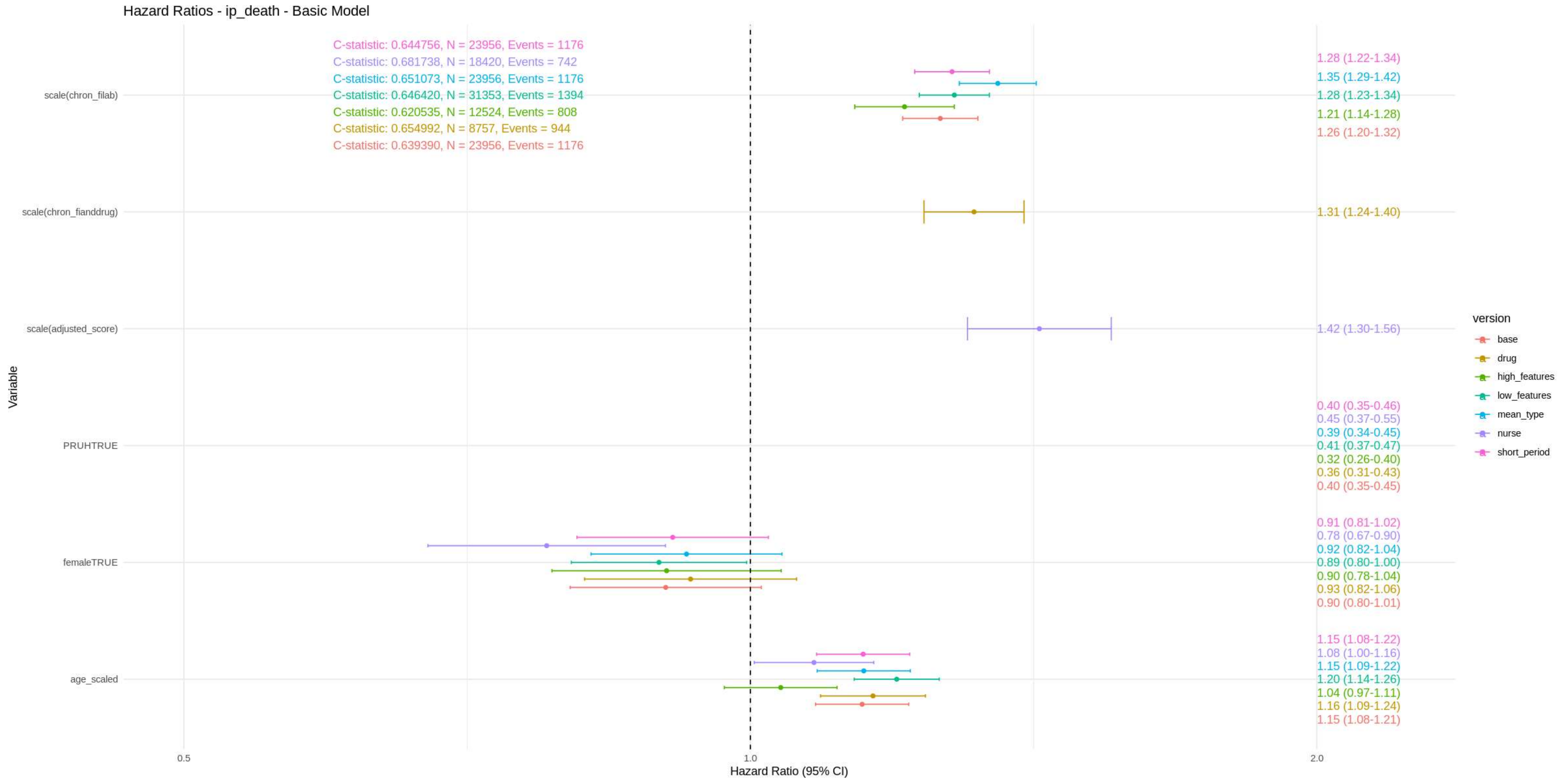

$los

$ip_death

$mort_90

$mort_all

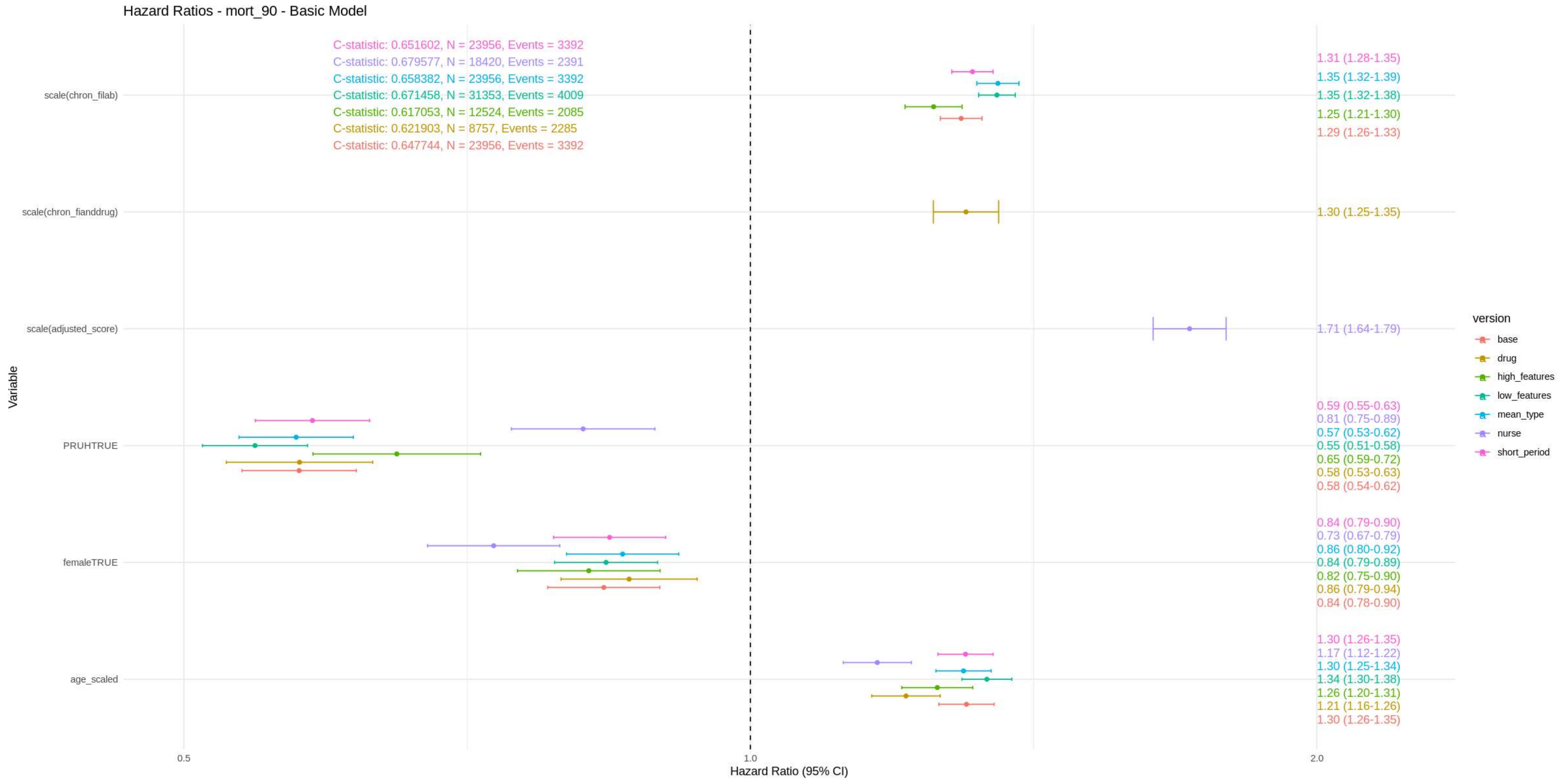

Hazard Ratios - mort_90 - Basic Model
C-statistic: 0.651602, N = 23956, Events = 3392
C-statistic: 0.679577, N = 18420, Events = 2391
C-statistic: 0.658382, N = 23956, Events = 3392
C-statistic: 0.671458, N = 31353, Events = 4009
C-statistic: 0.617053, N = 12524, Events = 2085
C-statistic: 0.621903, N = 8757, Events = 2285
C-statistic: 0.647744, N = 23956, Events = 3392
scale(chron_filab)
scale(chron_fianddrug)
scale(adjusted_score)
PRUHTRUE
femaleTRUE
age_scaled
Variable
1.31 (1.28-1.35)
1.35 (1.32-1.39)
1.35 (1.32-1.38)
1.25 (1.21-1.30)
1.29 (1.26-1.33)
1.30 (1.25-1.35)
1.71 (1.64-1.79)
0.59 (0.55-0.63)
0.81 (0.75-0.89)
0.57 (0.53-0.62)
0.55 (0.51-0.58)
0.65 (0.59-0.72)
0.58 (0.53-0.63)
0.58 (0.54-0.62)
0.84 (0.79-0.90)
0.73 (0.67-0.79)
0.86 (0.80-0.92)
0.84 (0.79-0.89)
0.82 (0.75-0.90)
0.86 (0.79-0.94)
0.84 (0.78-0.90)
1.30 (1.26-1.35)
1.17 (1.12-1.22)
1.30 (1.25-1.34)
1.34 (1.30-1.38)
1.26 (1.20-1.31)
1.21 (1.16-1.26)
1.30 (1.26-1.35)
0.5
1.0
2.0
Hazard Ratio (95% CI)
version
base
drug
high_features
low_features
mean_type
nurse
short_period

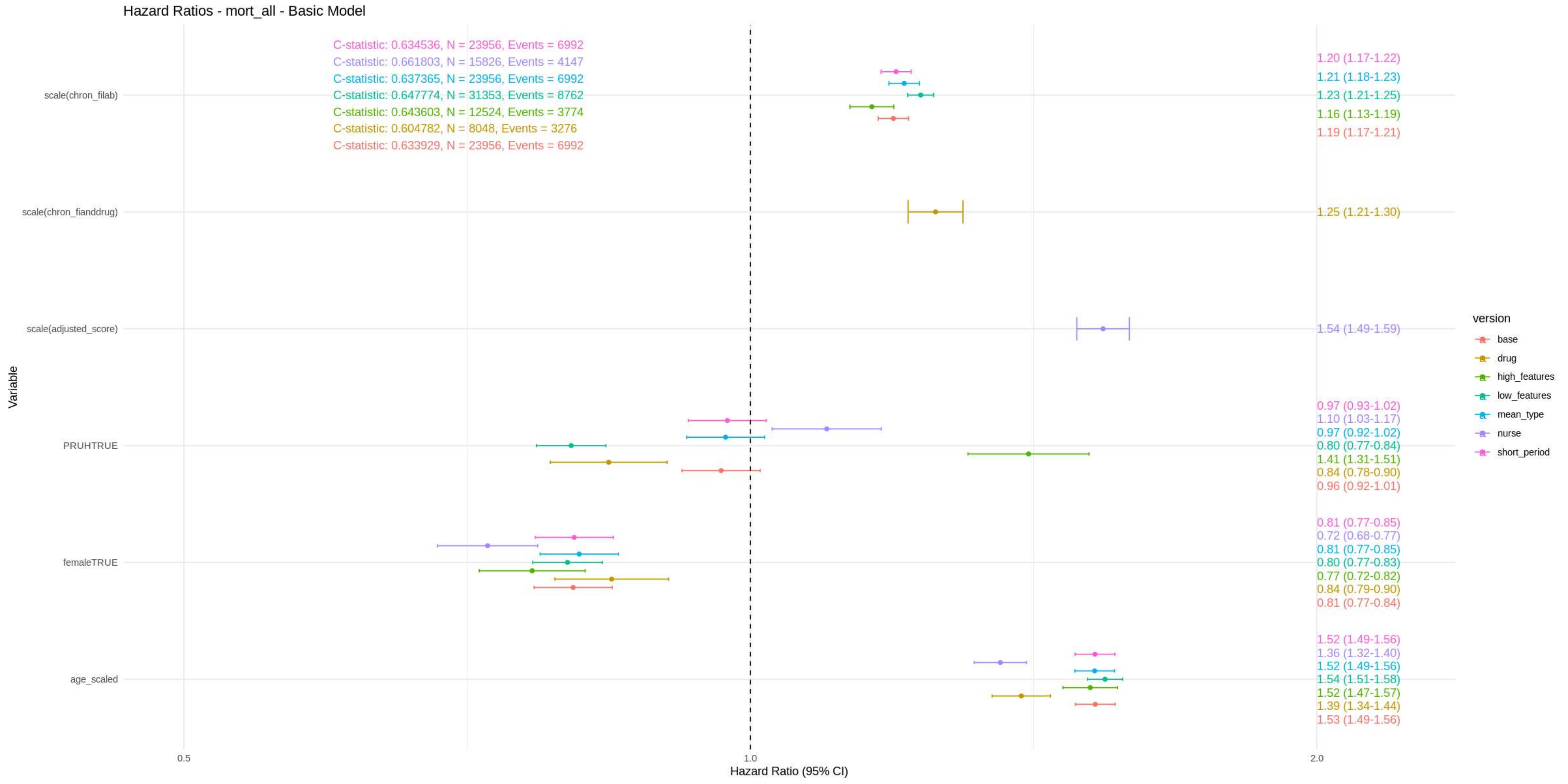

In [42]:

```
detailed_metrics <- imap_dfr(results, function(result, version) {
  map_dfr(c("basic_models", "complex_models"), function(model_type) {
    map_dfr(result[[model_type]], function(model) {
      concordance <- model$concordance
      c_stat <- concordance["concordant"] / (concordance["concordant"] + concordance["discordant"])
      coef_data <- broom::tidy(model, conf.int = TRUE)

      main_coef <- coef_data %>%
        filter(str_detect(term, "scale\\(chron_filab\\)|scale\\(adjusted_score\\)")) %>%
        slice(1)

      tibble(
        version = version,
        model_type = model_type,
        outcome = deparse(model$formula[[2]]),
        n_total = model$n,
        n_events = model$nevent,
        c_statistic = c_stat,
        AIC = AIC(model),
        coef = main_coef$estimate,
        hr = exp(main_coef$estimate),
        hr_lower = exp(main_coef$conf.low),
        hr_upper = exp(main_coef$conf.high),
        p_value = main_coef$p.value
      )
    })
  })
})
```

```r
detailed_metrics %>%
  mutate(
    hr_ci = sprintf("%.2f (%.2f-%.2f)", hr, hr_lower, hr_upper),
    c_stat = sprintf("%.3f", c_statistic)
  ) %>%
  select(version, model_type, outcome, n_total, n_events, c_stat, hr_ci, p_value) %>% write_csv("detailed_metrics.csv")
```

In [ ]:

In [41]:
```r
# Detailed metrics for ALL covariates

detailed_metrics_all_covariates <- imap_dfr(results, function(result, version) {
  # Iterate through model types ('basic_models', 'complex_models')
  map_dfr(c("basic_models", "complex_models"), function(model_type) {
    # Check if the model type exists for the current version
    if (!is.null(result[[model_type]])) {
      # Iterate through outcomes ('los', 'ip_death', 'mort_90', 'mort_all')
      map_dfr(result[[model_type]], function(model) {
        # Get concordance/c-statistic
        concordance_obj <- summary(model)$concordance
        # Handle potential NULL concordance
        if (is.null(concordance_obj)) {
            c_stat <- NA_real_
        } else if (is.numeric(concordance_obj)) {
             # Handle cases where concordance is a single number (e.g., from coxph.null) - might need adjustment based on exact structure
             c_stat <- concordance_obj[1] # Or appropriate index
        } else {
             c_stat <- concordance_obj["concordant"] / (concordance_obj["concordant"] + concordance_obj["discordant"])
             # Handle cases where concordance exists but might lack 'concordant'/'discordant' names properly
             if (is.nan(c_stat) || is.null(c_stat)) { c_stat <- NA_real_ }
        }

        # Use broom::tidy to get coefficients for ALL terms
        tidy_model <- broom::tidy(model, conf.int = TRUE, exponentiate = TRUE) # Exponentiate directly gives HR

        # Add metadata and return for this model
        tidy_model %>%
          mutate(
            version = version,
            model_type = model_type,
            outcome = deparse(model$formula[[2]]), # Extracts the Surv() part
            n_total = model$n,
            n_events = model$nevent,
            c_statistic = c_stat,
            AIC = AIC(model),
            # Rename exponentiated columns for clarity
            hr = estimate,
            hr_lower = conf.low,
            hr_upper = conf.high
          ) %>%
          # Select and arrange final columns
          select(
            version, model_type, outcome, term,
            hr, hr_lower, hr_upper, p.value,
            c_statistic, AIC, n_total, n_events
          )

      }, .id = "outcome_name") # Pass outcome name if needed, though already extracted
    } else {
      # Return empty tibble if model type doesn't exist (e.g., complex model for drug adj)
      tibble()
    }
  })
})
```

```r
# Optional: Format the HR and C-stat for printing/display
detailed_metrics_all_covariates_formatted <- detailed_metrics_all_covariates %>%
  mutate(
    hr_ci = sprintf("%.2f (%.2f-%.2f)", hr, hr_lower, hr_upper),
    c_stat_formatted = sprintf("%.3f", c_statistic),
    p_value_formatted = format.pval(p.value, eps = 0.001, digits = 3) # Format p-value nicely
  ) %>%
  select(
    version, model_type, outcome, term, hr_ci, p_value_formatted, c_stat_formatted, AIC, n_total, n_events
  )



# Save to CSV
write.csv(detailed_metrics_all_covariates, "detailed_metrics_all_covariates.csv", row.names = FALSE)
```

In [144…

```r
# If needed, install packages:
# install.packages("knitr")
# install.packages("kableExtra")

library(knitr)

kable(detailed_metrics_all_covariates_formatted)
```

version	model_type	outcome	term	hr_ci	p_value_formatted	c_stat_formatted	AIC	n_total	n_events
base	basic_models	Surv(los, !died_ip)	scale(chron_filab)	0.87 (0.86-0.88)	< 0.001	0.569	1183015.981	60381	59055
base	basic_models	Surv(los, !died_ip)	age_scaled	0.88 (0.87-0.89)	< 0.001	0.569	1183015.981	60381	59055
base	basic_models	Surv(los, !died_ip)	femaleTRUE	0.95 (0.94-0.97)	< 0.001	0.569	1183015.981	60381	59055
base	basic_models	Surv(los, !died_ip)	PRUHTRUE	1.07 (1.06-1.09)	< 0.001	0.569	1183015.981	60381	59055
base	basic_models	Surv(los, died_ip)	scale(chron_filab)	1.26 (1.20-1.32)	< 0.001	0.639	19467.003	23956	1176
base	basic_models	Surv(los, died_ip)	age_scaled	1.15 (1.08-1.21)	< 0.001	0.639	19467.003	23956	1176
base	basic_models	Surv(los, died_ip)	femaleTRUE	0.90 (0.80-1.01)	0.08257	0.639	19467.003	23956	1176
base	basic_models	Surv(los, died_ip)	PRUHTRUE	0.40 (0.35-0.45)	< 0.001	0.639	19467.003	23956	1176
base	basic_models	Surv(days_censored, status_censored)	scale(chron_filab)	1.29 (1.26-1.33)	< 0.001	0.648	63192.208	23956	3392
base	basic_models	Surv(days_censored, status_censored)	age_scaled	1.30 (1.26-1.35)	< 0.001	0.648	63192.208	23956	3392
base	basic_models	Surv(days_censored, status_censored)	femaleTRUE	0.84 (0.78-0.90)	< 0.001	0.648	63192.208	23956	3392
base	basic_models	Surv(days_censored, status_censored)	PRUHTRUE	0.58 (0.54-0.62)	< 0.001	0.648	63192.208	23956	3392
base	basic_models	Surv(days_elapsed, !Alive)	scale(chron_filab)	1.19 (1.17-1.21)	< 0.001	0.634	126992.302	23956	6992
base	basic_models	Surv(days_elapsed, !Alive)	age_scaled	1.53 (1.49-1.56)	< 0.001	0.634	126992.302	23956	6992
base	basic_models	Surv(days_elapsed, !Alive)	femaleTRUE	0.81 (0.77-0.84)	< 0.001	0.634	126992.302	23956	6992
base	basic_models	Surv(days_elapsed, !Alive)	PRUHTRUE	0.96 (0.92-1.01)	0.14224	0.634	126992.302	23956	6992
base	complex_models	Surv(los, !died_ip)	scale(chron_filab)	0.90 (0.89-0.92)	< 0.001	0.601	440434.044	24595	24211
base	complex_models	Surv(los, !died_ip)	age_scaled	0.86 (0.85-0.87)	< 0.001	0.601	440434.044	24595	24211
base	complex_models	Surv(los, !died_ip)	femaleTRUE	0.95 (0.92-0.97)	< 0.001	0.601	440434.044	24595	24211
base	complex_models	Surv(los, !died_ip)	PRUHTRUE	1.27 (1.22-1.31)	< 0.001	0.601	440434.044	24595	24211
base	complex_models	Surv(los, !died_ip)	news_scaled	0.87 (0.86-0.88)	< 0.001	0.601	440434.044	24595	24211
base	complex_models	Surv(los, !died_ip)	imd_scaled	1.03 (1.01-1.04)	0.00165	0.601	440434.044	24595	24211
base	complex_models	Surv(los, !died_ip)	fi_acute_scaled	0.91 (0.90-0.93)	< 0.001	0.601	440434.044	24595	24211
base	complex_models	Surv(los, died_ip)	scale(chron_filab)	1.13 (0.99-1.28)	0.06021	0.680	4456.316	9163	320
base	complex_models	Surv(los, died_ip)	age_scaled	1.13 (1.02-1.26)	0.02467	0.680	4456.316	9163	320
base	complex_models	Surv(los, died_ip)	femaleTRUE	0.95 (0.76-1.19)	0.64209	0.680	4456.316	9163	320
base	complex_models	Surv(los, died_ip)	PRUHTRUE	0.39 (0.26-0.59)	< 0.001	0.680	4456.316	9163	320
base	complex_models	Surv(los, died_ip)	news_scaled	1.32 (1.23-1.42)	< 0.001	0.680	4456.316	9163	320
base	complex_models	Surv(los, died_ip)	imd_scaled	0.94 (0.81-1.09)	0.43786	0.680	4456.316	9163	320
base	complex_models	Surv(los, died_ip)	fi_acute_scaled	1.25 (1.11-1.41)	< 0.001	0.680	4456.316	9163	320
base	complex_models	Surv(days_censored, status_censored)	scale(chron_filab)	1.24 (1.17-1.31)	< 0.001	0.714	18083.836	9163	1111
base	complex_models	Surv(days_censored, status_censored)	age_scaled	1.32 (1.24-1.40)	< 0.001	0.714	18083.836	9163	1111
base	complex_models	Surv(days_censored, status_censored)	femaleTRUE	0.89 (0.79-1.01)	0.06415	0.714	18083.836	9163	1111
base	complex_models	Surv(days_censored, status_censored)	PRUHTRUE	0.52 (0.44-0.62)	< 0.001	0.714	18083.836	9163	1111
base	complex_models	Surv(days_censored, status_censored)	news_scaled	1.38 (1.33-1.44)	< 0.001	0.714	18083.836	9163	1111
base	complex_models	Surv(days_censored, status_censored)	imd_scaled	0.93 (0.86-1.01)	0.07133	0.714	18083.836	9163	1111
base	complex_models	Surv(days_censored, status_censored)	fi_acute_scaled	1.31 (1.23-1.39)	< 0.001	0.714	18083.836	9163	1111
base	complex_models	Surv(days_elapsed, !Alive)	scale(chron_filab)	1.18 (1.14-1.23)	< 0.001	0.657	39755.395	8727	2487
base	complex_models	Surv(days_elapsed, !Alive)	age_scaled	1.46 (1.40-1.52)	< 0.001	0.657	39755.395	8727	2487
base	complex_models	Surv(days_elapsed, !Alive)	femaleTRUE	0.83 (0.76-0.90)	< 0.001	0.657	39755.395	8727	2487
base	complex_models	Surv(days_elapsed, !Alive)	PRUHTRUE	0.88 (0.79-0.98)	0.02045	0.657	39755.395	8727	2487
base	complex_models	Surv(days_elapsed, !Alive)	news_scaled	1.21 (1.17-1.25)	< 0.001	0.657	39755.395	8727	2487
base	complex_models	Surv(days_elapsed, !Alive)	imd_scaled	0.95 (0.90-1.00)	0.03585	0.657	39755.395	8727	2487
base	complex_models	Surv(days_elapsed, !Alive)	fi_acute_scaled	1.18 (1.14-1.24)	< 0.001	0.657	39755.395	8727	2487
short_period	basic_models	Surv(los, !died_ip)	scale(chron_filab)	0.87 (0.86-0.87)	< 0.001	0.570	1182966.300	60381	59055
short_period	basic_models	Surv(los, !died_ip)	age_scaled	0.88 (0.87-0.89)	< 0.001	0.570	1182966.300	60381	59055
short_period	basic_models	Surv(los, !died_ip)	femaleTRUE	0.95 (0.94-0.97)	< 0.001	0.570	1182966.300	60381	59055
short_period	basic_models	Surv(los, !died_ip)	PRUHTRUE	1.06 (1.05-1.08)	< 0.001	0.570	1182966.300	60381	59055
short_period	basic_models	Surv(los, died_ip)	scale(chron_filab)	1.28 (1.22-1.34)	< 0.001	0.645	19455.284	23956	1176
short_period	basic_models	Surv(los, died_ip)	age_scaled	1.15 (1.08-1.22)	< 0.001	0.645	19455.284	23956	1176
short_period	basic_models	Surv(los, died_ip)	femaleTRUE	0.91 (0.81-1.02)	0.11161	0.645	19455.284	23956	1176
short_period	basic_models	Surv(los, died_ip)	PRUHTRUE	0.40 (0.35-0.46)	< 0.001	0.645	19455.284	23956	1176
short_period	basic_models	Surv(days_censored, status_censored)	scale(chron_filab)	1.31 (1.28-1.35)	< 0.001	0.652	63153.302	23956	3392
short_period	basic_models	Surv(days_censored, status_censored)	age_scaled	1.30 (1.26-1.35)	< 0.001	0.652	63153.302	23956	3392
short_period	basic_models	Surv(days_censored, status_censored)	femaleTRUE	0.84 (0.79-0.90)	< 0.001	0.652	63153.302	23956	3392
short_period	basic_models	Surv(days_censored, status_censored)	PRUHTRUE	0.59 (0.55-0.63)	< 0.001	0.652	63153.302	23956	3392
short_period	basic_models	Surv(days_elapsed, !Alive)	scale(chron_filab)	1.20 (1.17-1.22)	< 0.001	0.635	126977.280	23956	6992
short_period	basic_models	Surv(days_elapsed, !Alive)	age_scaled	1.52 (1.49-1.56)	< 0.001	0.635	126977.280	23956	6992
short_period	basic_models	Surv(days_elapsed, !Alive)	femaleTRUE	0.81 (0.77-0.85)	< 0.001	0.635	126977.280	23956	6992
short_period	basic_models	Surv(days_elapsed, !Alive)	PRUHTRUE	0.97 (0.93-1.02)	0.24718	0.635	126977.280	23956	6992
short_period	complex_models	Surv(los, !died_ip)	scale(chron_filab)	0.90 (0.89-0.92)	< 0.001	0.602	440427.576	24595	24211
short_period	complex_models	Surv(los, !died_ip)	age_scaled	0.86 (0.85-0.88)	< 0.001	0.602	440427.576	24595	24211
short_period	complex_models	Surv(los, !died_ip)	femaleTRUE	0.95 (0.92-0.97)	< 0.001	0.602	440427.576	24595	24211

short_period	complex_models	Surv(los, !died_ip)	PRUHTRUE	1.26 (1.21-1.31)	< 0.001	0.602	440427.576	24595	24211
short_period	complex_models	Surv(los, !died_ip)	news_scaled	0.87 (0.86-0.88)	< 0.001	0.602	440427.576	24595	24211
short_period	complex_models	Surv(los, !died_ip)	imd_scaled	1.03 (1.01-1.04)	0.00147	0.602	440427.576	24595	24211
short_period	complex_models	Surv(los, !died_ip)	fi_acute_scaled	0.91 (0.90-0.93)	< 0.001	0.602	440427.576	24595	24211
short_period	complex_models	Surv(los, died_ip)	scale(chron_filab)	1.14 (1.01-1.30)	0.04013	0.683	4455.648	9163	320
short_period	complex_models	Surv(los, died_ip)	age_scaled	1.13 (1.02-1.26)	0.02533	0.683	4455.648	9163	320
short_period	complex_models	Surv(los, died_ip)	femaleTRUE	0.95 (0.76-1.19)	0.64933	0.683	4455.648	9163	320
short_period	complex_models	Surv(los, died_ip)	PRUHTRUE	0.39 (0.26-0.60)	< 0.001	0.683	4455.648	9163	320
short_period	complex_models	Surv(los, died_ip)	news_scaled	1.32 (1.23-1.42)	< 0.001	0.683	4455.648	9163	320
short_period	complex_models	Surv(los, died_ip)	imd_scaled	0.94 (0.81-1.09)	0.43764	0.683	4455.648	9163	320
short_period	complex_models	Surv(los, died_ip)	fi_acute_scaled	1.24 (1.10-1.40)	< 0.001	0.683	4455.648	9163	320
short_period	complex_models	Surv(days_censored, status_censored)	scale(chron_filab)	1.27 (1.20-1.35)	< 0.001	0.716	18070.180	9163	1111
short_period	complex_models	Surv(days_censored, status_censored)	age_scaled	1.32 (1.24-1.40)	< 0.001	0.716	18070.180	9163	1111
short_period	complex_models	Surv(days_censored, status_censored)	femaleTRUE	0.90 (0.79-1.01)	0.07606	0.716	18070.180	9163	1111
short_period	complex_models	Surv(days_censored, status_censored)	PRUHTRUE	0.53 (0.45-0.63)	< 0.001	0.716	18070.180	9163	1111
short_period	complex_models	Surv(days_censored, status_censored)	news_scaled	1.38 (1.33-1.44)	< 0.001	0.716	18070.180	9163	1111
short_period	complex_models	Surv(days_censored, status_censored)	imd_scaled	0.93 (0.86-1.01)	0.07115	0.716	18070.180	9163	1111
short_period	complex_models	Surv(days_censored, status_censored)	fi_acute_scaled	1.29 (1.22-1.37)	< 0.001	0.716	18070.180	9163	1111
short_period	complex_models	Surv(days_elapsed, !Alive)	scale(chron_filab)	1.20 (1.15-1.25)	< 0.001	0.658	39747.517	8727	2487
short_period	complex_models	Surv(days_elapsed, !Alive)	age_scaled	1.46 (1.40-1.52)	< 0.001	0.658	39747.517	8727	2487
short_period	complex_models	Surv(days_elapsed, !Alive)	femaleTRUE	0.83 (0.77-0.90)	< 0.001	0.658	39747.517	8727	2487
short_period	complex_models	Surv(days_elapsed, !Alive)	PRUHTRUE	0.89 (0.80-0.99)	0.02992	0.658	39747.517	8727	2487
short_period	complex_models	Surv(days_elapsed, !Alive)	news_scaled	1.21 (1.17-1.25)	< 0.001	0.658	39747.517	8727	2487
short_period	complex_models	Surv(days_elapsed, !Alive)	imd_scaled	0.95 (0.90-1.00)	0.03278	0.658	39747.517	8727	2487
short_period	complex_models	Surv(days_elapsed, !Alive)	fi_acute_scaled	1.18 (1.13-1.23)	< 0.001	0.658	39747.517	8727	2487
mean_type	basic_models	Surv(los, !died_ip)	scale(chron_filab)	0.86 (0.85-0.86)	< 0.001	0.571	1182831.564	60381	59055
mean_type	basic_models	Surv(los, !died_ip)	age_scaled	0.88 (0.87-0.89)	< 0.001	0.571	1182831.564	60381	59055
mean_type	basic_models	Surv(los, !died_ip)	femaleTRUE	0.95 (0.93-0.96)	< 0.001	0.571	1182831.564	60381	59055
mean_type	basic_models	Surv(los, !died_ip)	PRUHTRUE	1.07 (1.05-1.09)	< 0.001	0.571	1182831.564	60381	59055
mean_type	basic_models	Surv(los, died_ip)	scale(chron_filab)	1.35 (1.29-1.42)	< 0.001	0.651	19418.541	23956	1176
mean_type	basic_models	Surv(los, died_ip)	age_scaled	1.15 (1.09-1.22)	< 0.001	0.651	19418.541	23956	1176
mean_type	basic_models	Surv(los, died_ip)	femaleTRUE	0.92 (0.82-1.04)	0.18991	0.651	19418.541	23956	1176
mean_type	basic_models	Surv(los, died_ip)	PRUHTRUE	0.39 (0.34-0.45)	< 0.001	0.651	19418.541	23956	1176
mean_type	basic_models	Surv(days_censored, status_censored)	scale(chron_filab)	1.35 (1.32-1.39)	< 0.001	0.658	63084.188	23956	3392
mean_type	basic_models	Surv(days_censored, status_censored)	age_scaled	1.30 (1.25-1.34)	< 0.001	0.658	63084.188	23956	3392
mean_type	basic_models	Surv(days_censored, status_censored)	femaleTRUE	0.86 (0.80-0.92)	< 0.001	0.658	63084.188	23956	3392
mean_type	basic_models	Surv(days_censored, status_censored)	PRUHTRUE	0.57 (0.53-0.62)	< 0.001	0.658	63084.188	23956	3392
mean_type	basic_models	Surv(days_elapsed, !Alive)	scale(chron_filab)	1.21 (1.18-1.23)	< 0.001	0.637	126949.469	23956	6992
mean_type	basic_models	Surv(days_elapsed, !Alive)	age_scaled	1.52 (1.49-1.56)	< 0.001	0.637	126949.469	23956	6992
mean_type	basic_models	Surv(days_elapsed, !Alive)	femaleTRUE	0.81 (0.77-0.85)	< 0.001	0.637	126949.469	23956	6992
mean_type	basic_models	Surv(days_elapsed, !Alive)	PRUHTRUE	0.97 (0.92-1.02)	0.21293	0.637	126949.469	23956	6992
mean_type	complex_models	Surv(los, !died_ip)	scale(chron_filab)	0.89 (0.88-0.91)	< 0.001	0.602	440389.374	24595	24211
mean_type	complex_models	Surv(los, !died_ip)	age_scaled	0.86 (0.85-0.88)	< 0.001	0.602	440389.374	24595	24211
mean_type	complex_models	Surv(los, !died_ip)	femaleTRUE	0.94 (0.92-0.96)	< 0.001	0.602	440389.374	24595	24211
mean_type	complex_models	Surv(los, !died_ip)	PRUHTRUE	1.26 (1.22-1.31)	< 0.001	0.602	440389.374	24595	24211
mean_type	complex_models	Surv(los, !died_ip)	news_scaled	0.87 (0.86-0.88)	< 0.001	0.602	440389.374	24595	24211
mean_type	complex_models	Surv(los, !died_ip)	imd_scaled	1.03 (1.01-1.04)	0.00138	0.602	440389.374	24595	24211
mean_type	complex_models	Surv(los, !died_ip)	fi_acute_scaled	0.92 (0.90-0.93)	< 0.001	0.602	440389.374	24595	24211
mean_type	complex_models	Surv(los, died_ip)	scale(chron_filab)	1.25 (1.10-1.41)	< 0.001	0.692	4448.766	9163	320
mean_type	complex_models	Surv(los, died_ip)	age_scaled	1.13 (1.02-1.26)	0.02310	0.692	4448.766	9163	320
mean_type	complex_models	Surv(los, died_ip)	femaleTRUE	0.98 (0.78-1.23)	0.86263	0.692	4448.766	9163	320
mean_type	complex_models	Surv(los, died_ip)	PRUHTRUE	0.39 (0.25-0.59)	< 0.001	0.692	4448.766	9163	320
mean_type	complex_models	Surv(los, died_ip)	news_scaled	1.32 (1.23-1.42)	< 0.001	0.692	4448.766	9163	320
mean_type	complex_models	Surv(los, died_ip)	imd_scaled	0.94 (0.81-1.09)	0.40028	0.692	4448.766	9163	320
mean_type	complex_models	Surv(los, died_ip)	fi_acute_scaled	1.22 (1.08-1.37)	0.00132	0.692	4448.766	9163	320
mean_type	complex_models	Surv(days_censored, status_censored)	scale(chron_filab)	1.29 (1.22-1.37)	< 0.001	0.718	18065.351	9163	1111
mean_type	complex_models	Surv(days_censored, status_censored)	age_scaled	1.31 (1.24-1.39)	< 0.001	0.718	18065.351	9163	1111
mean_type	complex_models	Surv(days_censored, status_censored)	femaleTRUE	0.91 (0.81-1.03)	0.12838	0.718	18065.351	9163	1111
mean_type	complex_models	Surv(days_censored, status_censored)	PRUHTRUE	0.52 (0.44-0.62)	< 0.001	0.718	18065.351	9163	1111
mean_type	complex_models	Surv(days_censored, status_censored)	news_scaled	1.38 (1.33-1.44)	< 0.001	0.718	18065.351	9163	1111
mean_type	complex_models	Surv(days_censored, status_censored)	imd_scaled	0.93 (0.86-1.01)	0.07702	0.718	18065.351	9163	1111
mean_type	complex_models	Surv(days_censored, status_censored)	fi_acute_scaled	1.29 (1.21-1.37)	< 0.001	0.718	18065.351	9163	1111
mean_type	complex_models	Surv(days_elapsed, !Alive)	scale(chron_filab)	1.19 (1.14-1.23)	< 0.001	0.659	39753.757	8727	2487
mean_type	complex_models	Surv(days_elapsed, !Alive)	age_scaled	1.46 (1.40-1.52)	< 0.001	0.659	39753.757	8727	2487
mean_type	complex_models	Surv(days_elapsed, !Alive)	femaleTRUE	0.83 (0.77-0.90)	< 0.001	0.659	39753.757	8727	2487
mean_type	complex_models	Surv(days_elapsed, !Alive)	PRUHTRUE	0.89 (0.80-0.99)	0.03587	0.659	39753.757	8727	2487

mean_type	complex_models	Surv(days_elapsed, !Alive)	news_scaled	1.20 (1.16-1.25)	< 0.001	0.659	39753.757	8727	2487
mean_type	complex_models	Surv(days_elapsed, !Alive)	imd_scaled	0.95 (0.90-1.00)	0.04273	0.659	39753.757	8727	2487
mean_type	complex_models	Surv(days_elapsed, !Alive)	fi_acute_scaled	1.19 (1.14-1.24)	< 0.001	0.659	39753.757	8727	2487
high_features	basic_models	Surv(los, !died_ip)	scale(chron_filab)	0.91 (0.90-0.92)	< 0.001	0.559	672906.221	36267	35359
high_features	basic_models	Surv(los, !died_ip)	age_scaled	0.87 (0.86-0.88)	< 0.001	0.559	672906.221	36267	35359
high_features	basic_models	Surv(los, !died_ip)	femaleTRUE	0.97 (0.95-0.99)	0.00180	0.559	672906.221	36267	35359
high_features	basic_models	Surv(los, !died_ip)	PRUHTRUE	1.08 (1.05-1.11)	< 0.001	0.559	672906.221	36267	35359
high_features	basic_models	Surv(los, died_ip)	scale(chron_filab)	1.21 (1.14-1.28)	< 0.001	0.621	12416.935	12524	808
high_features	basic_models	Surv(los, died_ip)	age_scaled	1.04 (0.97-1.11)	0.29110	0.621	12416.935	12524	808
high_features	basic_models	Surv(los, died_ip)	femaleTRUE	0.90 (0.78-1.04)	0.15203	0.621	12416.935	12524	808
high_features	basic_models	Surv(los, died_ip)	PRUHTRUE	0.32 (0.26-0.40)	< 0.001	0.621	12416.935	12524	808
high_features	basic_models	Surv(days_censored, status_censored)	scale(chron_filab)	1.25 (1.21-1.30)	< 0.001	0.617	36124.194	12524	2085
high_features	basic_models	Surv(days_censored, status_censored)	age_scaled	1.26 (1.20-1.31)	< 0.001	0.617	36124.194	12524	2085
high_features	basic_models	Surv(days_censored, status_censored)	femaleTRUE	0.82 (0.75-0.90)	< 0.001	0.617	36124.194	12524	2085
high_features	basic_models	Surv(days_censored, status_censored)	PRUHTRUE	0.65 (0.59-0.72)	< 0.001	0.617	36124.194	12524	2085
high_features	basic_models	Surv(days_elapsed, !Alive)	scale(chron_filab)	1.16 (1.13-1.19)	< 0.001	0.644	63542.560	12524	3774
high_features	basic_models	Surv(days_elapsed, !Alive)	age_scaled	1.52 (1.47-1.57)	< 0.001	0.644	63542.560	12524	3774
high_features	basic_models	Surv(days_elapsed, !Alive)	femaleTRUE	0.77 (0.72-0.82)	< 0.001	0.644	63542.560	12524	3774
high_features	basic_models	Surv(days_elapsed, !Alive)	PRUHTRUE	1.41 (1.31-1.51)	< 0.001	0.644	63542.560	12524	3774
high_features	complex_models	Surv(los, !died_ip)	scale(chron_filab)	0.95 (0.93-0.97)	< 0.001	0.597	272853.769	16011	15731
high_features	complex_models	Surv(los, !died_ip)	age_scaled	0.86 (0.85-0.88)	< 0.001	0.597	272853.769	16011	15731
high_features	complex_models	Surv(los, !died_ip)	femaleTRUE	0.96 (0.93-1.00)	0.02502	0.597	272853.769	16011	15731
high_features	complex_models	Surv(los, !died_ip)	PRUHTRUE	1.31 (1.23-1.39)	< 0.001	0.597	272853.769	16011	15731
high_features	complex_models	Surv(los, !died_ip)	news_scaled	0.86 (0.85-0.87)	< 0.001	0.597	272853.769	16011	15731
high_features	complex_models	Surv(los, !died_ip)	imd_scaled	1.04 (1.01-1.06)	0.00126	0.597	272853.769	16011	15731
high_features	complex_models	Surv(los, !died_ip)	fi_acute_scaled	0.89 (0.87-0.90)	< 0.001	0.597	272853.769	16011	15731
high_features	complex_models	Surv(los, died_ip)	scale(chron_filab)	1.17 (1.01-1.35)	0.03522	0.647	3124.764	5232	240
high_features	complex_models	Surv(los, died_ip)	age_scaled	1.09 (0.96-1.24)	0.17816	0.647	3124.764	5232	240
high_features	complex_models	Surv(los, died_ip)	femaleTRUE	0.95 (0.73-1.23)	0.70391	0.647	3124.764	5232	240
high_features	complex_models	Surv(los, died_ip)	PRUHTRUE	0.42 (0.23-0.78)	0.00604	0.647	3124.764	5232	240
high_features	complex_models	Surv(los, died_ip)	news_scaled	1.26 (1.16-1.37)	< 0.001	0.647	3124.764	5232	240
high_features	complex_models	Surv(los, died_ip)	imd_scaled	0.93 (0.77-1.10)	0.39062	0.647	3124.764	5232	240
high_features	complex_models	Surv(los, died_ip)	fi_acute_scaled	1.29 (1.13-1.47)	< 0.001	0.647	3124.764	5232	240
high_features	complex_models	Surv(days_censored, status_censored)	scale(chron_filab)	1.19 (1.10-1.28)	< 0.001	0.704	11306.121	5232	747
high_features	complex_models	Surv(days_censored, status_censored)	age_scaled	1.33 (1.24-1.43)	< 0.001	0.704	11306.121	5232	747
high_features	complex_models	Surv(days_censored, status_censored)	femaleTRUE	0.86 (0.74-0.99)	0.03892	0.704	11306.121	5232	747
high_features	complex_models	Surv(days_censored, status_censored)	PRUHTRUE	0.62 (0.48-0.80)	< 0.001	0.704	11306.121	5232	747
high_features	complex_models	Surv(days_censored, status_censored)	news_scaled	1.35 (1.29-1.42)	< 0.001	0.704	11306.121	5232	747
high_features	complex_models	Surv(days_censored, status_censored)	imd_scaled	0.94 (0.85-1.03)	0.19203	0.704	11306.121	5232	747
high_features	complex_models	Surv(days_censored, status_censored)	fi_acute_scaled	1.41 (1.31-1.52)	< 0.001	0.704	11306.121	5232	747
high_features	complex_models	Surv(days_elapsed, !Alive)	scale(chron_filab)	1.17 (1.11-1.23)	< 0.001	0.671	20411.368	4750	1389
high_features	complex_models	Surv(days_elapsed, !Alive)	age_scaled	1.49 (1.41-1.57)	< 0.001	0.671	20411.368	4750	1389
high_features	complex_models	Surv(days_elapsed, !Alive)	femaleTRUE	0.77 (0.69-0.86)	< 0.001	0.671	20411.368	4750	1389
high_features	complex_models	Surv(days_elapsed, !Alive)	PRUHTRUE	1.18 (0.99-1.41)	0.05712	0.671	20411.368	4750	1389
high_features	complex_models	Surv(days_elapsed, !Alive)	news_scaled	1.24 (1.19-1.30)	< 0.001	0.671	20411.368	4750	1389
high_features	complex_models	Surv(days_elapsed, !Alive)	imd_scaled	0.95 (0.89-1.02)	0.16636	0.671	20411.368	4750	1389
high_features	complex_models	Surv(days_elapsed, !Alive)	fi_acute_scaled	1.26 (1.19-1.34)	< 0.001	0.671	20411.368	4750	1389
low_features	basic_models	Surv(los, !died_ip)	scale(chron_filab)	0.80 (0.79-0.81)	< 0.001	0.589	1489485.778	74493	72921
low_features	basic_models	Surv(los, !died_ip)	age_scaled	0.88 (0.88-0.89)	< 0.001	0.589	1489485.778	74493	72921
low_features	basic_models	Surv(los, !died_ip)	femaleTRUE	0.94 (0.93-0.96)	< 0.001	0.589	1489485.778	74493	72921
low_features	basic_models	Surv(los, !died_ip)	PRUHTRUE	1.09 (1.07-1.10)	< 0.001	0.589	1489485.778	74493	72921
low_features	basic_models	Surv(los, died_ip)	scale(chron_filab)	1.28 (1.23-1.34)	< 0.001	0.646	23737.066	31353	1394
low_features	basic_models	Surv(los, died_ip)	age_scaled	1.20 (1.14-1.26)	< 0.001	0.646	23737.066	31353	1394
low_features	basic_models	Surv(los, died_ip)	femaleTRUE	0.89 (0.80-1.00)	0.04106	0.646	23737.066	31353	1394
low_features	basic_models	Surv(los, died_ip)	PRUHTRUE	0.41 (0.37-0.47)	< 0.001	0.646	23737.066	31353	1394
low_features	basic_models	Surv(days_censored, status_censored)	scale(chron_filab)	1.35 (1.32-1.38)	< 0.001	0.671	76638.336	31353	4009
low_features	basic_models	Surv(days_censored, status_censored)	age_scaled	1.34 (1.30-1.38)	< 0.001	0.671	76638.336	31353	4009
low_features	basic_models	Surv(days_censored, status_censored)	femaleTRUE	0.84 (0.79-0.89)	< 0.001	0.671	76638.336	31353	4009
low_features	basic_models	Surv(days_censored, status_censored)	PRUHTRUE	0.55 (0.51-0.58)	< 0.001	0.671	76638.336	31353	4009
low_features	basic_models	Surv(days_elapsed, !Alive)	scale(chron_filab)	1.23 (1.21-1.25)	< 0.001	0.648	163335.233	31353	8762
low_features	basic_models	Surv(days_elapsed, !Alive)	age_scaled	1.54 (1.51-1.58)	< 0.001	0.648	163335.233	31353	8762
low_features	basic_models	Surv(days_elapsed, !Alive)	femaleTRUE	0.80 (0.77-0.83)	< 0.001	0.648	163335.233	31353	8762
low_features	basic_models	Surv(days_elapsed, !Alive)	PRUHTRUE	0.80 (0.77-0.84)	< 0.001	0.648	163335.233	31353	8762
low_features	complex_models	Surv(los, !died_ip)	scale(chron_filab)	0.84 (0.83-0.85)	< 0.001	0.607	491574.679	27164	26748
low_features	complex_models	Surv(los, !died_ip)	age_scaled	0.87 (0.85-0.88)	< 0.001	0.607	491574.679	27164	26748
low_features	complex_models	Surv(los, !died_ip)	femaleTRUE	0.94 (0.92-0.96)	< 0.001	0.607	491574.679	27164	26748

low_features	complex_models	Surv(los, !died_ip)	PRUHTRUE	1.27 (1.22-1.31)	< 0.001	0.607	491574.679	27164	26748
low_features	complex_models	Surv(los, !died_ip)	news_scaled	0.87 (0.86-0.88)	< 0.001	0.607	491574.679	27164	26748
low_features	complex_models	Surv(los, !died_ip)	imd_scaled	1.02 (1.00-1.03)	0.03345	0.607	491574.679	27164	26748
low_features	complex_models	Surv(los, !died_ip)	fi_acute_scaled	0.93 (0.92-0.95)	< 0.001	0.607	491574.679	27164	26748
low_features	complex_models	Surv(los, died_ip)	scale(chron_filab)	1.25 (1.12-1.40)	< 0.001	0.704	4916.123	10340	349
low_features	complex_models	Surv(los, died_ip)	age_scaled	1.17 (1.06-1.30)	0.00246	0.704	4916.123	10340	349
low_features	complex_models	Surv(los, died_ip)	femaleTRUE	0.93 (0.75-1.16)	0.51898	0.704	4916.123	10340	349
low_features	complex_models	Surv(los, died_ip)	PRUHTRUE	0.38 (0.26-0.57)	< 0.001	0.704	4916.123	10340	349
low_features	complex_models	Surv(los, died_ip)	news_scaled	1.31 (1.22-1.40)	< 0.001	0.704	4916.123	10340	349
low_features	complex_models	Surv(los, died_ip)	imd_scaled	0.92 (0.80-1.06)	0.23894	0.704	4916.123	10340	349
low_features	complex_models	Surv(los, died_ip)	fi_acute_scaled	1.26 (1.13-1.41)	< 0.001	0.704	4916.123	10340	349
low_features	complex_models	Surv(days_censored, status_censored)	scale(chron_filab)	1.26 (1.20-1.32)	< 0.001	0.726	19969.366	10340	1210
low_features	complex_models	Surv(days_censored, status_censored)	age_scaled	1.33 (1.26-1.41)	< 0.001	0.726	19969.366	10340	1210
low_features	complex_models	Surv(days_censored, status_censored)	femaleTRUE	0.88 (0.78-0.99)	0.03057	0.726	19969.366	10340	1210
low_features	complex_models	Surv(days_censored, status_censored)	PRUHTRUE	0.52 (0.44-0.62)	< 0.001	0.726	19969.366	10340	1210
low_features	complex_models	Surv(days_censored, status_censored)	news_scaled	1.38 (1.32-1.43)	< 0.001	0.726	19969.366	10340	1210
low_features	complex_models	Surv(days_censored, status_censored)	imd_scaled	0.92 (0.86-0.99)	0.03183	0.726	19969.366	10340	1210
low_features	complex_models	Surv(days_censored, status_censored)	fi_acute_scaled	1.32 (1.25-1.40)	< 0.001	0.726	19969.366	10340	1210
low_features	complex_models	Surv(days_elapsed, !Alive)	scale(chron_filab)	1.20 (1.16-1.24)	< 0.001	0.668	39575.812	8907	2480
low_features	complex_models	Surv(days_elapsed, !Alive)	age_scaled	1.50 (1.44-1.56)	< 0.001	0.668	39575.812	8907	2480
low_features	complex_models	Surv(days_elapsed, !Alive)	femaleTRUE	0.84 (0.77-0.91)	< 0.001	0.668	39575.812	8907	2480
low_features	complex_models	Surv(days_elapsed, !Alive)	PRUHTRUE	0.85 (0.77-0.95)	0.00359	0.668	39575.812	8907	2480
low_features	complex_models	Surv(days_elapsed, !Alive)	news_scaled	1.20 (1.16-1.25)	< 0.001	0.668	39575.812	8907	2480
low_features	complex_models	Surv(days_elapsed, !Alive)	imd_scaled	0.93 (0.89-0.98)	0.00785	0.668	39575.812	8907	2480
low_features	complex_models	Surv(days_elapsed, !Alive)	fi_acute_scaled	1.23 (1.18-1.29)	< 0.001	0.668	39575.812	8907	2480
nurse	basic_models	Surv(los, !died_ip)	scale(adjusted_score)	0.78 (0.77-0.79)	< 0.001	0.604	745097.057	39748	38897
nurse	basic_models	Surv(los, !died_ip)	age_scaled	0.93 (0.92-0.93)	< 0.001	0.604	745097.057	39748	38897
nurse	basic_models	Surv(los, !died_ip)	femaleTRUE	1.00 (0.98-1.02)	0.72900	0.604	745097.057	39748	38897
nurse	basic_models	Surv(los, !died_ip)	PRUHTRUE	1.10 (1.07-1.12)	< 0.001	0.604	745097.057	39748	38897
nurse	basic_models	Surv(los, died_ip)	scale(adjusted_score)	1.42 (1.30-1.56)	< 0.001	0.682	11586.689	18420	742
nurse	basic_models	Surv(los, died_ip)	age_scaled	1.08 (1.00-1.16)	0.03689	0.682	11586.689	18420	742
nurse	basic_models	Surv(los, died_ip)	femaleTRUE	0.78 (0.67-0.90)	< 0.001	0.682	11586.689	18420	742
nurse	basic_models	Surv(los, died_ip)	PRUHTRUE	0.45 (0.37-0.55)	< 0.001	0.682	11586.689	18420	742
nurse	basic_models	Surv(days_censored, status_censored)	scale(adjusted_score)	1.71 (1.64-1.79)	< 0.001	0.680	42579.075	18420	2391
nurse	basic_models	Surv(days_censored, status_censored)	age_scaled	1.17 (1.12-1.22)	< 0.001	0.680	42579.075	18420	2391
nurse	basic_models	Surv(days_censored, status_censored)	femaleTRUE	0.73 (0.67-0.79)	< 0.001	0.680	42579.075	18420	2391
nurse	basic_models	Surv(days_censored, status_censored)	PRUHTRUE	0.81 (0.75-0.89)	< 0.001	0.680	42579.075	18420	2391
nurse	basic_models	Surv(days_elapsed, !Alive)	scale(adjusted_score)	1.54 (1.49-1.59)	< 0.001	0.662	70519.300	15826	4147
nurse	basic_models	Surv(days_elapsed, !Alive)	age_scaled	1.36 (1.32-1.40)	< 0.001	0.662	70519.300	15826	4147
nurse	basic_models	Surv(days_elapsed, !Alive)	femaleTRUE	0.72 (0.68-0.77)	< 0.001	0.662	70519.300	15826	4147
nurse	basic_models	Surv(days_elapsed, !Alive)	PRUHTRUE	1.10 (1.03-1.17)	0.00606	0.662	70519.300	15826	4147
nurse	complex_models	Surv(los, !died_ip)	scale(adjusted_score)	0.80 (0.78-0.81)	< 0.001	0.626	340085.751	19581	19217
nurse	complex_models	Surv(los, !died_ip)	age_scaled	0.91 (0.90-0.93)	< 0.001	0.626	340085.751	19581	19217
nurse	complex_models	Surv(los, !died_ip)	femaleTRUE	0.98 (0.96-1.01)	0.24582	0.626	340085.751	19581	19217
nurse	complex_models	Surv(los, !died_ip)	PRUHTRUE	1.21 (1.16-1.27)	< 0.001	0.626	340085.751	19581	19217
nurse	complex_models	Surv(los, !died_ip)	news_scaled	0.86 (0.85-0.88)	< 0.001	0.626	340085.751	19581	19217
nurse	complex_models	Surv(los, !died_ip)	imd_scaled	1.01 (0.99-1.03)	0.45361	0.626	340085.751	19581	19217
nurse	complex_models	Surv(los, !died_ip)	fi_acute_scaled	0.90 (0.89-0.92)	< 0.001	0.626	340085.751	19581	19217
nurse	complex_models	Surv(los, died_ip)	scale(adjusted_score)	1.24 (1.08-1.41)	0.00197	0.693	4172.011	8238	304
nurse	complex_models	Surv(los, died_ip)	age_scaled	1.05 (0.94-1.18)	0.38539	0.693	4172.011	8238	304
nurse	complex_models	Surv(los, died_ip)	femaleTRUE	0.89 (0.70-1.11)	0.29543	0.693	4172.011	8238	304
nurse	complex_models	Surv(los, died_ip)	PRUHTRUE	0.54 (0.35-0.84)	0.00579	0.693	4172.011	8238	304
nurse	complex_models	Surv(los, died_ip)	news_scaled	1.32 (1.22-1.42)	< 0.001	0.693	4172.011	8238	304
nurse	complex_models	Surv(los, died_ip)	imd_scaled	0.95 (0.82-1.11)	0.51477	0.693	4172.011	8238	304
nurse	complex_models	Surv(los, died_ip)	fi_acute_scaled	1.32 (1.17-1.48)	< 0.001	0.693	4172.011	8238	304
nurse	complex_models	Surv(days_censored, status_censored)	scale(adjusted_score)	1.55 (1.45-1.66)	< 0.001	0.726	16337.988	8238	1026
nurse	complex_models	Surv(days_censored, status_censored)	age_scaled	1.16 (1.08-1.23)	< 0.001	0.726	16337.988	8238	1026
nurse	complex_models	Surv(days_censored, status_censored)	femaleTRUE	0.81 (0.71-0.91)	< 0.001	0.726	16337.988	8238	1026
nurse	complex_models	Surv(days_censored, status_censored)	PRUHTRUE	0.78 (0.65-0.94)	0.00965	0.726	16337.988	8238	1026
nurse	complex_models	Surv(days_censored, status_censored)	news_scaled	1.36 (1.30-1.42)	< 0.001	0.726	16337.988	8238	1026
nurse	complex_models	Surv(days_censored, status_censored)	imd_scaled	0.95 (0.88-1.03)	0.24059	0.726	16337.988	8238	1026
nurse	complex_models	Surv(days_censored, status_censored)	fi_acute_scaled	1.36 (1.28-1.44)	< 0.001	0.726	16337.988	8238	1026
nurse	complex_models	Surv(days_elapsed, !Alive)	scale(adjusted_score)	1.45 (1.38-1.53)	< 0.001	0.676	27580.554	6805	1811
nurse	complex_models	Surv(days_elapsed, !Alive)	age_scaled	1.32 (1.26-1.39)	< 0.001	0.676	27580.554	6805	1811
nurse	complex_models	Surv(days_elapsed, !Alive)	femaleTRUE	0.76 (0.70-0.84)	< 0.001	0.676	27580.554	6805	1811
nurse	complex_models	Surv(days_elapsed, !Alive)	PRUHTRUE	1.08 (0.94-1.25)	0.26058	0.676	27580.554	6805	1811

```
|nurse          |complex_models |Surv(days_elapsed, !Alive)            |news_scaled            |1.19 (1.14-1.23) |< 0.001          |0.676            |      27580.554|      6805|      1811|
|nurse          |complex_models |Surv(days_elapsed, !Alive)            |imd_scaled             |0.96 (0.91-1.03) |0.25132          |0.676            |      27580.554|      6805|      1811|
|nurse          |complex_models |Surv(days_elapsed, !Alive)            |fi_acute_scaled        |1.21 (1.16-1.27) |< 0.001          |0.676            |      27580.554|      6805|      1811|
|drug           |basic_models   |Surv(los, !died_ip)                   |scale(chron_fianddrug) |0.94 (0.93-0.96) |< 0.001          |0.534            |     380024.708|     22146|     21056|
|drug           |basic_models   |Surv(los, !died_ip)                   |age_scaled             |0.93 (0.91-0.94) |< 0.001          |0.534            |     380024.708|     22146|     21056|
|drug           |basic_models   |Surv(los, !died_ip)                   |femaleTRUE             |0.99 (0.96-1.01) |0.32628          |0.534            |     380024.708|     22146|     21056|
|drug           |basic_models   |Surv(los, !died_ip)                   |PRUHTRUE               |1.01 (0.98-1.04) |0.52204          |0.534            |     380024.708|     22146|     21056|
|drug           |basic_models   |Surv(los, died_ip)                    |scale(chron_fianddrug) |1.31 (1.24-1.40) |< 0.001          |0.655            |      15059.851|      8757|       944|
|drug           |basic_models   |Surv(los, died_ip)                    |age_scaled             |1.16 (1.09-1.24) |< 0.001          |0.655            |      15059.851|      8757|       944|
|drug           |basic_models   |Surv(los, died_ip)                    |femaleTRUE             |0.93 (0.82-1.06) |0.26879          |0.655            |      15059.851|      8757|       944|
|drug           |basic_models   |Surv(los, died_ip)                    |PRUHTRUE               |0.36 (0.31-0.43) |< 0.001          |0.655            |      15059.851|      8757|       944|
|drug           |basic_models   |Surv(days_censored, status_censored)  |scale(chron_fianddrug) |1.30 (1.25-1.35) |< 0.001          |0.622            |      38894.902|      8757|      2285|
|drug           |basic_models   |Surv(days_censored, status_censored)  |age_scaled             |1.21 (1.16-1.26) |< 0.001          |0.622            |      38894.902|      8757|      2285|
|drug           |basic_models   |Surv(days_censored, status_censored)  |femaleTRUE             |0.86 (0.79-0.94) |< 0.001          |0.622            |      38894.902|      8757|      2285|
|drug           |basic_models   |Surv(days_censored, status_censored)  |PRUHTRUE               |0.58 (0.53-0.63) |< 0.001          |0.622            |      38894.902|      8757|      2285|
|drug           |basic_models   |Surv(days_elapsed, !Alive)            |scale(chron_fianddrug) |1.25 (1.21-1.30) |< 0.001          |0.605            |      53139.103|      8048|      3276|
|drug           |basic_models   |Surv(days_elapsed, !Alive)            |age_scaled             |1.39 (1.34-1.44) |< 0.001          |0.605            |      53139.103|      8048|      3276|
|drug           |basic_models   |Surv(days_elapsed, !Alive)            |femaleTRUE             |0.84 (0.79-0.90) |< 0.001          |0.605            |      53139.103|      8048|      3276|
|drug           |basic_models   |Surv(days_elapsed, !Alive)            |PRUHTRUE               |0.84 (0.78-0.90) |< 0.001          |0.605            |      53139.103|      8048|      3276|
|drug           |complex_models |Surv(los, !died_ip)                   |scale(chron_fianddrug) |0.97 (0.95-1.00) |0.02041          |0.538            |     128894.121|      8357|      8001|
|drug           |complex_models |Surv(los, !died_ip)                   |age_scaled             |0.91 (0.89-0.93) |< 0.001          |0.538            |     128894.121|      8357|      8001|
|drug           |complex_models |Surv(los, !died_ip)                   |femaleTRUE             |0.97 (0.93-1.02) |0.23222          |0.538            |     128894.121|      8357|      8001|
|drug           |complex_models |Surv(los, !died_ip)                   |PRUHTRUE               |1.04 (0.97-1.11) |0.27302          |0.538            |     128894.121|      8357|      8001|
|drug           |complex_models |Surv(los, !died_ip)                   |news_scaled            |0.98 (0.96-1.00) |0.01401          |0.538            |     128894.121|      8357|      8001|
|drug           |complex_models |Surv(los, !died_ip)                   |imd_scaled             |1.01 (0.98-1.04) |0.56359          |0.538            |     128894.121|      8357|      8001|
|drug           |complex_models |Surv(los, !died_ip)                   |fi_acute_scaled        |0.93 (0.91-0.96) |< 0.001          |0.538            |     128894.121|      8357|      8001|
|drug           |complex_models |Surv(los, died_ip)                    |scale(chron_fianddrug) |1.12 (0.99-1.26) |0.06912          |0.667            |       3991.331|      3113|       292|
|drug           |complex_models |Surv(los, died_ip)                    |age_scaled             |1.17 (1.04-1.31) |0.00795          |0.667            |       3991.331|      3113|       292|
|drug           |complex_models |Surv(los, died_ip)                    |femaleTRUE             |0.94 (0.74-1.18) |0.57366          |0.667            |       3991.331|      3113|       292|
|drug           |complex_models |Surv(los, died_ip)                    |PRUHTRUE               |0.44 (0.28-0.69) |< 0.001          |0.667            |       3991.331|      3113|       292|
|drug           |complex_models |Surv(los, died_ip)                    |news_scaled            |1.29 (1.20-1.40) |< 0.001          |0.667            |       3991.331|      3113|       292|
|drug           |complex_models |Surv(los, died_ip)                    |imd_scaled             |0.92 (0.79-1.08) |0.31398          |0.667            |       3991.331|      3113|       292|
|drug           |complex_models |Surv(los, died_ip)                    |fi_acute_scaled        |1.25 (1.10-1.42) |< 0.001          |0.667            |       3991.331|      3113|       292|
|drug           |complex_models |Surv(days_censored, status_censored)  |scale(chron_fianddrug) |1.17 (1.09-1.26) |< 0.001          |0.655            |      11563.545|      3113|       781|
|drug           |complex_models |Surv(days_censored, status_censored)  |age_scaled             |1.24 (1.15-1.33) |< 0.001          |0.655            |      11563.545|      3113|       781|
|drug           |complex_models |Surv(days_censored, status_censored)  |femaleTRUE             |0.86 (0.75-0.99) |0.03983          |0.655            |      11563.545|      3113|       781|
|drug           |complex_models |Surv(days_censored, status_censored)  |PRUHTRUE               |0.63 (0.51-0.79) |< 0.001          |0.655            |      11563.545|      3113|       781|
|drug           |complex_models |Surv(days_censored, status_censored)  |news_scaled            |1.25 (1.19-1.31) |< 0.001          |0.655            |      11563.545|      3113|       781|
|drug           |complex_models |Surv(days_censored, status_censored)  |imd_scaled             |0.94 (0.85-1.03) |0.16225          |0.655            |      11563.545|      3113|       781|
|drug           |complex_models |Surv(days_censored, status_censored)  |fi_acute_scaled        |1.24 (1.15-1.34) |< 0.001          |0.655            |      11563.545|      3113|       781|
|drug           |complex_models |Surv(days_elapsed, !Alive)            |scale(chron_fianddrug) |1.15 (1.08-1.23) |< 0.001          |0.625            |      16321.498|      2794|      1164|
|drug           |complex_models |Surv(days_elapsed, !Alive)            |age_scaled             |1.39 (1.31-1.48) |< 0.001          |0.625            |      16321.498|      2794|      1164|
|drug           |complex_models |Surv(days_elapsed, !Alive)            |femaleTRUE             |0.84 (0.75-0.94) |0.00354          |0.625            |      16321.498|      2794|      1164|
|drug           |complex_models |Surv(days_elapsed, !Alive)            |PRUHTRUE               |0.80 (0.68-0.95) |0.01036          |0.625            |      16321.498|      2794|      1164|
|drug           |complex_models |Surv(days_elapsed, !Alive)            |news_scaled            |1.16 (1.10-1.21) |< 0.001          |0.625            |      16321.498|      2794|      1164|
|drug           |complex_models |Surv(days_elapsed, !Alive)            |imd_scaled             |0.96 (0.89-1.03) |0.23449          |0.625            |      16321.498|      2794|      1164|
|drug           |complex_models |Surv(days_elapsed, !Alive)            |fi_acute_scaled        |1.20 (1.13-1.28) |< 0.001          |0.625            |      16321.498|      2794|      1164|
```

In [47]:

```
compare_mixed_effects_simple <- function(results) {
  fi_versions <- c("base", "short_period", "mean_type", "high_features", "low_features", "nurse", "drug")

  # Create multi_measurements for each version
  multi_measurements <- map(fi_versions, function(version) {
    df <- results[[version]]$datasets$los

    fi_col <- case_when(
      version == "nurse" ~ "adjusted_score",
      version == "drug" ~ "chron_fianddrug",
      TRUE ~ "chron_filab"
    )

    df %>%
      group_by(client_idcode) %>%
      filter(n() > 1) %>%
      arrange(time) %>%
      mutate(
```

```
      measurement = row_number(),
      time_passed = time - min(time)
    ) %>%
    ungroup()
}) %>% setNames(fi_versions)

# Fit models for each version
models <- map2(fi_versions, multi_measurements, function(version, data) {
  fi_col <- case_when(
    version == "nurse" ~ "adjusted_score",
    version == "drug" ~ "chron_fianddrug",
    TRUE ~ "chron_filab"
  )

  formula <- as.formula(
    paste0(fi_col, " ~ female + age_scaled + PRUH + presenting_complaint + (1 | client_idcode)")
  )

  model <- lmer(formula, data = data)

  # Get ICC without converting to data frame
  icc_val <- performance::icc(model)$ICC_adjusted

  # Get R2 values
  r2_vals <- performance::r2_nakagawa(model)

  list(
    model = model,
    n_patients = length(unique(data$client_idcode)),
    n_observations = nrow(data),
    icc = icc_val,
    r2_conditional = r2_vals$R2_conditional,
    r2_marginal = r2_vals$R2_marginal,
    aic = AIC(model),
    bic = BIC(model)
  )
}) %>% setNames(fi_versions)

# Compare fixed effects
fixed_effects <- map_dfr(models, function(m) {
  coefs <- summary(m$model)$coefficients
  data.frame(
    term = rownames(coefs),
    estimate = coefs[,"Estimate"],
    std_error = coefs[,"Std. Error"],
    t_value = coefs[,"t value"]
  )
}, .id = "version")

# Compare random effects
random_effects <- map_dfr(models, function(m) {
  data.frame(
    variance = as.numeric(VarCorr(m$model)$client_idcode[1])
  )
}, .id = "version")

# Compile model fit statistics
model_stats <- map_dfr(models, function(m) {
  data.frame(
    n_patients = m$n_patients,
    n_observations = m$n_observations,
    icc = m$icc,
    r2_conditional = m$r2_conditional,
    r2_marginal = m$r2_marginal,
    aic = m$aic,
    bic = m$bic
```

```
    )
  }, .id = "version")

  list(
    multi_measurements = multi_measurements,
    models = models,
    fixed_effects = fixed_effects,
    random_effects = random_effects,
    model_stats = model_stats
  )
}
```

In [68]:
```
compare_mixed_effects_complex <- function(results) {
  fi_versions <- c("base", "short_period", "mean_type", "high_features", "low_features", "nurse", "drug")

  # Create multi_measurements for each version
  multi_measurements <- map(fi_versions, function(version) {
    df <- results[[version]]$datasets$los

    fi_col <- case_when(
      version == "nurse" ~ "adjusted_score",
      version == "drug" ~ "chron_fianddrug",
      TRUE ~ "chron_filab"
    )

    df %>%
      group_by(client_idcode) %>%
      filter(n() > 1) %>%
      arrange(time) %>%
      mutate(
        measurement = row_number(),
        time_passed = time - min(time)
      ) %>%
      ungroup()
  }) %>% setNames(fi_versions)

  # Fit models for each version
  models <- map2(fi_versions, multi_measurements, function(version, data) {
    fi_col <- case_when(
      version == "nurse" ~ "adjusted_score",
      version == "drug" ~ "chron_fianddrug",
      TRUE ~ "chron_filab"
    )

    formula <- as.formula(
      paste0(fi_col, " ~ female + age_scaled + PRUH + presenting_complaint + news_scaled + fi_acute_scaled + (1 | client_idcode)")
    )

    model <- lmer(formula, data = data)


      # Get the full ICC results object including CIs
icc_results <- performance::icc(model, ci = 0.95) # ci = 0.95 is often the default, but good to be explicit


icc_val <- icc_results['1', 'ICC_adjusted']
icc_ci_low <- icc_results['CI_low', 'ICC_adjusted']
icc_ci_high <- icc_results['CI_high', 'ICC_adjusted']


    # Get R2 values
    r2_vals <- performance::r2_nakagawa(model)

    list(
      model = model,
```

```r
      n_patients = length(unique(data$client_idcode)),
      n_observations = nrow(data),
      icc = icc_val,
        icc_ci_low = icc_ci_low,
  icc_ci_high = icc_ci_high,
      r2_conditional = r2_vals$R2_conditional,
      r2_marginal = r2_vals$R2_marginal,
      aic = AIC(model),
      bic = BIC(model)
    )
  }) %>% setNames(fi_versions)

  # Compare fixed effects
  fixed_effects <- map_dfr(models, function(m) {
    coefs <- summary(m$model)$coefficients
    data.frame(
      term = rownames(coefs),
      estimate = coefs[,"Estimate"],
      std_error = coefs[,"Std. Error"],
      t_value = coefs[,"t value"]
    )
  }, .id = "version")

  # Compare random effects
  random_effects <- map_dfr(models, function(m) {
    data.frame(
      variance = as.numeric(VarCorr(m$model)$client_idcode[1])
    )
  }, .id = "version")

  # Compile model fit statistics
  model_stats <- map_dfr(models, function(m) {
    data.frame(
      n_patients = m$n_patients,
      n_observations = m$n_observations,
      icc = m$icc,
        icc_ci_low = m$icc_ci_low,
        icc_ci_high = m$icc_ci_high,
      r2_conditional = m$r2_conditional,
      r2_marginal = m$r2_marginal,
      aic = m$aic,
      bic = m$bic
    )
  }, .id = "version")

  list(
    multi_measurements = multi_measurements,
    models = models,
    fixed_effects = fixed_effects,
    random_effects = random_effects,
    model_stats = model_stats
  )
}
```

In [45]:
```r
comparison_results  <- compare_mixed_effects_simple(results)
```

In [69]:
```r
complex_comparison_results <- compare_mixed_effects_complex(results)
```

```
Warning message in data.frame(n_patients = m$n_patients, n_observations = m$n_observations, :
“row names were found from a short variable and have been discarded”
Warning message in data.frame(n_patients = m$n_patients, n_observations = m$n_observations, :
“row names were found from a short variable and have been discarded”
Warning message in data.frame(n_patients = m$n_patients, n_observations = m$n_observations, :
“row names were found from a short variable and have been discarded”
Warning message in data.frame(n_patients = m$n_patients, n_observations = m$n_observations, :
“row names were found from a short variable and have been discarded”
Warning message in data.frame(n_patients = m$n_patients, n_observations = m$n_observations, :
“row names were found from a short variable and have been discarded”
Warning message in data.frame(n_patients = m$n_patients, n_observations = m$n_observations, :
“row names were found from a short variable and have been discarded”
Warning message in data.frame(n_patients = m$n_patients, n_observations = m$n_observations, :
“row names were found from a short variable and have been discarded”
```

In [ ]:

```
plot_mixed_effects_results <- function(comparison_results) {
  # Model fit statistics plot
  plot_model_stats <- function(model_stats) {
    model_stats %>%
      pivot_longer(cols = c(icc, r2_conditional, r2_marginal),
                   names_to = "metric", values_to = "value") %>%
      ggplot(aes(x = version, y = value, fill = metric)) +
      geom_bar(stat = "identity", position = "dodge") +
      theme_minimal() +
      theme(axis.text.x = element_text(angle = 45, hjust = 1)) +
      labs(title = "Model Fit Comparison",
           x = "FI Version",
           y = "Value",
           fill = "Metric")
  }

  # Fixed effects plot
  plot_fixed_effects <- function(fixed_effects) {
    fixed_effects %>%
      filter(term != "(Intercept)") %>%
      ggplot(aes(x = version, y = estimate,
                 ymin = estimate - 1.96*std_error,
                 ymax = estimate + 1.96*std_error)) +
      geom_pointrange() +
      facet_wrap(~term, scales = "free_y") +
      theme_minimal() +
      theme(axis.text.x = element_text(angle = 45, hjust = 1)) +
      labs(title = "Fixed Effects Estimates",
           x = "FI Version",
           y = "Estimate with 95% CI")
  }

  # Sample size comparison
  plot_sample_sizes <- function(model_stats) {
    model_stats %>%
      pivot_longer(cols = c(n_patients, n_observations),
                   names_to = "count_type", values_to = "count") %>%
      ggplot(aes(x = version, y = count, fill = count_type)) +
      geom_bar(stat = "identity", position = "dodge") +
      theme_minimal() +
      theme(axis.text.x = element_text(angle = 45, hjust = 1)) +
      labs(title = "Sample Size Comparison",
           x = "FI Version",
           y = "Count",
           fill = "Type")
  }

  # Random effects variance plot
  plot_random_effects <- function(random_effects) {
    random_effects %>%
```

```r
      ggplot(aes(x = version, y = variance)) +
      geom_bar(stat = "identity") +
      theme_minimal() +
      theme(axis.text.x = element_text(angle = 45, hjust = 1)) +
      labs(title = "Random Effects Variance by FI Version",
           x = "FI Version",
           y = "Variance")
  }

  list(
    model_stats_plot = plot_model_stats(comparison_results$model_stats),
    fixed_effects_plot = plot_fixed_effects(comparison_results$fixed_effects),
    sample_sizes_plot = plot_sample_sizes(comparison_results$model_stats),
    random_effects_plot = plot_random_effects(comparison_results$random_effects)
  )
}
```

In [ ]:
```r
plots <- plot_mixed_effects_results(comparison_results)
gridExtra::grid.arrange(plots$model_stats_plot, plots$fixed_effects_plot,
                       plots$sample_sizes_plot, plots$random_effects_plot,
                       ncol = 2)
```

In [ ]:
```r
plots$fixed_effects_plot
```

In [151…
```r
# Get sample characteristics
sample_summary_simple <- map_dfr(comparison_results$multi_measurements, function(df) {
  data.frame(
    n_total = length(unique(df$client_idcode)),
    n_measurements = nrow(df),
    avg_measures_per_patient = mean(table(df$client_idcode)),
    median_time_between = median(df$time_passed)
  )
}, .id = "version")

sample_summary_complex <- map_dfr(complex_comparison_results$multi_measurements, function(df) {
  data.frame(
    n_total = length(unique(df$client_idcode)),
    n_measurements = nrow(df),
    avg_measures_per_patient = mean(table(df$client_idcode)),
    median_time_between = median(df$time_passed)
  )
}, .id = "version")

# Get model statistics summary
model_summary_simple <- comparison_results$model_stats
model_summary_complex <- complex_comparison_results$model_stats


# Get fixed effects summary
effects_summary_simple <- comparison_results$fixed_effects
effects_summary_complex <- complex_comparison_results$fixed_effects
```

In [122…
```r
print(complex_comparison_results$model_stats)
```

```
                           version n_patients n_observations       icc icc_ci_low
Conditional R2...1          base      12766          49191 0.6006793  0.5899371
Conditional R2...2  short_period      12766          49191 0.6020618  0.5895143
Conditional R2...3     mean_type      12766          49191 0.5937721  0.5827490
Conditional R2...4 high_features       7352          31095 0.6747570  0.6644009
Conditional R2...5  low_features      15829          58969 0.5125709  0.4994183
Conditional R2...6         nurse      12766          49191 0.3748198  0.3588938
Conditional R2...7          drug      12766          49191 0.7564620  0.7466013
                   icc_ci_high r2_conditional r2_marginal       aic       bic
Conditional R2...1   0.6151451      0.6311548  0.07631831 -38587.63 -38269.34
Conditional R2...2   0.6163408      0.6339531  0.08014136 -37706.92 -37388.62
Conditional R2...3   0.6087098      0.6283717  0.08517292 -36875.46 -36557.16
Conditional R2...4   0.6886666      0.6940328  0.05926603 -27562.03 -27258.83
Conditional R2...5   0.5272487      0.5545607  0.08614553 -39475.64 -39152.81
Conditional R2...6   0.3905267      0.4752404  0.16062673  61589.72  61897.72
Conditional R2...7   0.7716515      0.7720738  0.06410423 -17865.96 -17589.83
```

In [71]:
```
sample_summary_simple
```

A data.frame: 7 × 5

version	n_total	n_measurements	avg_measures_per_patient	median_time_between
<chr>	<int>	<int>	<dbl>	<drtn>
base	12766	49191	3.853282	14015299 secs
short_period	12766	49191	3.853282	14015299 secs
mean_type	12766	49191	3.853282	14015299 secs
high_features	7352	31095	4.229461	17371166 secs
low_features	15829	58969	3.725377	13501466 secs
nurse	12766	49191	3.853282	14015299 secs
drug	12766	49191	3.853282	14015299 secs

In [148…
```
sample_summary_complex
```

A data.frame: 7 × 5

version	n_total	n_measurements	avg_measures_per_patient	median_time_between
<chr>	<int>	<int>	<dbl>	<drtn>
base	12766	49191	3.853282	14015299 secs
short_period	12766	49191	3.853282	14015299 secs
mean_type	12766	49191	3.853282	14015299 secs
high_features	7352	31095	4.229461	17371166 secs
low_features	15829	58969	3.725377	13501466 secs
nurse	12766	49191	3.853282	14015299 secs
drug	12766	49191	3.853282	14015299 secs

In [152…
```
model_summary_complex
```

A data.frame: 7 × 10

	version	n_patients	n_observations	icc	icc_ci_low	icc_ci_high	r2_conditional	r2_marginal	aic	bic
	<chr>	<int>	<int>	<dbl>	<dbl>	<dbl>	<dbl>	<dbl>	<dbl>	<dbl>
**Conditional R2...1**	base	12766	49191	0.6006793	0.5899371	0.6151451	0.6311548	0.07631831	-38587.63	-38269.34
**Conditional R2...2**	short_period	12766	49191	0.6020618	0.5895143	0.6163408	0.6339531	0.08014136	-37706.92	-37388.62
**Conditional R2...3**	mean_type	12766	49191	0.5937721	0.5827490	0.6087098	0.6283717	0.08517292	-36875.46	-36557.16
**Conditional R2...4**	high_features	7352	31095	0.6747570	0.6644009	0.6886666	0.6940328	0.05926603	-27562.03	-27258.83
**Conditional R2...5**	low_features	15829	58969	0.5125709	0.4994183	0.5272487	0.5545607	0.08614553	-39475.64	-39152.81
**Conditional R2...6**	nurse	12766	49191	0.3748198	0.3588938	0.3905267	0.4752404	0.16062673	61589.72	61897.72
**Conditional R2...7**	drug	12766	49191	0.7564620	0.7466013	0.7716515	0.7720738	0.06410423	-17865.96	-17589.83

In [157…

```
kable(effects_summary_complex, row.names = FALSE)
```

version	term	estimate	std_error	t_value
base	(Intercept)	0.1863134	0.0119743	15.5594509
base	femaleTRUE	-0.0377219	0.0021961	-17.1763935
base	age_scaled	0.0084893	0.0011086	7.6578130
base	PRUHTRUE	0.0009176	0.0023933	0.3833977
base	presenting_complaintAirway / breathing >> Difficulty breathing	-0.0030249	0.0127498	-0.2372534
base	presenting_complaintAirway / breathing >> Short of breath	-0.0040178	0.0120166	-0.3343539
base	presenting_complaintCirculation / chest	-0.0080782	0.0129982	-0.6214860
base	presenting_complaintCirculation / chest >> Chest pain	-0.0085858	0.0120617	-0.7118216
base	presenting_complaintCirculation / chest >> Collapse / fainting episode	-0.0069963	0.0128823	-0.5430909
base	presenting_complaintCirculation / chest >> Palpitations	-0.0163678	0.0129401	-1.2648888
base	presenting_complaintEnvironmental	-0.0010333	0.0172287	-0.0599735
base	presenting_complaintEye	-0.0002330	0.0148476	-0.0156914
base	presenting_complaintGastrointestinal	0.0071916	0.0121453	0.5921269
base	presenting_complaintGastrointestinal >> Abdominal pain	0.0047894	0.0120612	0.3970896
base	presenting_complaintGeneral / minor / admin	0.0029831	0.0123420	0.2417067
base	presenting_complaintGeneral / minor / admin >> Generalised weakness	0.0040147	0.0121183	0.3312965
base	presenting_complaintGenitourinary	0.0000367	0.0121442	0.0030235
base	presenting_complaintGenitourinary >> Unable to pass urine	-0.0026633	0.0124856	-0.2133066
base	presenting_complaintHead and neck	-0.0121277	0.0131060	-0.9253540
base	presenting_complaintNeurological	-0.0044237	0.0125179	-0.3533923
base	presenting_complaintNeurological >> Confusion	-0.0074881	0.0124682	-0.6005796
base	presenting_complaintNeurological >> Dizziness	-0.0079451	0.0123880	-0.6413564
base	presenting_complaintNeurological >> Falls / unsteady on feet	-0.0070552	0.0120798	-0.5840464
base	presenting_complaintNeurological >> Headache	-0.0052521	0.0126468	-0.4152940
base	presenting_complaintNeurological >> Limb weakness	-0.0080303	0.0132953	-0.6039899
base	presenting_complaintNeurological >> Speech disturbance	0.0041421	0.0141318	0.2931050
base	presenting_complaintObGyn	-0.0137799	0.0187495	-0.7349461
base	presenting_complaintOther	0.0019744	0.0125936	0.1567742
base	presenting_complaintPsychosocial / Behaviour change	0.0008542	0.0134960	0.0632905
base	presenting_complaintSkin	-0.0018451	0.0137279	-0.1344039
base	presenting_complaintSkin >> Localised swelling / redness / lumps / bumps	0.0003563	0.0127669	0.0279100
base	presenting_complaintTrauma / musculoskeletal	-0.0035112	0.0122464	-0.2867141
base	presenting_complaintTrauma / musculoskeletal >> Head injury	-0.0057033	0.0124516	-0.4580362
base	presenting_complaintTrauma / musculoskeletal >> Injury of lower limb	0.0001261	0.0128839	0.0097890
base	presenting_complaintTrauma / musculoskeletal >> Pain in lower limb	-0.0082557	0.0123309	-0.6695110
base	presenting_complaintUnwell Adult	0.0055060	0.0129633	0.4247386
base	news_scaled	0.0031767	0.0006559	4.8430906
base	fi_acute_scaled	0.0223298	0.0007345	30.4023372
short_period	(Intercept)	0.1930614	0.0122209	15.7976541
short_period	femaleTRUE	-0.0385899	0.0022452	-17.1879789
short_period	age_scaled	0.0096643	0.0011332	8.5280857
short_period	PRUHTRUE	-0.0063292	0.0024461	-2.5874234
short_period	presenting_complaintAirway / breathing >> Difficulty breathing	0.0002329	0.0130118	0.0178971
short_period	presenting_complaintAirway / breathing >> Short of breath	-0.0024480	0.0122636	-0.1996135
short_period	presenting_complaintCirculation / chest	-0.0045013	0.0132655	-0.3393245
short_period	presenting_complaintCirculation / chest >> Chest pain	-0.0059733	0.0123097	-0.4852564
short_period	presenting_complaintCirculation / chest >> Collapse / fainting episode	-0.0048287	0.0131473	-0.3672740
short_period	presenting_complaintCirculation / chest >> Palpitations	-0.0150865	0.0132064	-1.1423630
short_period	presenting_complaintEnvironmental	0.0050915	0.0175823	0.2895834
short_period	presenting_complaintEye	0.0007750	0.0151533	0.0511434
short_period	presenting_complaintGastrointestinal	0.0085807	0.0123950	0.6922679
short_period	presenting_complaintGastrointestinal >> Abdominal pain	0.0061524	0.0123091	0.4998266
short_period	presenting_complaintGeneral / minor / admin	0.0054239	0.0125957	0.4306148
short_period	presenting_complaintGeneral / minor / admin >> Generalised weakness	0.0045503	0.0123675	0.3679253
short_period	presenting_complaintGenitourinary	0.0021314	0.0123939	0.1719726
short_period	presenting_complaintGenitourinary >> Unable to pass urine	0.0004798	0.0127424	0.0376571
short_period	presenting_complaintHead and neck	-0.0074419	0.0133758	-0.5563720
short_period	presenting_complaintNeurological	0.0000711	0.0127754	0.0055692
short_period	presenting_complaintNeurological >> Confusion	-0.0064366	0.0127246	-0.5058365
short_period	presenting_complaintNeurological >> Dizziness	-0.0048756	0.0126427	-0.3856489
short_period	presenting_complaintNeurological >> Falls / unsteady on feet	-0.0044934	0.0123282	-0.3644833
short_period	presenting_complaintNeurological >> Headache	-0.0032839	0.0129068	-0.2544323
short_period	presenting_complaintNeurological >> Limb weakness	-0.0079756	0.0135689	-0.5877838

short_period	presenting_complaintNeurological >> Speech disturbance	0.0010873	0.0144228	0.0753841
short_period	presenting_complaintObGyn	-0.0113851	0.0191356	-0.5949675
short_period	presenting_complaintOther	-0.0001855	0.0128525	-0.0144302
short_period	presenting_complaintPsychosocial / Behaviour change	0.0042317	0.0137737	0.3072342
short_period	presenting_complaintSkin	0.0029286	0.0140103	0.2090289
short_period	presenting_complaintSkin >> Localised swelling / redness / lumps / bumps	0.0043058	0.0130297	0.3304610
short_period	presenting_complaintTrauma / musculoskeletal	-0.0018371	0.0124982	-0.1469919
short_period	presenting_complaintTrauma / musculoskeletal >> Head injury	-0.0037284	0.0127076	-0.2933959
short_period	presenting_complaintTrauma / musculoskeletal >> Injury of lower limb	0.0008170	0.0131489	0.0621316
short_period	presenting_complaintTrauma / musculoskeletal >> Pain in lower limb	-0.0053105	0.0125845	-0.4219874
short_period	presenting_complaintUnwell Adult	0.0072587	0.0132298	0.5486630
short_period	news_scaled	0.0031184	0.0006695	4.6576320
short_period	fi_acute_scaled	0.0240236	0.0007498	32.0394849
mean_type	(Intercept)	0.2195951	0.0125026	17.5639388
mean_type	femaleTRUE	-0.0434980	0.0022739	-19.1290801
mean_type	age_scaled	0.0097061	0.0011483	8.4522664
mean_type	PRUHTRUE	-0.0026050	0.0024812	-1.0499057
mean_type	presenting_complaintAirway / breathing >> Difficulty breathing	0.0015349	0.0133154	0.1152710
mean_type	presenting_complaintAirway / breathing >> Short of breath	-0.0021953	0.0125490	-0.1749341
mean_type	presenting_complaintCirculation / chest	-0.0033748	0.0135736	-0.2486314
mean_type	presenting_complaintCirculation / chest >> Chest pain	-0.0081367	0.0125961	-0.6459714
mean_type	presenting_complaintCirculation / chest >> Collapse / fainting episode	-0.0097140	0.0134520	-0.7221276
mean_type	presenting_complaintCirculation / chest >> Palpitations	-0.0133602	0.0135121	-0.9887578
mean_type	presenting_complaintEnvironmental	-0.0032669	0.0179948	-0.1815469
mean_type	presenting_complaintEye	0.0017359	0.0155031	0.1119708
mean_type	presenting_complaintGastrointestinal	0.0063754	0.0126833	0.5026603
mean_type	presenting_complaintGastrointestinal >> Abdominal pain	0.0054330	0.0125956	0.4313455
mean_type	presenting_complaintGeneral / minor / admin	0.0044212	0.0128886	0.3430301
mean_type	presenting_complaintGeneral / minor / admin >> Generalised weakness	0.0061728	0.0126550	0.4877751
mean_type	presenting_complaintGenitourinary	0.0010151	0.0126820	0.0800453
mean_type	presenting_complaintGenitourinary >> Unable to pass urine	-0.0020265	0.0130380	-0.1554266
mean_type	presenting_complaintHead and neck	-0.0114395	0.0136846	-0.8359360
mean_type	presenting_complaintNeurological	-0.0049138	0.0130720	-0.3759014
mean_type	presenting_complaintNeurological >> Confusion	-0.0076629	0.0130200	-0.5885484
mean_type	presenting_complaintNeurological >> Dizziness	-0.0049365	0.0129369	-0.3815819
mean_type	presenting_complaintNeurological >> Falls / unsteady on feet	-0.0073727	0.0126149	-0.5844409
mean_type	presenting_complaintNeurological >> Headache	-0.0079754	0.0132066	-0.6038982
mean_type	presenting_complaintNeurological >> Limb weakness	-0.0049884	0.0138832	-0.3593102
mean_type	presenting_complaintNeurological >> Speech disturbance	0.0073306	0.0147554	0.4968096
mean_type	presenting_complaintObGyn	-0.0008698	0.0195766	-0.0444299
mean_type	presenting_complaintOther	-0.0000458	0.0131515	-0.0034797
mean_type	presenting_complaintPsychosocial / Behaviour change	-0.0064806	0.0140928	-0.4598508
mean_type	presenting_complaintSkin	-0.0025495	0.0143351	-0.1778491
mean_type	presenting_complaintSkin >> Localised swelling / redness / lumps / bumps	0.0013397	0.0133308	0.1004957
mean_type	presenting_complaintTrauma / musculoskeletal	-0.0017864	0.0127887	-0.1396826
mean_type	presenting_complaintTrauma / musculoskeletal >> Head injury	-0.0078733	0.0130027	-0.6055122
mean_type	presenting_complaintTrauma / musculoskeletal >> Injury of lower limb	-0.0009737	0.0134541	-0.0723712
mean_type	presenting_complaintTrauma / musculoskeletal >> Pain in lower limb	-0.0076162	0.0128768	-0.5914662
mean_type	presenting_complaintUnwell Adult	0.0142870	0.0135374	1.0553743
mean_type	news_scaled	0.0033125	0.0006844	4.8397846
mean_type	fi_acute_scaled	0.0238533	0.0007658	31.1477641
high_features	(Intercept)	0.1654226	0.0132851	12.4517447
high_features	femaleTRUE	-0.0402352	0.0029150	-13.8030395
high_features	age_scaled	0.0057026	0.0014679	3.8849918
high_features	PRUHTRUE	0.0335285	0.0043265	7.7496312
high_features	presenting_complaintAirway / breathing >> Difficulty breathing	-0.0051109	0.0140300	-0.3642817
high_features	presenting_complaintAirway / breathing >> Short of breath	-0.0044024	0.0132809	-0.3314805
high_features	presenting_complaintCirculation / chest	-0.0074893	0.0144526	-0.5181971
high_features	presenting_complaintCirculation / chest >> Chest pain	-0.0063453	0.0133335	-0.4758918
high_features	presenting_complaintCirculation / chest >> Collapse / fainting episode	-0.0086812	0.0143311	-0.6057643
high_features	presenting_complaintCirculation / chest >> Palpitations	-0.0107097	0.0142841	-0.7497643
high_features	presenting_complaintEnvironmental	-0.0018119	0.0193097	-0.0938354
high_features	presenting_complaintEye	0.0045942	0.0165504	0.2775906
high_features	presenting_complaintGastrointestinal	0.0068102	0.0134459	0.5064873
high_features	presenting_complaintGastrointestinal >> Abdominal pain	0.0014522	0.0133390	0.1088657
high_features	presenting_complaintGeneral / minor / admin	-0.0029989	0.0136579	-0.2195750

high_features	presenting_complaintGeneral / minor / admin >> Generalised weakness	0.0016478	0.0134287	0.1227103
high_features	presenting_complaintGenitourinary	-0.0001446	0.0134489	-0.0107532
high_features	presenting_complaintGenitourinary >> Unable to pass urine	-0.0022646	0.0138819	-0.1631319
high_features	presenting_complaintHead and neck	-0.0087521	0.0144947	-0.6038143
high_features	presenting_complaintNeurological	-0.0067356	0.0138517	-0.4862668
high_features	presenting_complaintNeurological >> Confusion	-0.0065800	0.0138437	-0.4753062
high_features	presenting_complaintNeurological >> Dizziness	-0.0083806	0.0136922	-0.6120746
high_features	presenting_complaintNeurological >> Falls / unsteady on feet	-0.0087004	0.0133730	-0.6505956
high_features	presenting_complaintNeurological >> Headache	-0.0095572	0.0139835	-0.6834650
high_features	presenting_complaintNeurological >> Limb weakness	-0.0116947	0.0148379	-0.7881668
high_features	presenting_complaintNeurological >> Speech disturbance	-0.0094247	0.0157674	-0.5977317
high_features	presenting_complaintObGyn	-0.0154423	0.0204092	-0.7566331
high_features	presenting_complaintOther	-0.0090429	0.0138898	-0.6510472
high_features	presenting_complaintPsychosocial / Behaviour change	0.0044990	0.0149946	0.3000391
high_features	presenting_complaintSkin	-0.0059020	0.0151687	-0.3890902
high_features	presenting_complaintSkin >> Localised swelling / redness / lumps / bumps	-0.0108899	0.0142182	-0.7659161
high_features	presenting_complaintTrauma / musculoskeletal	-0.0054047	0.0135547	-0.3987319
high_features	presenting_complaintTrauma / musculoskeletal >> Head injury	-0.0068341	0.0138122	-0.4947866
high_features	presenting_complaintTrauma / musculoskeletal >> Injury of lower limb	-0.0011937	0.0143363	-0.0832616
high_features	presenting_complaintTrauma / musculoskeletal >> Pain in lower limb	-0.0075684	0.0136493	-0.5544930
high_features	presenting_complaintUnwell Adult	0.0000106	0.0142415	0.0007414
high_features	news_scaled	0.0006872	0.0007495	0.9168737
high_features	fi_acute_scaled	0.0154726	0.0008624	17.9418958
low_features	(Intercept)	0.1988074	0.0125110	15.8905699
low_features	femaleTRUE	-0.0345933	0.0020485	-16.8872122
low_features	age_scaled	0.0084129	0.0010355	8.1244491
low_features	PRUHTRUE	-0.0065005	0.0021727	-2.9918524
low_features	presenting_complaintAirway / breathing >> Difficulty breathing	0.0029881	0.0134072	0.2228694
low_features	presenting_complaintAirway / breathing >> Short of breath	0.0051648	0.0125822	0.4104794
low_features	presenting_complaintCirculation / chest	-0.0039198	0.0136012	-0.2881939
low_features	presenting_complaintCirculation / chest >> Chest pain	-0.0036128	0.0126306	-0.2860343
low_features	presenting_complaintCirculation / chest >> Collapse / fainting episode	-0.0034496	0.0134565	-0.2563495
low_features	presenting_complaintCirculation / chest >> Palpitations	-0.0159501	0.0135218	-1.1795802
low_features	presenting_complaintEnvironmental	-0.0103460	0.0182349	-0.5673727
low_features	presenting_complaintEye	-0.0055898	0.0154785	-0.3611343
low_features	presenting_complaintGastrointestinal	0.0120873	0.0127160	0.9505547
low_features	presenting_complaintGastrointestinal >> Abdominal pain	0.0072924	0.0126290	0.5774349
low_features	presenting_complaintGeneral / minor / admin	0.0112914	0.0129219	0.8738228
low_features	presenting_complaintGeneral / minor / admin >> Generalised weakness	0.0098381	0.0126733	0.7762826
low_features	presenting_complaintGenitourinary	0.0052526	0.0127134	0.4131526
low_features	presenting_complaintGenitourinary >> Unable to pass urine	0.0019423	0.0130614	0.1487063
low_features	presenting_complaintHead and neck	-0.0062528	0.0136891	-0.4567686
low_features	presenting_complaintNeurological	-0.0023413	0.0130911	-0.1788494
low_features	presenting_complaintNeurological >> Confusion	-0.0001618	0.0130399	-0.0124084
low_features	presenting_complaintNeurological >> Dizziness	-0.0121107	0.0129961	-0.9318759
low_features	presenting_complaintNeurological >> Falls / unsteady on feet	0.0006513	0.0126368	0.0515424
low_features	presenting_complaintNeurological >> Headache	-0.0051691	0.0132317	-0.3906610
low_features	presenting_complaintNeurological >> Limb weakness	-0.0159479	0.0138598	-1.1506597
low_features	presenting_complaintNeurological >> Speech disturbance	0.0043205	0.0145238	0.2974798
low_features	presenting_complaintObGyn	-0.0090121	0.0192335	-0.4685610
low_features	presenting_complaintOther	0.0133619	0.0131981	1.0124098
low_features	presenting_complaintPsychosocial / Behaviour change	0.0003230	0.0141501	0.0228258
low_features	presenting_complaintSkin	-0.0028001	0.0143797	-0.1947292
low_features	presenting_complaintSkin >> Localised swelling / redness / lumps / bumps	0.0121323	0.0133330	0.9099475
low_features	presenting_complaintTrauma / musculoskeletal	0.0028566	0.0128168	0.2228836
low_features	presenting_complaintTrauma / musculoskeletal >> Head injury	0.0008286	0.0130289	0.0636007
low_features	presenting_complaintTrauma / musculoskeletal >> Injury of lower limb	0.0019624	0.0134982	0.1453839
low_features	presenting_complaintTrauma / musculoskeletal >> Pain in lower limb	-0.0019547	0.0128971	-0.1515621
low_features	presenting_complaintUnwell Adult	0.0222662	0.0136180	1.6350546
low_features	news_scaled	0.0057839	0.0006868	8.4220273
low_features	fi_acute_scaled	0.0264411	0.0007555	35.0001447
nurse	(Intercept)	4.2481979	0.2626052	16.1771291
nurse	femaleTRUE	0.0536516	0.0335613	1.5986162
nurse	age_scaled	0.5259654	0.0170998	30.7585714
nurse	PRUHTRUE	-1.0665806	0.0419078	-25.4506458
nurse	presenting_complaintAirway / breathing >> Difficulty breathing	0.1377086	0.2785602	0.4943586

nurse	presenting_complaintAirway / breathing >> Short of breath	-0.0103998	0.2644776	-0.0393220
nurse	presenting_complaintCirculation / chest	0.1572582	0.2819988	0.5576557
nurse	presenting_complaintCirculation / chest >> Chest pain	-0.2703990	0.2651883	-1.0196492
nurse	presenting_complaintCirculation / chest >> Collapse / fainting episode	0.0241385	0.2792671	0.0864350
nurse	presenting_complaintCirculation / chest >> Palpitations	-0.4485451	0.2800260	-1.6017983
nurse	presenting_complaintEnvironmental	0.1224257	0.3634904	0.3368058
nurse	presenting_complaintEye	-0.1235640	0.3245705	-0.3807002
nurse	presenting_complaintGastrointestinal	0.1550405	0.2666807	0.5813711
nurse	presenting_complaintGastrointestinal >> Abdominal pain	-0.1095034	0.2651892	-0.4129255
nurse	presenting_complaintGeneral / minor / admin	0.2588696	0.2702774	0.9577920
nurse	presenting_complaintGeneral / minor / admin >> Generalised weakness	0.3328588	0.2660209	1.2512503
nurse	presenting_complaintGenitourinary	0.1625812	0.2663907	0.6103108
nurse	presenting_complaintGenitourinary >> Unable to pass urine	0.1358385	0.2720412	0.4993307
nurse	presenting_complaintHead and neck	-0.1845260	0.2851491	-0.6471209
nurse	presenting_complaintNeurological	0.5370165	0.2730120	1.9670073
nurse	presenting_complaintNeurological >> Confusion	0.4453922	0.2716885	1.6393489
nurse	presenting_complaintNeurological >> Dizziness	-0.1463701	0.2712972	-0.5395192
nurse	presenting_complaintNeurological >> Falls / unsteady on feet	0.5105537	0.2651759	1.9253395
nurse	presenting_complaintNeurological >> Headache	-0.2431045	0.2754220	-0.8826619
nurse	presenting_complaintNeurological >> Limb weakness	0.2103929	0.2875179	0.7317556
nurse	presenting_complaintNeurological >> Speech disturbance	0.2613003	0.3003868	0.8698794
nurse	presenting_complaintObGyn	0.5076110	0.3873463	1.3104836
nurse	presenting_complaintOther	-0.2344949	0.2763201	-0.8486349
nurse	presenting_complaintPsychosocial / Behaviour change	-0.2230972	0.2900060	-0.7692848
nurse	presenting_complaintSkin	-0.0199786	0.2958265	-0.0675348
nurse	presenting_complaintSkin >> Localised swelling / redness / lumps / bumps	-0.0150400	0.2777285	-0.0541538
nurse	presenting_complaintTrauma / musculoskeletal	0.1933031	0.2682913	0.7204970
nurse	presenting_complaintTrauma / musculoskeletal >> Head injury	0.1505438	0.2712291	0.5550432
nurse	presenting_complaintTrauma / musculoskeletal >> Injury of lower limb	0.1925311	0.2799363	0.6877673
nurse	presenting_complaintTrauma / musculoskeletal >> Pain in lower limb	0.1669214	0.2699122	0.6184285
nurse	presenting_complaintUnwell Adult	-0.0890308	0.2869145	-0.3103044
nurse	news_scaled	0.1376012	0.0127939	10.7552561
nurse	fi_acute_scaled	0.1954820	0.0140868	13.8769638
drug	(Intercept)	0.2642537	0.0137300	19.2463993
drug	femaleTRUE	-0.0208862	0.0024190	-8.6342029
drug	age_scaled	-0.0026468	0.0012048	-2.1967819
drug	PRUHTRUE	0.0078994	0.0029900	2.6419119
drug	presenting_complaintAirway / breathing >> Difficulty breathing	-0.0137775	0.0143495	-0.9601406
drug	presenting_complaintAirway / breathing >> Short of breath	-0.0200628	0.0137811	-1.4558128
drug	presenting_complaintCirculation / chest	-0.0334038	0.0147568	-2.2636303
drug	presenting_complaintCirculation / chest >> Chest pain	-0.0230947	0.0139669	-1.6535332
drug	presenting_complaintCirculation / chest >> Collapse / fainting episode	-0.0368735	0.0147725	-2.4960885
drug	presenting_complaintCirculation / chest >> Palpitations	-0.0311979	0.0156473	-1.9938145
drug	presenting_complaintEnvironmental	-0.0182107	0.0206213	-0.8830982
drug	presenting_complaintEye	-0.0425888	0.0202501	-2.1031415
drug	presenting_complaintGastrointestinal	-0.0208776	0.0138597	-1.5063533
drug	presenting_complaintGastrointestinal >> Abdominal pain	-0.0214835	0.0138965	-1.5459644
drug	presenting_complaintGeneral / minor / admin	-0.0163394	0.0140704	-1.1612584
drug	presenting_complaintGeneral / minor / admin >> Generalised weakness	-0.0197536	0.0138920	-1.4219410
drug	presenting_complaintGenitourinary	-0.0193007	0.0140263	-1.3760332
drug	presenting_complaintGenitourinary >> Unable to pass urine	-0.0232472	0.0149804	-1.5518424
drug	presenting_complaintHead and neck	-0.0221121	0.0169486	-1.3046560
drug	presenting_complaintNeurological	-0.0230610	0.0143260	-1.6097286
drug	presenting_complaintNeurological >> Confusion	-0.0281285	0.0140803	-1.9977265
drug	presenting_complaintNeurological >> Dizziness	-0.0249979	0.0143413	-1.7430778
drug	presenting_complaintNeurological >> Falls / unsteady on feet	-0.0265468	0.0138216	-1.9206679
drug	presenting_complaintNeurological >> Headache	-0.0275551	0.0152763	-1.8037763
drug	presenting_complaintNeurological >> Limb weakness	-0.0261152	0.0151671	-1.7218313
drug	presenting_complaintNeurological >> Speech disturbance	-0.0138083	0.0156522	-0.8821945
drug	presenting_complaintObGyn	-0.0246923	0.0241552	-1.0222367
drug	presenting_complaintOther	-0.0355451	0.0145365	-2.4452272
drug	presenting_complaintPsychosocial / Behaviour change	-0.0303143	0.0153870	-1.9701270
drug	presenting_complaintSkin	-0.0391746	0.0171038	-2.2904094
drug	presenting_complaintSkin >> Localised swelling / redness / lumps / bumps	-0.0110909	0.0149329	-0.7427123
drug	presenting_complaintTrauma / musculoskeletal	-0.0230387	0.0140763	-1.6367057
drug	presenting_complaintTrauma / musculoskeletal >> Head injury	-0.0244436	0.0143280	-1.7060055

```
|drug          |presenting_complaintTrauma / musculoskeletal >> Injury of lower limb     | -0.0274414| 0.0143703|  -1.9095942|
|drug          |presenting_complaintTrauma / musculoskeletal >> Pain in lower limb       | -0.0251267| 0.0142347|  -1.7651762|
|drug          |presenting_complaintUnwell Adult                                         | -0.0253202| 0.0145094|  -1.7450956|
|drug          |news_scaled                                                              |  0.0018077| 0.0006460|   2.7981125|
|drug          |fi_acute_scaled                                                          |  0.0167346| 0.0008265|  20.2479193|
```

In [62]:
```
effects_summary_simple %>% write_csv('effect_summary_simple.csv')
effects_summary_complex %>% write_csv('effect_summary_complex.csv')
```

In [63]:
```
library(tableone)
```

In [64]:
```
map_ethnicity_function <- function(nationality_code) {
  ons_ethnicity <- case_when(
    nationality_code %in% c('White British', 'White Irish', 'White Scottish', 'White English', 'White Welsh', 'White Cornish',
                            'White and Black Caribben', 'White and Asian', 'White and Black African', 'White Nothern Irish',
                            'Other White Unspecified', 'Any other white back ground', 'Polish', 'Portuguese', 'Greek',
                            'Greek Cypriot', 'Cypriot (Part not stated)', 'Turkish Cypriot', 'Kosovan', 'All former USSR rep',
                            'Serbian', 'Croatian', 'Bosnian', 'Spanish', 'Italian', 'Albanian') ~ 'White',
    nationality_code %in% c('Black Caribbean', 'Black African', 'Black British', 'Any other Black background', 'Ghanaian',
                            'Nigerian', 'Other Black Unspecified', 'Black and Asian', 'Black and Chinese', 'Mixed Black',
                            'Mixed Asian', 'Black and White', 'Sudanese', 'Eritrean', 'Angolan', 'Somali') ~ 'Black',
    nationality_code %in% c('Asian/Chinese', 'Indian/British Indian', 'Pakistani/British Pakistani', 'Bangladeshi/British Bangladeshi',
                            'Other Asian Unspecified', 'Any other Asian background', 'East African Asian', 'Sri Lankan',
                            'Vietnamese', 'Punjabi', 'Sinhalese', 'Tamil', 'Chinese', 'Malaysian', 'Filipino', 'Japanese') ~ 'Asian',
    nationality_code %in% c('Mixed Black', 'Mixed Asian', 'Other Mixed', 'White and Black African', 'White and Black Caribbean',
                            'Chinese and White', 'Black and White') ~ 'Mixed',
    nationality_code %in% c('Arab', 'Middle East', 'Kurdish', 'Iranian', 'Iraqi', 'Ethiopian', 'Traveller', 'Gypsy/Romany',
                            'Other Latin American', 'Colombian', 'Ecuadorian') ~ 'Other',
    TRUE ~ 'Not Stated' # Catch-all for unspecified or unclear categories
  )}
```

In [ ]:
```
# First, let's create a function to count unique patients
count_unique_patients <- function(data) {
  length(unique(data$client_idcode))
}

# Function to create the comparison table
create_comparison_table <- function(results) {
  # Create a list to store each dataset's summary
  datasets_list <- list(
    base = results$base$datasets$los,
    short_period = results$short_period$datasets$los,
    mean_type = results$mean_type$datasets$los,
    high_features = results$high_features$datasets$los,
    low_features = results$low_features$datasets$los,
    nurse = results$nurse$datasets$los,
    drug = results$drug$datasets$los
  )

  # Define variables for comparison
  factorvars <- c('female', 'ons_ethnicity', 'PRUH', 'Alive', 'Group')
  continuous_vars <- c('age', 'NEWS_Score', 'imd_rank', 'imd_decile', 'los', 'chron_filab', 'adjusted_score', 'fi_acute')

  # Create list to store TableOne objects
  tables <- list()

  # Process each dataset
  for(name in names(datasets_list)) {
    data <- datasets_list[[name]] %>%
      mutate(
        ons_ethnicity = map_ethnicity_function(patient_NationalityCode),
        Unique_patients = count_unique_patients(.), # Changed to . since we're in a pipe
        # Alive is already in your data so we don't need to create it
        Group = name # Add group identifier
      )
```

```
    # Create TableOne
    tables[[name]] <- CreateTableOne(
      vars = c(factorvars, continuous_vars, 'Unique_patients'),
      data = data,
      factorVars = factorvars,
      includeNA = TRUE,
      test = TRUE
    )
  }

  # Combine all tables
  comparison_table <- print(
    tables,
    printToggle = FALSE,
    showAllLevels = TRUE,
    nonnormal = continuous_vars
  )

  return(comparison_table)
}

# Load necessary libraries if not already loaded
library(tableone)
library(dplyr)

# Create the comparison table
comparison_results2 <- create_comparison_table(results)
```

In [66]:

```
comparison_results2
```

```
$base

                              Overall
  n                              60381
  female = TRUE (%)              32260 ( 53.4)
  ons_ethnicity (%)
     Asian                        2482 (  4.1)
     Black                       10894 ( 18.0)
     Mixed                          33 (  0.1)
     Not Stated                   4263 (  7.1)
     Other                         346 (  0.6)
     White                       42363 ( 70.2)
  PRUH = TRUE (%)                22331 ( 37.0)
  Alive = TRUE (%)               40070 ( 66.4)
  Group = base (%)               60381 (100.0)
  age (mean (SD))                81.64 (7.01)
  NEWS_Score (mean (SD))          1.96 (1.31)
  imd_rank (mean (SD))        15685.58 (8683.95)
  imd_decile (mean (SD))          5.26 (2.63)
  los (mean (SD))                 4.28 (9.75)
  chron_filab (mean (SD))         0.17 (0.13)
  adjusted_score (mean (SD))      4.06 (1.87)
  fi_acute (mean (SD))            0.39 (0.11)
  Unique_patients (mean (SD)) 23956.00 (0.00)

$short_period

                              Overall
  n                              60381
  female = TRUE (%)              32260 ( 53.4)
  ons_ethnicity (%)
     Asian                        2482 (  4.1)
     Black                       10894 ( 18.0)
     Mixed                          33 (  0.1)
     Not Stated                   4263 (  7.1)
     Other                         346 (  0.6)
     White                       42363 ( 70.2)
  PRUH = TRUE (%)                22331 ( 37.0)
  Alive = TRUE (%)               40070 ( 66.4)
  Group = short_period (%)       60381 (100.0)
  age (mean (SD))                81.64 (7.01)
  NEWS_Score (mean (SD))          1.96 (1.31)
  imd_rank (mean (SD))        15685.58 (8683.95)
  imd_decile (mean (SD))          5.26 (2.63)
  los (mean (SD))                 4.28 (9.75)
  chron_filab (mean (SD))         0.17 (0.13)
  adjusted_score (mean (SD))      4.06 (1.87)
  fi_acute (mean (SD))            0.39 (0.11)
  Unique_patients (mean (SD)) 23956.00 (0.00)

$mean_type

                              Overall
  n                              60381
  female = TRUE (%)              32260 ( 53.4)
  ons_ethnicity (%)
     Asian                        2482 (  4.1)
     Black                       10894 ( 18.0)
     Mixed                          33 (  0.1)
     Not Stated                   4263 (  7.1)
     Other                         346 (  0.6)
     White                       42363 ( 70.2)
  PRUH = TRUE (%)                22331 ( 37.0)
  Alive = TRUE (%)               40070 ( 66.4)
  Group = mean_type (%)          60381 (100.0)
  age (mean (SD))                81.64 (7.01)
```

```
  NEWS_Score (mean (SD))          1.96 (1.31)
  imd_rank (mean (SD))        15685.58 (8683.95)
  imd_decile (mean (SD))          5.26 (2.63)
  los (mean (SD))                 4.28 (9.75)
  chron_filab (mean (SD))         0.19 (0.14)
  adjusted_score (mean (SD))      4.06 (1.87)
  fi_acute (mean (SD))            0.39 (0.11)
  Unique_patients (mean (SD)) 23956.00 (0.00)

$high_features

                              Overall
  n                              36267
  female = TRUE (%)              19251 ( 53.1)
  ons_ethnicity (%)
     Asian                        1722 (  4.7)
     Black                        8496 ( 23.4)
     Mixed                          23 (  0.1)
     Not Stated                   1979 (  5.5)
     Other                         278 (  0.8)
     White                       23769 ( 65.5)
  PRUH = TRUE (%)                 6714 ( 18.5)
  Alive = TRUE (%)               24103 ( 66.5)
  Group = high_features (%)      36267 (100.0)
  age (mean (SD))                81.30 (6.88)
  NEWS_Score (mean (SD))          1.98 (1.33)
  imd_rank (mean (SD))        13777.78 (7798.56)
  imd_decile (mean (SD))          4.69 (2.37)
  los (mean (SD))                 4.13 (9.67)
  chron_filab (mean (SD))         0.15 (0.13)
  adjusted_score (mean (SD))      4.20 (1.85)
  fi_acute (mean (SD))            0.39 (0.11)
  Unique_patients (mean (SD)) 12524.00 (0.00)

$low_features

                              Overall
  n                              74493
  female = TRUE (%)              40496 ( 54.4)
  ons_ethnicity (%)
     Asian                        2909 (  3.9)
     Black                       12014 ( 16.1)
     Mixed                          40 (  0.1)
     Not Stated                   7058 (  9.5)
     Other                         387 (  0.5)
     White                       52085 ( 69.9)
  PRUH = TRUE (%)                30687 ( 41.2)
  Alive = TRUE (%)               49923 ( 67.0)
  Group = low_features (%)       74493 (100.0)
  age (mean (SD))                81.73 (7.11)
  NEWS_Score (mean (SD))          1.95 (1.30)
  imd_rank (mean (SD))        16109.21 (8787.37)
  imd_decile (mean (SD))          5.39 (2.66)
  los (mean (SD))                 4.37 (9.96)
  chron_filab (mean (SD))         0.19 (0.13)
  adjusted_score (mean (SD))      4.01 (1.87)
  fi_acute (mean (SD))            0.39 (0.11)
  Unique_patients (mean (SD)) 31353.00 (0.00)

$nurse

                              Overall
  n                              60381
  female = TRUE (%)              32260 ( 53.4)
  ons_ethnicity (%)
     Asian                        2482 (  4.1)
```

```
    Black                          10894 ( 18.0)
    Mixed                             33 (  0.1)
    Not Stated                      4263 (  7.1)
    Other                            346 (  0.6)
    White                          42363 ( 70.2)
  PRUH = TRUE (%)                  22331 ( 37.0)
  Alive = TRUE (%)                 40070 ( 66.4)
  Group = nurse (%)                60381 (100.0)
  age (mean (SD))                  81.64 (7.01)
  NEWS_Score (mean (SD))            1.96 (1.31)
  imd_rank (mean (SD))          15685.58 (8683.95)
  imd_decile (mean (SD))            5.26 (2.63)
  los (mean (SD))                   4.28 (9.75)
  chron_filab (mean (SD))           0.17 (0.13)
  adjusted_score (mean (SD))        4.06 (1.87)
  fi_acute (mean (SD))              0.39 (0.11)
  Unique_patients (mean (SD)) 23956.00 (0.00)

$drug

                              Overall
  n                            60381
  female = TRUE (%)            32260 ( 53.4)
  ons_ethnicity (%)
    Asian                       2482 (  4.1)
    Black                      10894 ( 18.0)
    Mixed                         33 (  0.1)
    Not Stated                  4263 (  7.1)
    Other                        346 (  0.6)
    White                      42363 ( 70.2)
  PRUH = TRUE (%)              22331 ( 37.0)
  Alive = TRUE (%)             40070 ( 66.4)
  Group = drug (%)             60381 (100.0)
  age (mean (SD))              81.64 (7.01)
  NEWS_Score (mean (SD))        1.96 (1.31)
  imd_rank (mean (SD))      15685.58 (8683.95)
  imd_decile (mean (SD))        5.26 (2.63)
  los (mean (SD))               4.28 (9.75)
  chron_filab (mean (SD))       0.17 (0.13)
  adjusted_score (mean (SD))    4.06 (1.87)
  fi_acute (mean (SD))          0.39 (0.11)
  Unique_patients (mean (SD)) 23956.00 (0.00)
```

In [67]:

```
# Get all the tables from comparison_results and combine them side by side
row_names <- rownames(comparison_results[[1]])
combined_table <- do.call(cbind, comparison_results)
rownames(combined_table) <- row_names

# Add group names to column headers if desired
current_colnames <- colnames(combined_table)
new_colnames <- current_colnames
for(name in names(comparison_results)) {
  cols_for_group <- grep(paste0("^p\\.value|^test|^\\.|^n$"),
                        colnames(comparison_results[[name]]),
                        invert = TRUE)
  new_colnames[which(current_colnames %in% colnames(comparison_results[[name]])[cols_for_group])] <-
    paste(name, current_colnames[which(current_colnames %in% colnames(comparison_results[[name]])[cols_for_group])],
          sep = "_")
}
colnames(combined_table) <- new_colnames

# View or save the combined table
head(combined_table)
# write.csv(combined_table, "comparison_table.csv")
```
