# Supplementary material for: Can laboratory test-based frailty indices contribute to frailty screening in emergency departments?
Source: Age Ageing. 2025 Jul 16;54(7):afaf192. doi: 10.1093/ageing/afaf192 (PMC12264206; doi:10.1093/ageing/afaf192)
Supplement: aa-25-0992-File003_afaf192 [file aa-25-0992-file003_afaf192.docx]

STROBE Statement—checklist of items that should be included in reports of observational studies

|  | Item No. | Recommendation | Page  No. | Relevant text from manuscript |
| --- | --- | --- | --- | --- |
| **Title and abstract** | 1 | (*a*) Indicate the study’s design with a commonly used term in the title or the abstract | 1 | In this retrospective cohort study |
|  |  | (*b*) Provide in the abstract an informative and balanced summary of what was done and what was found | 1 |  |
| Introduction | | | |  |
| Background/rationale | 2 | Explain the scientific background and rationale for the investigation being reported | 2-3 | Introduction section |
| Objectives | 3 | State specific objectives, including any prespecified hypotheses | 3 | Primary objectives stated: 'Compare the performance and reliability of laboratory-based frailty indices (FI-Labs) with nurse-assessed Clinical Frailty Scale (CFS) scores...' and 'Evaluate how different configurations of the FI-Lab influence their associations with adverse clinical outcomes...'." |
| Methods | | | |  |
| Study design | 4 | Present key elements of study design early in the paper | 4 | We conducted a retrospective cohort study...' |
| Setting | 5 | Describe the setting, locations, and relevant dates, including periods of recruitment, exposure, follow-up, and data collection | 4 | Described setting: '...two London hospitals: King's College Hospital (KCH) and Princess Royal University Hospital (PRUH)'. Dates: '...ED attendances between July 2017 and December 2021'. Data collection period implied by study dates. |
| Participants | 6 | (*a*) *Cohort study*—Give the eligibility criteria, and the sources and methods of selection of participants. Describe methods of follow-up  *Case-control study*—Give the eligibility criteria, and the sources and methods of case ascertainment and control selection. Give the rationale for the choice of cases and controls  *Cross-sectional study*—Give the eligibility criteria, and the sources and methods of selection of participants | 4 | "Eligibility criteria: 'patients aged 70 years or older' in ED. Sources/methods of selection: All eligible ED attendances from EHRs of two hospitals during the study period. Exclusions: 'indeterminate gender and those without complete attendance records (70 patients in total)'. Follow-up methods: Outcomes (mortality etc.) ascertained via EHR linkage up to study end |
|  |  | (*b*) *Cohort study*—For matched studies, give matching criteria and number of exposed and unexposed  *Case-control study*—For matched studies, give matching criteria and the number of controls per case |  |  |
| Variables | 7 | Clearly define all outcomes, exposures, predictors, potential confounders, and effect modifiers. Give diagnostic criteria, if applicable | 7 | "Outcomes: time to discharge, death during admission, 90-day mortality, overall survival. Exposures/predictors: Nurse-assessed CFS, 6 FI-Lab versions (Base, Short-period, Mean-type, High-features, Low-features, Drug-adjusted) detailed. Potential confounders: Age, sex, hospital site, NEWS score, socioeconomic status (IMD), specific lab markers." |
| Data sources/ measurement | 8* | For each variable of interest, give sources of data and details of methods of assessment (measurement). Describe comparability of assessment methods if there is more than one group | *7* | *Data source: EHRs via CogStack. Variables measured: FI-Labs calculated from aggregated monthly lab data (details provided, e.g., 36-month window for Base FI-Lab, proportion outside range) ; Nurse CFS assessed at triage; Demographics, vitals, NEWS, medications, outcomes extracted.* |
| Bias | 9 | Describe any efforts to address potential sources of bias | 4, 15, 16 + supplemental | Monthly aggregation of lab data; Exclusion of admission month lab data for FI-Lab calculation. Potential biases discussed: Limited generalisability; Selection bias from differing data requirements for FI-Lab versions emporal aggregation bias; Observational design limits causal inference |
| Study size | 10 | Explain how the study size was arrived at | 8 | Total attendances (74,493) and unique patients (54,075) reported. Sample sizes vary by FI-Lab configuration due to data requirements (e.g., High-features N=36,267; Low-features N=74,493). Size determined by available retrospective data in the timeframe. |

Continued on next page

| Quantitative variables | 11 | Explain how quantitative variables were handled in the analyses. If applicable, describe which groupings were chosen and why | 4, 5 | Lab data aggregated monthly. FI-Lab calculation methods described (proportion outside range, mean value outside range). Continuous frailty measures standardized (per SD) for hazard ratio comparison. Used in Cox and mixed-effects models." |
| --- | --- | --- | --- | --- |
| Statistical methods | 12 | (*a*) Describe all statistical methods, including those used to control for confounding | 7 | Survival analysis using Cox proportional hazards models for outcomes. Basic models adjusted for age, sex, site; Complex models added NEWS, socioeconomic status, lab markers. Reliability assessed using linear mixed-effects models with random intercepts for patient ID, adjusted for age, sex, site, presenting complaint. |
|  |  | (*b*) Describe any methods used to examine subgroups and interactions | 4,5 | Comparison of different FI-Lab configurations.. |
|  |  | (*c*) Explain how missing data were addressed | 7 | Participants with missing data for any covariates included in the complex models were excluded from those specific analyses. |
|  |  | (*d*) *Cohort study*—If applicable, explain how loss to follow-up was addressed  *Case-control study*—If applicable, explain how matching of cases and controls was addressed  *Cross-sectional study*—If applicable, describe analytical methods taking account of sampling strategy | 7 | Loss to follow-up handled via survival analysis with defined outcome periods (in-hospital, 90-day, overall survival). Repeated measures handled using mixed-effects models with random intercepts for patient ID |
|  |  | (*e*) Describe any sensitivity analyses | N/A | Not performed – the whole paper could be considered a set of sensitivity analyses |
| Results | | | | |
| Participants | 13* | (a) Report numbers of individuals at each stage of study—eg numbers potentially eligible, examined for eligibility, confirmed eligible, included in the study, completing follow-up, and analysed | 8 | Total attendances = 74,493; Unique patients = 54,075. Numbers included for each FI-Lab configuration reported (e.g., Base FI-Lab N=60,381; High-features N=36,267) |
|  |  | (b) Give reasons for non-participation at each stage | 4 | Excluded 70 patients (indeterminate gender/incomplete records). Reasons for varying N in analyses: differing minimum data requirements for each FI-Lab configuratio |
|  |  | (c) Consider use of a flow diagram |  |  |
| Descriptive data | 14* | (a) Give characteristics of study participants (eg demographic, clinical, social) and information on exposures and potential confounders | Table 2 |  |
|  |  | (b) Indicate number of participants with missing data for each variable of interest | Supplemental material |  |
|  |  | (c) *Cohort study*—Summarise follow-up time (eg, average and total amount) | Supplemental Material |  |
| Outcome data | 15* | *Cohort study*—Report numbers of outcome events or summary measures over time | Table 2 |  |
|  |  | *Case-control study—*Report numbers in each exposure category, or summary measures of exposure |  |  |
|  |  | *Cross-sectional study—*Report numbers of outcome events or summary measures |  |  |
| Main results | 16 | (*a*) Give unadjusted estimates and, if applicable, confounder-adjusted estimates and their precision (eg, 95% confidence interval). Make clear which confounders were adjusted for and why they were included | Table 3 | Adjusted HRs and 95% CIs provided in Table 3. Complex models adjusted for age, sex, site, NEWS, socioeconomic status, lab markers are presented. |
|  |  | (*b*) Report category boundaries when continuous variables were categorized |  |  |
|  |  | (*c*) If relevant, consider translating estimates of relative risk into absolute risk for a meaningful time period |  |  |

Continued on next page

| Other analyses | 17 | Report other analyses done—eg analyses of subgroups and interactions, and sensitivity analyses |  | Reliability analysis using linear mixed-effects models and ICC reported (e.g., Drug-adjusted FI-Lab ICC=0.74). Analysis of influence of presenting complaints and NEWS score on different measures. Model fit comparisons using AIC/BIC. |
| --- | --- | --- | --- | --- |
| Discussion | | | | |
| Key results | 18 | Summarise key results with reference to study objectives | 14 | Our FI-Labs showed inferior predictive validity compared to nurse-assessed CFS scores but exhibited substantially greater between-visit reliability (Objective 1), while different FI-Lab configurations showed only minor variations in their associations with clinical outcomes, with increased complexity often resulted in reduced population coverage (Objective 2). |
| Limitations | 19 | Discuss limitations of the study, taking into account sources of potential bias or imprecision. Discuss both direction and magnitude of any potential bias | 15,16, supplemental |  |
| Interpretation | 20 | Give a cautious overall interpretation of results considering objectives, limitations, multiplicity of analyses, results from similar studies, and other relevant evidence | 14,15,16 |  |
| Generalisability | 21 | Discuss the generalisability (external validity) of the study results | 16 | Generalisability (external validity) discussed as a limitation due to the study being conducted in two London hospitals. Notes findings might not apply to other settings with different populations or practices |
| Other information | |  | | |
| Funding | 22 | Give the source of funding and the role of the funders for the present study and, if applicable, for the original study on which the present article is based | 8 | Dalhousie Department of Medicine Research Fellowship and King's College London Centre for Doctoral Studies (to HLE)'. Role of funders stated: 'The funders had no role in study design, data collection and analysis, decision to publish, or preparation of the manuscript'. |

*Give information separately for cases and controls in case-control studies and, if applicable, for exposed and unexposed groups in cohort and cross-sectional studies.

**Note:** An Explanation and Elaboration article discusses each checklist item and gives methodological background and published examples of transparent reporting. The STROBE checklist is best used in conjunction with this article (freely available on the Web sites of PLoS Medicine at http://www.plosmedicine.org/, Annals of Internal Medicine at http://www.annals.org/, and Epidemiology at http://www.epidem.com/). Information on the STROBE Initiative is available at www.strobe-statement.org.
